# Integrative Analysis and Experimental Validation Indicated That SNHG17 Is a Prognostic Marker in Prostate Cancer and a Modulator of the Tumor Microenvironment via a Competitive Endogenous RNA Regulatory Network

**DOI:** 10.1155/2022/1747604

**Published:** 2022-07-12

**Authors:** Jinling Li, Hongyuan Yu, Jinlong Yao, Zhenming Jiang, Zhenhua Li, Xiaolu Cui

**Affiliations:** ^1^Department of Urology, First Hospital of China Medical University, Shenyang 110001, China; ^2^Department of Urology, Shengjing Hospital of China Medical University, Shenyang 110004, China

## Abstract

The incidence of prostate cancer (PC) is growing rapidly worldwide, and studies uncovering the molecular mechanisms driving the progression and modulating the immune infiltration and antitumor immunity of PC are urgently needed. The long noncoding RNA SNHG family has been recognized as a prognostic marker in cancers and contributes to the progression of multiple cancers, including PC. In this study, we aimed to clarify the prognostic values and underlying mechanisms of SNHGs in promoting the progression and modulating the tumor microenvironment of PC through data mining based on The Cancer Genome Atlas (TCGA) database. We identified that within the SNHG family, SNHG17 was most correlated with the overall survival of PC patients and could act as an independent predictor. Moreover, we constructed a competitive endogenous RNA (ceRNA) network by which SNHG17 promotes progression and potentially inhibits the immune infiltration and immune response of prostate cancer. By interacting with miR-23a-3p/23b-3p/23c, SNHG17 upregulates the expression of UBE2M and OTUB1, which have been demonstrated to play critical roles in the tumorigenesis of human cancers, more importantly promoting cancer cell immunosuppression and resistance to cytotoxic stimulation. Finally, we examined the correlation between SNHG17 expression and the clinical progression of PC patients based on our cohort of 52 PC patients. We also verified the SNHG17/miR-23a/OTUB1 axis in RV-1 and PC-3 cells by dual luciferase and RIP assays, and we further identified that SNHG17 promoted cellular invasive capacity by modulating OTUB1. In summary, the current study conducted a ceRNA-based SNHG17-UBE2M/OTUB1 axis and indicated that SNHG17 might be a novel prognostic factor associated with the progression, immunosuppression, and cytotoxic resistance of PC.

## 1. Introduction

Prostate cancer (PC) is a major disease that threatens human health worldwide. According to the data from cancer statistics 2022, PC ranks 1^st^ of estimated new cases (268,490 cases, 27%) and 2^nd^ of estimated deaths (34,500 deaths, 11%) in the U.S. [1]. In China, the estimated number of prostate cancer patients in 2022 will be 125,646 in total [2]. According to data from the World Health Organization, the future burden of PC in China will be over 20/million, and the death rate will be 12/million in 2040. This suggests that early diagnosis and effective treatment for prostate cancer have become far more important than ever. Next-generation genome sequencing and gene/protein expression profile analysis of prostate tumors have uncovered the potential importance of genetic and epigenetic changes observed in the infiltration of immune cells in the tumor microenvironment and the genetic heterogeneity in prostate cancer cells. As a result, the treatment strategies for PC and castration-resistant prostate cancer (CRPC) have entered the era of molecular immunotherapy that includes CAR-T therapy [3] and immune checkpoint blockade (ICB) [4].

In the past few decades, numerous studies have helped to uncover the molecular and biological processes that initiate the progression of PC. In particular, with the tools of whole transcriptome and exon sequencing, biologists have deciphered various genomic alterations that contribute to the pathogenesis of PC. Transcriptome sequencing data reveal that 70-90% of human genes are involved in genomic transcription; however, only 2% of transcripts encode proteins, while the others are mostly noncoding RNAs. Long noncoding RNAs (lncRNAs) have been identified as key regulatory molecules in multiple biological processes, including tumorigenesis. The small nucleolar RNA host gene (SNHG) family is a subgroup of lncRNAs that are reported to be dysregulated in various human cancers [5]. SNHGs have been recognized to improve cell proliferation, cell cycle progression, invasion and metastasis of urological cancer cells [6]. For example, in renal cell carcinoma, SNHG12 interacts with microRNA- (miRNA-) 30a-3p and consequently promotes the expression of the downstream oncogenes RUNX2, IGF-1R, and WNT2 [7]. SNHG1 was reported to play an oncogenetic role in prostate cancer via the ceRNA networks of miR-199a-3p/CDK7 [8] and miR-377-3p/AKT2 [9]. SNHG5 has been identified to improve the proliferation, cell cycle progression, and invasion of bladder cancer cells through the miR-363-3p/Twist1 axis [10]. Accumulating studies have focused on the biological functions and underlying mechanisms of SNHGs driving the progression of cancer, including urological cancers.

Here, we investigated the prognostic value of SNHGs and analyzed the underlying mechanisms of SNHG17 in PC using bioinformatic analysis tools. First, we explored the prognostic value of 32 SNHGs based on the overall survival (OS) and progression-free interval (PFI) data of the PRAD cohort from The Cancer Genome Atlas (TCGA) databases, and we identified 6 SNHGs that positively correlated with both OS and PFI. Afterwards, we screened 8 significant prognostic SNHGs using the least absolute shrinkage and selection operator (LASSO) Cox regression. By comparing the two SNHG gene sets, SNHG17 was found to be the only common prognostic indicator. We next built a prognostic model for the OS of PC patients based on T stage, N stage, Gleason's score, and SNHG17 expression. We also evaluated the potential mechanisms involving the functions of SNHG17 in PC through Gene Ontology (GO), Kyoto Encyclopedia Genes and Genomes (KEGG), and gene set enrichment analysis (GSEA). Finally, we explored the competitive endogenous RNA (ceRNA) regulatory network of SNHG17 in PC. Ovarian tumor (OUT) deubiquitinase, ubiquitin aldehyde binding 1 (OTUB1) and ubiquitin conjugating enzyme E2 M (UBE2M) were predicted to be regulated by SNHG17 via competitively interacting with miR-23a-3p, miR-23b-3p, and miR-23c. The SNHG17/miR-23a-3p/OTUB1 axis was validated in RV-1 and PC-3 cells. Through this regulatory network, SNHG17 potentially modulates critical biological processes such as protein polyubiquitination, cell cycle, and autophagy in PC cells.

## 2. Materials and Methods

### 2.1. Clinical Tissue Specimens

Prior patients' written consent and approval were obtained from the First Hospital of China Medical University for the use of clinical specimens for research purposes. A total of 52 patients with prostate cancer who underwent radical cystectomy from 2015 to 2017 at the Department of Urology, First Hospital of China Medical University, were included in this study. Histologically, the tumors were classified according to the 2004 World Health Organization histologic classification of prostate cancer and were staged using the 2002 American Joint Committee on Cancer system. The use of the clinical specimens was approved by the ethics committee of the First Hospital of China Medical University.

### 2.2. Cell Culture

RV-1 and PC-3 cells were purchased from the Cell Bank of the Chinese Academy of Sciences (Shanghai, China). The experimental cells were maintained in RPMI 1640 medium supplemented with 10% FBS and antibiotics.

### 2.3. RNA Isolation, Primers, and Real-Time Quantitative PCR

RNA isolation and qRT–PCR assays were performed as described previously [11]. Total RNA was extracted from cultured cells using TRIzol reagent (Invitrogen) and reverse transcribed with random primers using PrimeScript™ RT Master Mix (Perfect Real Time; Takara Biotechnology Co. Ltd., Dalian, China) according to the manufacturer's instructions. For miR-23a-3p detection, cDNA synthesis and quantitative real-time PCR were performed using a mercury LNA™ Universal RT MicroRNA PCR kit (Exiqon, Skelstedet, Vedbaek, Denmark). qRT–PCR was performed using SYBR® Premix Ex Taq™ (Tli RNase H Plus; Takara Biotechnology Co. Ltd., Dalian, China) and LightCycler™ 480 II system (Roche, Basel, Switzerland). GAPDH and U6 snRNA were employed as endogenous controls for OTUB1/SNHG17 and miR-23a-3p, respectively. The relative levels of gene expression were quantified and analyzed using Light Cycler™ 480 software 1.5.1.6.2 (Roche, Basel, Switzerland). The real-time value for each sample was averaged and compared using the 2-*ΔΔ*Ct method. Three independent experiments were performed to analyze the relative gene expression. The primers for miRNAs were synthesized by Exiqon, and the primers for mRNAs were as follows: SNHG17-fw: 5′-TGCTTGTAAGGCAGGGTCTC-3′; SNHG17-rev: 5′-ACAGCCACTGAAAGCATGTG-3′; OTUB1-fw: 5′-ATGACCAGAGCACCTCCGACTACC-3′, OTUB1-rev: 5′-GACCATTTACAACCACAGAAAAAC-3′; GAPDH-fw: 5′-GAAGAGAGAGACCCTCACGCTG-3′; GAPDH-rev: 5′-ACTGTGAGGAGGGGAGATTCAGT-3.

### 2.4. Protein Extraction and Western Blotting Assay

An antibody against OTUB1 (ab270595) was purchased from Abcam and used according to the manufacturer's recommendations. Protein extraction and western blotting assays were performed as described previously [11]. The immune bands were visualized using ECL reagents (Transgen Biotechnology, Beijing, China) on a MicroChemi Chemiluminescent Imaging System (DNR Bio-Imaging Systems, Mahale HaHamisha, Jerusalem, Israel).

### 2.5. Immunochemistry Staining

Clinical pathological sections of prostate cancer tissue specimens from 6 PC patients were provided by the Department of Pathology of the First Hospital of China Medical University. The expression of OTUB1 in tissue specimens was detected using an UltraSensitive™ SP (Mouse/Rabbit) IHC kit (Maxin-Bio, Fuzhou, Fujian, China) according to the manufacturer's instructions. Immunochemistry staining was performed as described previously [11]. The images were captured by an upright metallurgical microscope (Olympus, Tokyo, Japan) under an original magnification of ×200. The cell-positive rate was counted in three random visual fields.

### 2.6. RNA Binding Protein Immunoprecipitation (RIP) Assay

RV-1 cells were lysed with TRIzol reagent (Invitrogen), and total RNA was extracted and prepared. One microgram of each RNA sample was incubated with 5 *μ*g of anti-IgG or anti-AGO2 antibody overnight at 4°C, and the complexes were isolated with magnetic beads (Invitrogen). The genes present in the pull-down products were measured by real-time PCR.

### 2.7. Dual Luciferase Assay

To test the interactions between SNHG17 and miR-23a-3p, luciferase reporter plasmids containing wild-type SNHG17 or miRNA response element- (MRE-) mutated SNHG17 were cotransfected with negative control or miR-23a-3p mimics into PC cells using Lipofectamine 3000 (Invitrogen). To test the interactions between miR-23a-3p and OTUB1, luciferase reporter plasmids containing the wild-type 3′UTR of OTUB1 or the MRE-mutated 3′UTR of OTUB1 were cotransfected with the negative control or miR-23a-3p mimics into PC cells. Luciferase reporter activity was measured using a Dual Luciferase Reporter Assay Kit (Promega) according to the manufacturer's protocol. The relative luciferase activity was measured by a Synergy HTX multimode microplate reader (BioTek).

### 2.8. Plasmid/Small Interfering RNA Construction and Transfection

Luciferase reporter plasmids (psiCHECK), OTUB1 overexpression plasmids (pcDNA3.1), siRNA against SNHG17, and miR-23a-3p mimics/inhibitors were synthesized by Genechem (Shanghai, China). Cell transfections were performed using Lipofectamine 3000 (Invitrogen) according to the manufacturer's protocol. Briefly, cells were cultured in a 6-well plate with 2 ml of culture medium. Luciferase plasmids (1 *μ*g), overexpression plasmids (1 *μ*g), or siRNA (75 nM) was added to the medium, and the medium was removed after 6 hours. Then, 2 ml of culture medium was added to each well, and the cells were subsequently cultured for another 24 hours.

### 2.9. Transwell Assay

Transwell assays were performed using a transwell chamber (Corning) and Matrigel (BD Biosciences), and the experimental protocol was as described previously [11]. The number of cells invaded through the gel was counted in three random visual fields, and the images were captured by a Leica DM3000 microscope (Leica).

### 2.10. Gene Ontology (GO) Term and Kyoto Encyclopedia of Genes and Genomes (KEGG) Pathway Enrichment Analysis and Gene Set Enrichment Analysis (GSEA)

To explore the biological functions of target genes, the data were analyzed by functional enrichment and GSEA. The positive and negative correlations of expression between all genes and SNHG17, OTUB1, or UBE2M were generated based on RNA sequencing data from TCGA. Data were analyzed using R software (V 3.6.3) and the DESeq2 package [12]. Gene Ontology (GO) is a tool for annotating genes with functions, including molecular functions (MF), biological pathways (BP), and cellular components (CC). KEGG is a collection of databases that includes the analysis of genomes, biological pathways, diseases, drugs, and chemical substances (http://www.kegg.jp/kegg/kegg1.html). GSEA is a computational method that allows the determination of classes of genes or proteins that are overrepresented in a large set of genes or proteins and may have a statistically significant association with disease phenotypes [13]. The defined gene sets are from the molecular signatures database (MSigDB) [14]. The enriched pathways were determined based on the nominal *p* value and the normalized enrichment score (NES).

### 2.11. Kaplan–Meier Analysis

Cox regression univariate analysis was used to analyze the correlation between SNHG expression and patient overall survival (OS) and progression-free interval (PFI) using TCGA database. The Kaplan–Meier (KM) method was used to analyze the correlation between the expression of potential genes and the survival of patients based on the best separation of potential gene expression using TCGA database. The prognostic value of SNHGs, miR-23a-3p, miR-23b-3p, and miR-23c in prostate cancer was assessed according to overall survival (OS) and progression-free interval (PFI) using Kaplan–Meier plotter. Kaplan–Meier analysis was conducted based on RNA sequence datasets as well as clinical survival data of prostate cancer patients from TCGA. Cox regression univariate analysis was performed using survival software, and the results were visualized by the “forest plot” R package using forest plots. The KM analysis and KM curve were calculated by R software 3.6.3, survminer package and survival package.

### 2.12. Data Collection and Processing

The present study was based on The Cancer Genome Atlas (TCGA) dataset [15]. TCGA (Table 1) was used to obtain gene expression information of tumor and normal samples and clinical information data and to analyze the expression of SNHGs, miR-23a-3p/23b/3p/23c, OTUB1, and UBE2M in prostate tumors. To examine the positively or negatively expressed genes with SNHG17, ORUB1, or UBE2M, RNA sequencing datasets of TCGA_PRAD were analyzed by R 3.6.3, DESeq2 package.

### 2.13. Immune Infiltration and Tumor Immune Estimation Resource (TIMER)

The immune infiltration signature of SNHG17 was analyzed by ssGSEA, and the correlation of the immune infiltrating level and UBE2M/OTUB1 was plotted by ssGSEA and Tumor Immune Estimation Resource (TIMER) [16]. The relative tumor infiltration levels of 24 immune cell types were quantified by ssGSEA to interrogate the expression levels of genes in published signature gene lists [17]. The TIMER database was used to evaluate the correlation between UBE2M/OTUB1 expression and the infiltration levels of six immune infiltrates.

### 2.14. UALCAN

UALCAN provides analyses of transcriptome data from The Cancer Genome Atlas (TCGA) and MET500 data [18]. In this study, UALCAN was used to investigate the association between the expression of UBE2M/OTUB1 and clinicopathological parameters (lymph node metastasis status, TP53 mutation status) and promoter methylation levels of UBE2M/OTUB1 in prostate cancer.

### 2.15. The Encyclopedia of RNA Interactomes

The Encyclopedia of RNA Interactomes (ENCORI) is a platform for studying miRNA-ncRNA, RBP-ncRNA, and RBP-mRNA interactions from CLIP-seq, degradome-seq, and RNA–RNA interactome data [19]. In this study, lncRNA-mRNA, miRNA–mRNA, and lncRNA-miRNA interactions were analyzed by ENCORI. The parameters for each analysis module were set as suggested by the software.

### 2.16. Statistical Analysis and Model Construction

Statistical analyses were performed by the R package (V3.6.3). The association between clinical pathologic features and SNHG17 expression was analyzed by the Wilcoxon signed-rank sum test and logistic regression. The association between clinicopathological characteristics and the overall survival (OS) and progression-free interval (PFI) in TCGA_PRAD patients was analyzed by Cox regression and the Kaplan–Meier method. Multivariate Cox analysis was used to analyze the impact of SNHG17 expression on patient survival as well as other clinical features (T stage, lymph node status, Gleason's score, distant metastasis status, and PSA level).

Based on Cox regression models and LASSO regression analysis, SNHG17 expression along with other independent prognostic factors was used to establish a nomogram [20], individualizing the predicted survival probability for 3-, 5-, and 7-year survival. The RMS package (version: 5.1-4; https://cran.r-project.org/web/packages/rms/index.html) was used to construct the nomogram. The calibration curves were graphically generated by mapping the nomogram-predicted probabilities against the observed occurrences. The chi-square test was used to compare the clinical and pathological characteristics of the low and high SNHG17 expression groups. The HR with 95% confidence interval (CI) was measured to estimate the hazard risk of individual factors. *p* < 0.05 indicates statistical significance, and *p* < 0.01 indicates high statistical significance. All reported *p* values were two-tailed. Statistical analyses involved Student's *t*-test and one-way ANOVA.

## 3. Results

### 3.1. Identifying Prognosis-Related SNHGs in Prostate Cancer

**Figure 1:**
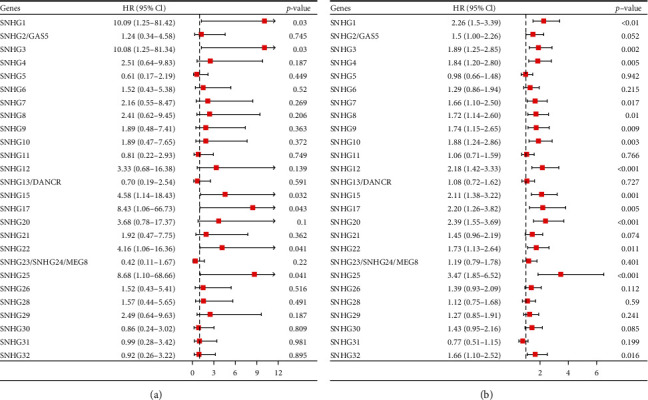
Analysis of the relationship between SNHG expression and OS/PFI of 499 patients with prostate cancer using one-way Cox regression, presented using forest plots.

To identify the prognosis-related SNHGs in prostate cancer, we first explored the expression data of the SNHG family (SNHG1, SNHG2/GAS5, SNHG3, SNHG4, SNHG5, SNHG6, SNHG7, SNHG8, SNHG9, SNHG10, SNHG11, SNHG12, SNHG13/DANCR, SNHG14, SNHG15, SNHG16, SNHG17, SNHG18, SNHG19, SNHG20, SNHG21, SNHG22, SNHG23/24/MEG8, SNHG25, SNHG26, SNGH27, SNHG28, SNHG29, SNHG30, SNHG31, and SNHG32) in the PRAD cohort from TCGA database. The prognostic significance of SNHGS was evaluated between two groups divided by the mean expression level of each SNHG mRNA. Univariate Cox regression analysis suggested that SNHG1, SNHG3, SNHG15, SNHG17, SNHG22, and SNHG25 have significant prognostic effects on both OS and PFI (Figures 1(a) and 1(b)). Kaplan–Meier analysis further confirmed the prognostic values of the six SNHGs in patients with PC (Supplementary Figures 1A–1L).

Associations between SNHG expression and OS (a) and PFI (b) of prostate cancer patients were analyzed by a one-way Cox regression test and visualized by forest plots.

### 3.2. Correlations between the Expression of SNHGs and the Clinical Characteristics of PC

**Figure 2:**
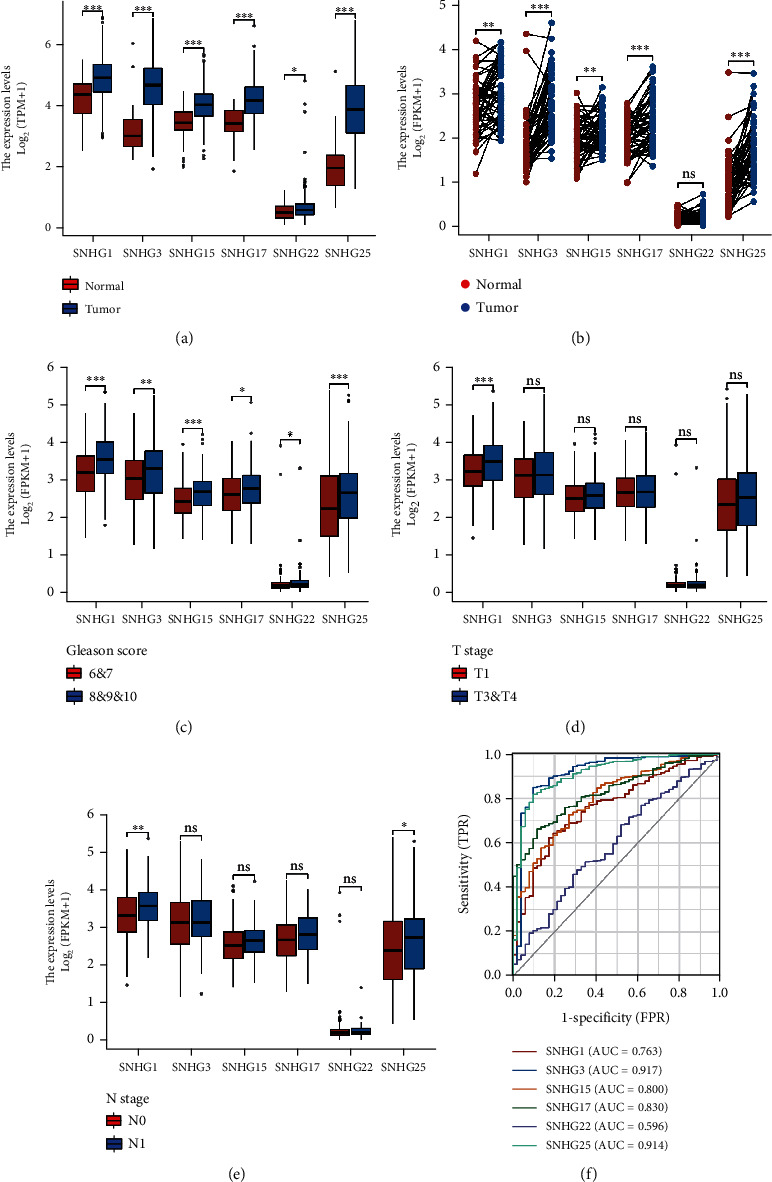
Relationship between six SNHGs and clinical and pathological characteristics of PC patients. (a) Box plots indicating the expression of six SNHGs in normal prostate tissue samples (*n* = 52) and prostate tumor tissue samples (*n* = 499). (b) Dot plots indicating the expression of six SNHG2 in 52 pairs of normal prostate samples and prostate tumor tissue samples (Wilcoxon signed rank test). (c) Box plots indicating the expression of six SNHGs in PC tumor tissues with low Gleason's score (6 & 7) (*n* = 293) and high Gleason's score (8 & 9 & 10) (*n* = 206). (d) Box plots indicating the expression of six SNHGs in PC tumor tissues with T2 stage (*n* = 189) and T3 & T4 stage (*n* = 303). (e) Box plots indicating the expression of six SNHGs in PC tumor tissues with (*n* = 347) or without (*n* = 79) lymph node metastasis. (f) ROC curve for six SNHGs in adjacent normal prostate samples and PC tumor samples. ns indicates not significant, ∗ indicates *p* < 0.05, ∗∗ indicates *p* < 0.01, and ∗∗∗ indicates *p* < 0.001, by Wilcoxon rank sum test or Wilcoxon signed rank test.

To evaluate the role of six candidate SNHGs in PC progression, we analyzed the correlation between the expression of the 6 SNHGs and the clinical outcomes of PC based on TCGA database. The characteristics of 499 patients with prostate cancer, including clinical and gene expression data, were collected from TCGA database (Table 1). The results suggested that the expression levels of the 6 SNHGs were significantly elevated in prostate tumor tissue samples compared with normal prostate tissue samples (Figure 2(a)). However, SNHG22 expression was not significant when compared between 52 pairs of prostate cancer tissue samples and adjacent normal tissues (Figure 2(b)). All six SNHGs were significantly upregulated in high Gleason's score tumors (Gleason's score = 8&9&10) compared with low Gleason's score tumors (Gleason's score = 6&7) (Figure 2(c)). However, there were no significant differences observed when comparing the expression of the six SNHGs between T2 stage tumors and T3 & T4 stage tumors (Figure 2(d)). Additionally, only SNHG1 and SNHG25 demonstrated significantly higher expression in N1 stage tumors than in N0 stage tumors (Figure 2(e)). We also used a receiver operating characteristic (ROC) curve to examine the prognostic value of the six SNHGs by comparing SNHG expression in normal prostate samples and PC samples. The area under the curve (AUC) values for SNHG1, SNHG15, and SNHG17 ranged from 0.7 to 0.9, and AUC values for SNHG3 and SNHG25 were above 0.9, indicating a high diagnostic potential (Figure 2(f)). Combined, the above data suggested that SNHG1, SNHG3, SNHG15, SNHG17, and SNHG25 have a much greater prognostic value in prostate cancer.

### 3.3. Identification of Prognosis-Related SNHGs by Lasso Regression Analysis and Construction of a Prognostic Model

**Figure 3:**
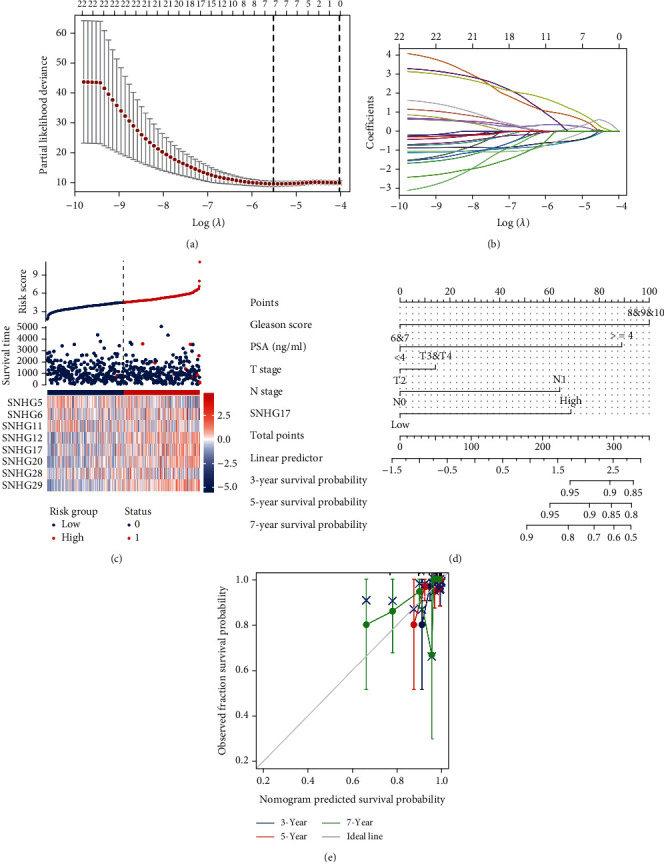
LASSO analysis further confirmed SNHG17 to be a potential prognostic marker for PC and prediction model construction. (a) A total of 33 SNHGs were further analyzed by LASSO analysis to explore potential risk factors for the OS of PC patients. (b) LASSO coefficient profiles. (c) The risk score, survival status, and heat map of eight SNHGs in patients with PC. (d) A nomogram for predicting the probability of 3-, 5-, and 7-year OS in PC patients. (e) Calibration plots validating the efficiency of nomograms for the OS of PC patients.

To further explore which SNHGs could be used to indicate the prognosis of PC, LASSO regression analysis was performed to evaluate the prognostic potential of all 32 SNHGs (Figures 3(a) and 3(b)). Eight SNHGs were selected for the model: SNHG5, SNHG6, SNHG11, SNHG12, SNHG17, SNHG20, SNHG28, and SNHG29 (Figure 3(c)). Since we identified five potential SNHGs by Cox regression univariate analysis, Kaplan–Meier analysis, and ROC curve analysis, SNHG17 was found to be the only SNHG confirmed by our comprehensive analysis.

Univariate Cox regression analysis showed that high SNHG17 expression was significantly correlated with poor OS in PC patients (HR = 8.428, 95%CI = 1.064–66.730, *p* = 0.043). Moreover, multivariate regression analysis further confirmed that SNHG17 expression was an independent prognostic factor for OS in PC patients (HR = 13.530, 95%CI = 1.053–173.563, *p* = 0.046) (Table 2). We then constructed a nomogram using T stage, N stage, PSA level, Gleason's score, and SNHG17 expression as indicators to predict 3-, 5-, and 7-year-old PC patients (Figure 3(d)). The calibration curve was also performed to present the prediction ability of the nomograms for the 3-, 5-, and 7-year clinical outcomes (Figure 3(e)). Collectively, the above data suggested that SNHG17 could be a prognostic indicator for patients with prostate cancer.

### 3.4. GO/KEGG and GSEA of SNHG17 in PC

**Figure 4:**
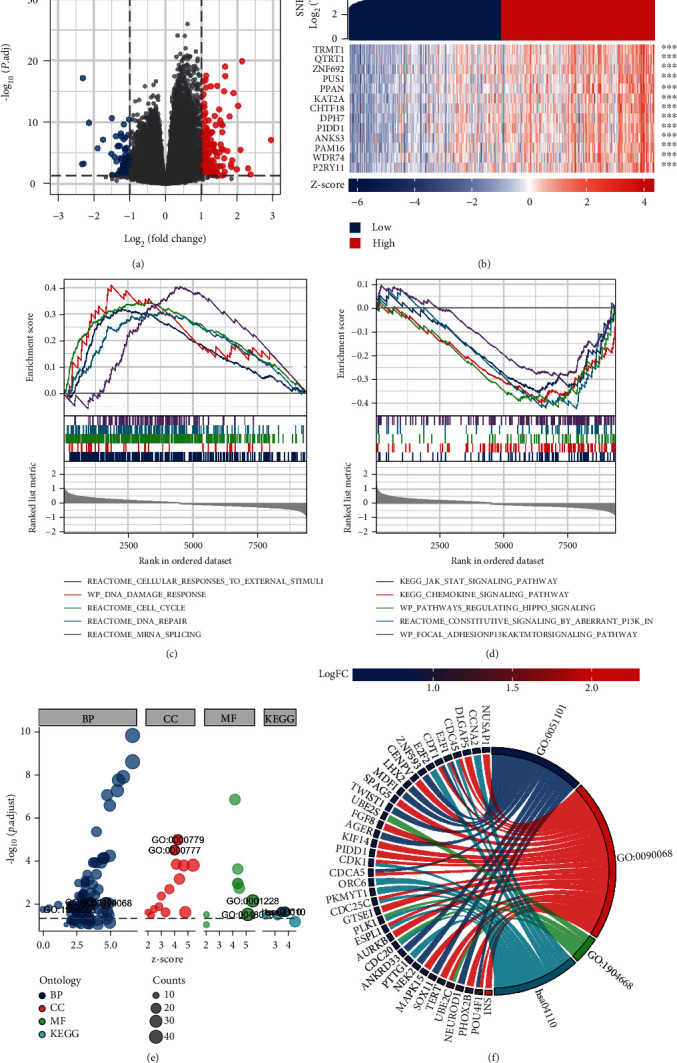
DEGs related to SNHG17 and enrichment analysis of DEGs in prostate cancer. (a, b) Volcano plots of the DEGs and heat map showing the top 13 DEGs. (c, d) Enrichment plots from the gene set enrichment analysis. (e) Plots from GO/KEGG analysis indicating the biological processes related to SNHG17 in prostate cancer. (f) Chord diagram indicating the enriched biological processes related to SNHG17 and significantly correlated DEGs to each biological process.

Next, we sought to investigate the putative mechanisms underlying the functions of SNHG17 in the progression of PC through GO, KEGG, and GSEA analyses. Data mining from TCGA database was used to identify the positive or negative coexpressed genes with SNHG17 in PC (Figures 4(a) and 4(b)). GO/KEGG and GSEA analyses were performed based on the most differentially coexpressed genes (logFC > 1 or logFC < −1, *p* < 0.05). As shown in Figures 4(c) and 4(d), GSEA suggested that SNHG17 was positively correlated with RNA metabolism, cellular response to external stimuli, DNA damage response, and cell cycle and negatively correlated with pathways in cancer and PI3K/AKT signaling in cancer, indicating that SNHG17 may not affect the progression of PC through canonical cancer-related signaling pathways.

GO and KEGG analyses were also conducted, and our indexes of interest are plotted in Figures 4(e) and 4(f). SNHG17 was found to be positively correlated with regulation of DNA binding (ID: GO:0051101, *Z* score = 3.61, *p*.adj = 0.008854), positive regulation of the cell cycle process (ID: GO:0090068, *Z* score = 4.69, *p*.adj = 0.00974114), positive regulation of ubiquitin protein ligase activity (ID: GO:1904668, *Z* score = 2, *p*.adj = 0.019254074), and the cell cycle (ID: hsa04110, *Z* score = 3.464101615, *p*.adj = 0.024914436) (Table 3). Taken together, the above results indicated that SNHG17 may participate in the progression of prostate cancer by modulating the cell cycle and the activity of ubiquitin-related proteins.

### 3.5. Correlation Analysis between SNHG17 Expression and the Infiltrating Level of Immune Cells in PC

**Figure 5:**
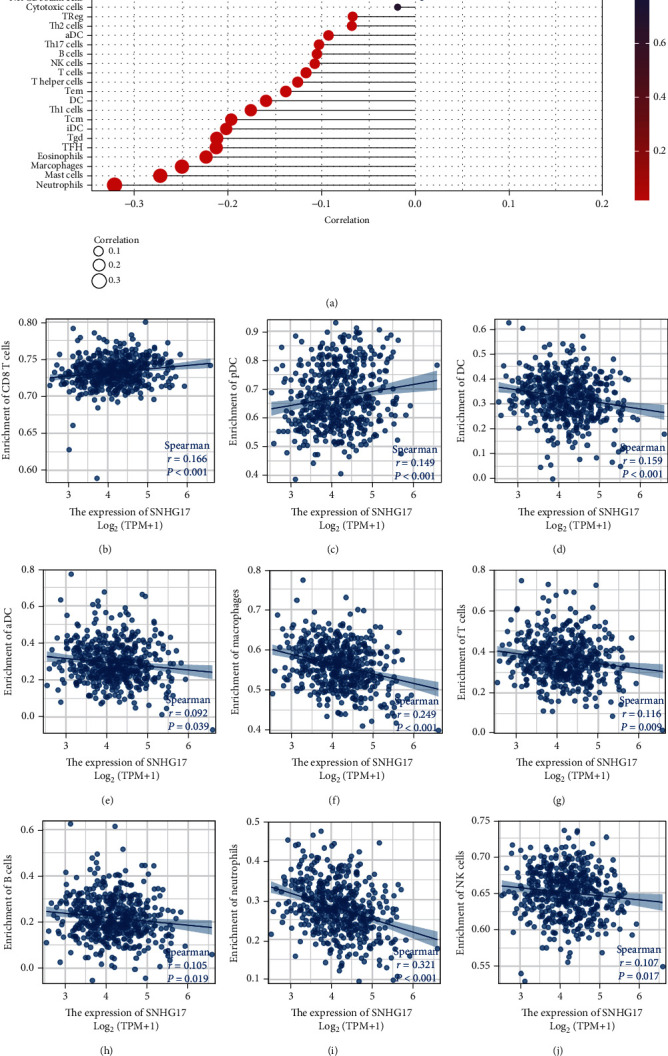
Relationship between SNHG17 and the infiltration level of immune cells in PC. (a) Correlation between SNHG17 and immune infiltration in PC by the ssGSEA method. (b–j) Correlation analysis between SNHG17 and the infiltration level of each immune cell was plotted separately by Spearman's correlation coefficient analysis.

We then analyzed the correlation between SNHG17 expression and infiltrating immune cells in prostate cancer based on the gene expression profile and immune cell infiltrating data from TCGA databases (Figure 5(a)). The results suggested that SNHG17 expression was negatively correlated with the infiltration level of most immune cells, such as dendritic cells (DCs) (Figure 5(d)), activated DCs (aDCs) (Figure 5(e)), macrophages (Figure 5(f)), T cells (Figure 5(g)), B cells (Figure 5(h)), neutrophils (Figure 5(i)), and NK cells (Figure 5(j)). In addition, the infiltration levels of CD8+ T cells (Figure 5(b)) and pDCs (Figure 5(c)) were found to be significantly associated with decreased SNNHG17 expression. The enrichment levels of immune cells between the SNHG-low group and the SNHG17-high group in prostate cancer were analyzed and are shown in Supplementary Figures 2A–2L.

### 3.6. Construction and Correlation Analysis of the ceRNA Network

**Figure 6:**
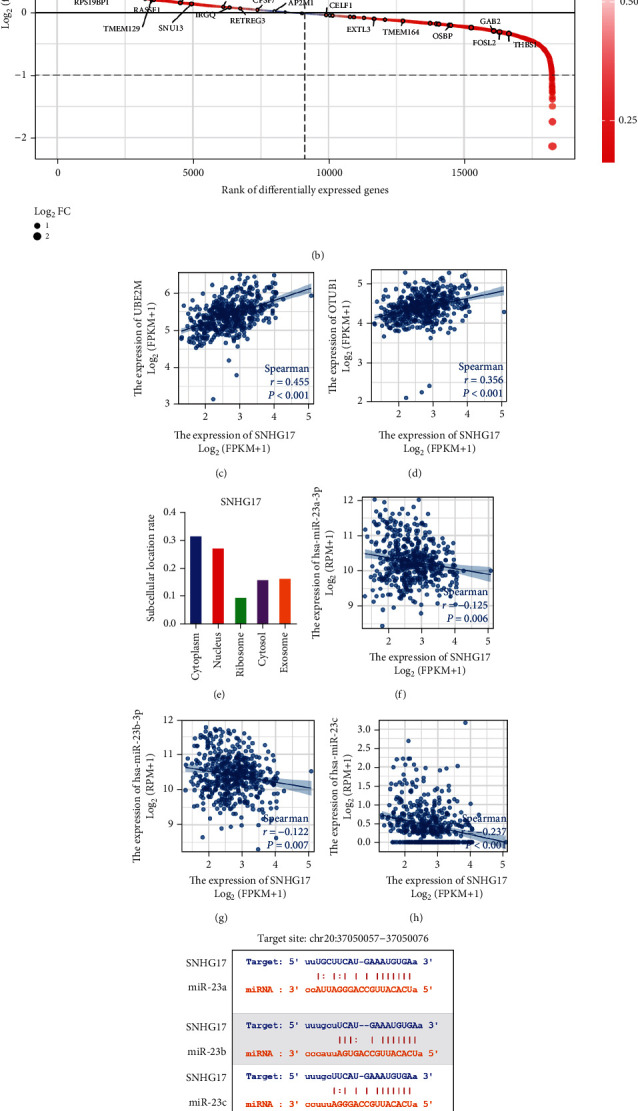
Construction of the ceRNA network. (a) Thirty-seven ceRNAs of SNHG17 were predicted by ENCORI. (b) Plot indicating the ranking of 37 ceRNAs in the DEGs related to SNHG17. (c, d) Correlation analysis between SNHG17 and UBE2M/OTUB1 by Spearman's correlation analysis. (e) Subcellular localization of SNHG17 analyzed by lncLocator. (f–h) Correlation analysis between SNHG17 and miR-23a-3p/23b-3p/23c by Spearman's correlation analysis. (i) Predicted binding sites of SNHG17 and miR-23a-3p/23b-3p/23c by ENCORI. (j) The triple regulatory network in PC. Red circle indicates lncRNA, blue square indicates mRNA, and yellow triangle indicates miRNAs.

Accumulating evidence supports that lncRNAs can function as ceRNAs to sponge target miRNAs or circRNAs and consequently release the expression of their downstream mRNAs. Therefore, we sought to determine the potential ceRNA networks underlying the function of SNHG17 in PC. We used the “ceRNA network: lncRNA-ceRNA” module of ENCORI tools [19] to search for the experimental supported ceRNA network of SNHG17, and the parameters were set as *p* ≤ 0.01, FDR < 0.01, and pancancer ≥ 6. The results indicated 37 protein-coding RNAs that may be regulated by SNHG17 via potential ceRNA networks (Figure 6(a)). The correlations between the expression of 37 candidate mRNAs and SNHG17 in prostate cancer were then plotted based on the differential gene expression profile (Figure 6(b)), and within the 37 candidates, we aimed to explore target genes that were positively correlated with SNHG17 and had the potential to drive cancer progression. Finally, we identified UBE2M and OTUB1 as ideal target genes. UBE2M and OTUB1 were found to be significantly correlated with SNHG17 (Figures 6(c) and 6(d)). UBE2M and OTUB1 are both cancer-related genes and have been reported to regulate the immune response and antitumor immunity. The subcellular location of SNHG17 was analyzed by lncLocator [21] (Figure 6(e)).

Next, we used the miRNA target module of ENCORI tools to explore the miRNAs between SNHG17 and UBE2M/OTUB1. Seventeen SNHG17-targeted miRNAs, 21 miRNAs that target UBE2M, and 13 miRNAs that target OTUB1 were identified, and miR-23a-3p/miR23b-3p/miR-23c were found to be the three common miRNAs that linked SNHG17 with UBE2M and OTUB1 (Figure 6(j)). The potential binding sites are shown in Figure 6(h). We also analyzed the expression correlations between SNHG17 and miR23a-3p and between miR-23b-3p and miR-23c, and the results suggested that SNGH17 was negatively correlated with the three miRNAs (Figures 6(f)–6(i)).

### 3.7. Clinical Implications of miR-23a-3p/23b-3p/23c in PC

To further identify the biological functions of miR-23a-3p/23b-3p/23c in prostate cancer, we analyzed their expression profiles based on TCGA databases. The expression of all three miRNAs was significantly decreased in prostate tumor tissues compared with normal prostate samples (Supplementary Figures 3A–3C). Kaplan–Meier analysis showed that high expression of miR-23b-3p and miR-23c was significantly correlated with better PFI in PC patients (Supplementary Figures 3D–3F). Taken together, miR-23a-3p/23b-3p/23c were very likely to play antitumor roles in prostate cancer.

Targets of miRNAs were analyzed using the miRNA-target function of ENCORI tools. A total of 1046 target genes of miR-23a-3p, 978 target genes of miR-23b-3p, and 76 target genes of miR-23c were recognized and are shown in a Venn diagram (Supplementary Figure 3G). The potential mechanisms underlying the functions of the three miRNAs were explored by GO/KEGG analysis. As shown in Supplementary Figure 3H, miR-23a-3p/23b-3p/23c were found to be significantly correlated with the regulation of autophagy, protein monoubiquitination, histone methylation, mTOR signaling, and p53 signaling.

### 3.8. Assessment of SNHG17 and OTUB1 Expression in a Cohort of 52 PC Patients

**Figure 7:**
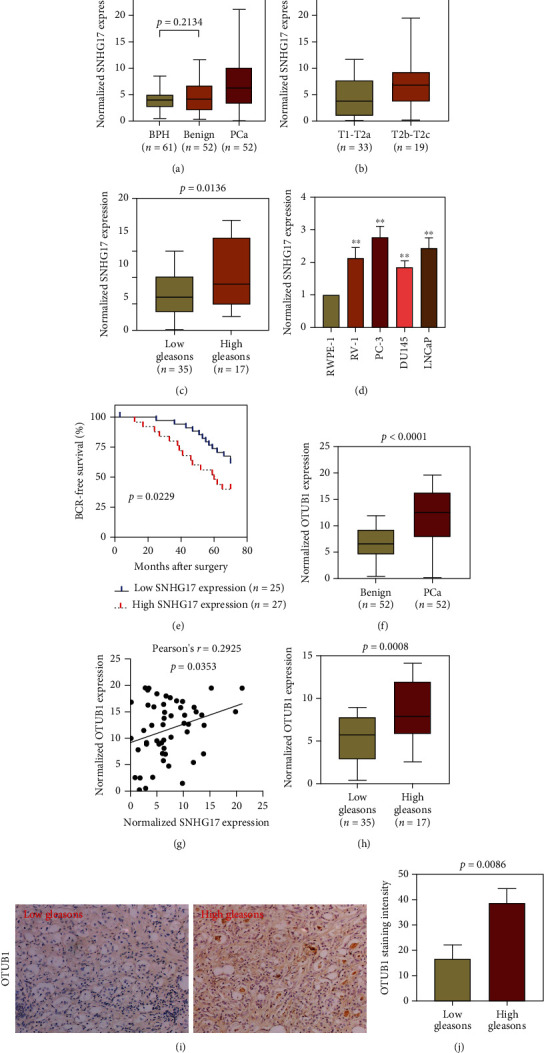
SNHG17 is overexpressed in PC tumors and correlates with tumor progression. (a) Analysis of SNHG17 expression in 61 BPH tissues and 52 pairs of benign prostate tissues and prostate cancer tissues by qRT–PCR (one-way ANOVA). (b) SNHG17 expression between PC tumor tissues with T1-T2a stage and T2b-T2c stage. (c) SNHG17 expression between PC tumor tissues with low Gleason scores and high Gleason scores. (d) SNHG17 expression in one normal prostate epithelial cell line and four prostate cancer cell lines (one-way ANOVA). (e) High SNHG17 expression positively correlated with poor BCR-free survival by Kaplan–Meier plotting (Cox regression analysis). (f) OTUB1 expression between 52 pairs of benign prostate tissues and prostate cancer tissues by qRT–PCR. (g) Correlation between SNHG17 and OTUB1 expression in 52 PC tumor tissues by Pearson's correlation coefficient analysis. (h) OTUB1 expression between PC tumor tissues with low Gleason's score (≤7, *n* = 35) and those with high Gleason's score (>7, *n* = 17) by qRT–PCR. (i) Representative images of IHC staining of OTUB1 in PC tumor tissues with low or high Gleason's score. Magnification = 200x. (j) The intensity of OTUB1 staining in PC tumor tissues with low (*n* = 3) or high Gleason's scores (*n* = 3) was analyzed and compared. ∗∗ indicates *p* < 0.01, analyzed by Student's *t*-test or one-way ANOVA.

To further demonstrate the prognostic value of SNHG17 in PC, we examined SNHG17 expression by qRT–PCR in a cohort of 52 PC patients who were hospitalized in our department. The results showed that SNHG17 was upregulated in PC tumor specimens compared with benign PC tissues and adjacent normal PC tissues (Figure 7(a)). In addition, SNHG17 expression was found to be elevated in PC tumor tissues with advanced tumor stage (Figure 7(b)) and high Gleason's score (Figure 7(c)). We also examined SNHG17 expression in one normal prostate epithelial cell line (RWPE-1) and four PC cancer cell lines (RV-1, PC-3, DU145, and LNCaP), and we found that SNHG17 expression was significantly upregulated in PC cancer cell lines (Figure 7(d)). The clinical outcomes, such as overall survival, metastasis, and biochemical recurrence status, of 52 patients were followed up, and information was analyzed with SNHG17 expression (Table 4). The results suggested that a high level of SNHG17 was remarkably correlated with poor BCR-free survival (Figure 7(e)).

OTUB1 expression was analyzed in 52 pairs of adjacent normal prostate tissue and PC tumor tissues by qRT–PCR, and the results suggested that OTUB1 was transcriptionally upregulated in PC tumor tissues (Figure 7(f)). Pearson's correlation analysis showed a positive correlation between the expression of SNHG17 and OTUB1 in 52 PC tumor specimens (Figure 7(g)). To verify the correlation between OTUB1 expression and the clinical progression of prostate cancer, PC patients were divided by Gleason's score, and the mRNA level of OTUB1 was analyzed by qRT–PCR. The results suggested that OTUB1 was overexpressed in the high Gleason score group (Gleason score > 7, *n* = 17) compared with the low Gleason score group (Figure 7(h)). To further confirm the result, we detected OTUB1 expression in 6 PC tumor tissue specimens by immunochemistry staining, among which three tissues were pathologically diagnosed as Gleason's score ≤ 7 and the other three were diagnosed as Gleason's score > 7. The results demonstrated that OTUB1 staining was stronger in the high Gleason's score group than in the low Gleason's score group (Figures 7(i) and 7(j)).

### 3.9. Validation of SNHG17/miR-23a/OTUB1 Axis in Prostate Cancer

**Figure 8:**
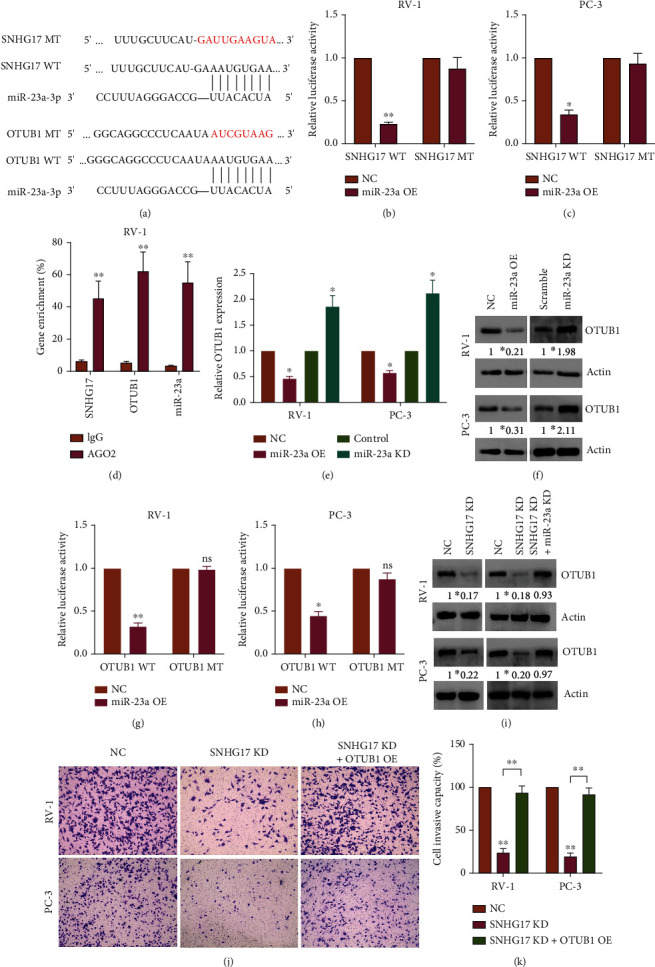
Validation of the SNHG17/miR-23a-3p/OTUB1 axis in prostate cancer. (a) The binding sites and sequences between SNHG17 and miR-23a-3p and miR-23a-3p and OTUB1. A dual luciferase reporter assay was performed to evaluate the interactions between luciferase reporter vectors containing wild-type SNHG17 or mutant SNHG17 in (b) RV-1 and (c) PC-3 cells. (d) RIP assay was performed to assess the enrichment of SNHG17, miR-23a, and OTUB1 in IgG or AGO2 pulled-down RNA products. (e) qRT–PCR analysis indicated that overexpression of miR-23a-3p decreased the mRNA expression of OTUB1, while knockdown of miR-23a-3p prompted the expression of OTUB1 in PC cells. (f) Western blot revealed a similar result that miR-23a-3p modulated OTUB1 protein expression. A dual luciferase reporter assay was performed to evaluate the interaction between miR-23a-3p and the 3′UTR of the OTUB1 gene. A luciferase reporter vector containing wild-type OTUB1 or mutant OTUB1 was cotransfected with negative control or miR-23a-3p mimics into (g) RV-1 and (h) PC-3 cells. (i) Western blot analysis suggested that knockdown of SNHG17 significantly decreased OTUB1 expression, while dual knockdown of SNHG17 and miR-23a-3p restored OTUB1 expression. (j, k) Transwell assays showed that knockdown of SNHG17 inhibited cell invasion, and reexpression of OTUB1 in SNHG17-knockdown cells rescued the cell invasive capacity (one-way ANOVA). Invaded cells were captured in three random visual fields at a magnification of 200x. ∗ indicates *p* < 0.05 and ∗∗ indicates *p* < 0.01, analyzed by Student's *t*-test or one-way ANOVA.

As we analyzed the ceRNA network through which SNHG17 potentially mediated the progression of prostate cancer, we then sought to validate our hypothesis using PC cell lines. The binding sites between SNHG17 and miR-23a and between miR-23a and OTUB1 were analyzed by ENCORI as described previously (Figure 8(a)). Luciferase reporter vectors encoding the wild-type SNHG17 gene (SNHG17 WT) and binding site-mutated SNHG17 (SNHG17 MT) were transfected into PC cells with control or miR-23a-3a mimics, and luciferase activity was then measured 24 hours posttransfection. The results showed that miR-23a overexpression significantly inhibited the transcription of luciferase reporter vectors containing wild-type SNHG17; however, mutation of SNHG17 diminished the inhibitory effects (Figures 8(b) and 8(c)). To verify the interactions between SNHG17, miR-23a, and the OTUB1 gene, a RIP assay was subsequently performed on RV-1 cells. The results showed that the levels of SNHG17, miR-23a, and OTUB1 genes were significantly enriched in the AGO2 pulled-down RNA products (Figure 8(d)). miR-23a-3p was overexpressed or knocked down in PC cells by transfecting the miR-23a mimics or inhibitors into the cells (Supplementary Figure 4A), and mRNA and protein expression of OTUB1 was detected by qRT–PCR and western blot assay (Figures 8(e) and 8(f)). A dual luciferase assay was performed to verify the interaction between miR-23a-3p and the OTUB1 gene using luciferase reporter vectors containing wild-type OTUB1 (OTUB1) or miR-23a binding site mutated OTUB1 (OTUB1 MT) (Figures 8(g) and 8(h)). The results confirmed that miR-23a-3p regulated OTUB1 expression by directly interacting with the OTUB1 gene.

Small interfering RNAs (siRNA) against SNHG17 or scramble RNA oligo were separately transfected into PC cells (Supplementary Figure 4B), and OTUB1 protein expression was measured by western blot. The results indicated that SNHG17 knockdown inhibited OTUB1 expression, and knockdown of both SNHG17 and miR-23a-3p rescued OTUB1 expression, which was hampered by SNHG17 knockdown alone (Figure 8(i)). Finally, PC cells were treated with SNHG17 knockdown alone or a combination of SNHG17 knockdown and OTUB1 overexpression (Supplementary Figure 4C), and the invasive capacity of PC cells was assessed by transwell assay. The results demonstrated that SNHG17 knockdown decreased the number of invaded cells, and OTUB1 overexpression rescued the hampered cell invasive ability (Figures 8(j) and 8(k)), indicating that SNHG17 modulated PC cell invasive capacity by regulating OTUB1.

### 3.10. Clinical Implication and Enrichment Analysis of UBE2M/OTUB1 in PC

Expression profiling analysis suggested that the expression of UBE2M and OTUB1 was significantly increased in prostate tumor tissue samples compared with normal tissues (Supplementary Figure 5A). Meanwhile, UBE2M was found to be overexpressed in tumor tissues with a high Gleason score (Gleason's score = 8, 9, 10) compared with tumor tissues with a low Gleason score (Gleason's score = 6&7) (Supplementary Figure 5B). The expression of OTUB1 was notably elevated in tumor tissues with lymph node metastasis (Supplementary Figure 5C). The expression of UBE2M/OTUB1 in prostate cancer based on lymph node metastasis status, TP53 mutation status, and promoter methylation level of UBE2M/OTUB1 was analyzed using UALCAN software [18]. The expression of UBE2M/OTUB1 was significantly associated with TP53 mutation and nodal metastasis of prostate cancer (Supplementary Figures 6A–6D). Moreover, altered promoter methylation levels were observed in both UBE2M and OTUB1 (Supplementary Figures 6E and 6F).

GO/KEGG and GSEA analyses were performed to examine the underlying mechanisms of UBE2M and OTUB1 in prostate cancer. As shown in Supplementary Figures 5D and 5E, UBE2M and OTUB1 shared some similar enriched biological processes, such as regulation of RUNX3 expression and activity, stabilization of p53, and response to metal ions. On the other hand, UBE2M and OTUB1 were negatively enriched in some cancer-related or immune response-related pathways, such as PI3K/AKT signaling in cancer, the JAK/STAT signaling pathway, pathways in cancer, and the chemokine signaling pathway (Supplementary Figures 5D and 5E). GO/KEGG analysis suggested that UBE2M and OTUB1 were closely correlated with proteasome, ubiquitin-like protein binding, ribosome, and NIK/NF-kappa B signaling (Supplementary Figures 5F and 5G).

### 3.11. Correlation Analysis of Immune Infiltration and UBE2M/OTUB1 in PC

**Figure 9:**
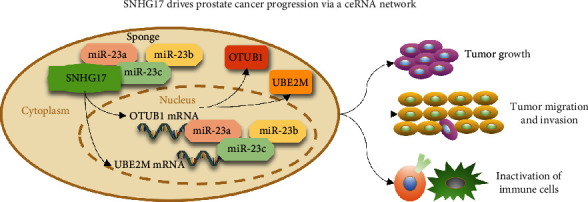
Diagram indicating the SNHG17-related ceRNA network in PC. SNHG17 competitively interacts with miR-23a-3p/23b-3p/23c and releases the expression of UBE2M and OTUB1, thereby promoting the tumorigenesis of prostate cancer and inhibiting the activation and infiltration of immune cells in the tumor microenvironment.

Since we have identified the immune-infiltration correlated signature of SNHG17, we next sought to determine whether the potential downstream targets were also associated with the infiltrating level of immune cells in prostate cancer. Immune cell infiltration with the expression of UBE2M and OTUB1 was analyzed using the ssGSEA algorithm based on TCGA databases and TIMER tools [16]. The results showed that the expression of UBE2M was significantly correlated with decreased infiltrating levels of most immune cell types except for NK CD56 bright cells and plasmacytoid DCs (pDCs) (Supplementary Figure 7A). Similarly, OTUB1 expression was negatively associated with the infiltration level of most immune cell types except for IK CD56 bright cells and NK CD56 bright cells (Supplementary Figure 7B). The results from TIMER tools showed that UBE2M was negatively correlated with the infiltration of B cells, CD8+ T cells, CD4+ T cells, macrophages, neutrophils, and DCs (Supplementary Figure 7C), while OTUB1 was found to be significantly correlated with the infiltration of macrophages only (Supplementary Figure 7D). We also analyzed the association between immune cell infiltration levels and somatic copy number alterations for UBE2M or OTUB1 by the SCNA module. The results suggested that deep deletion of OTUB1 significantly decreased the infiltrating level of CD4+ T cells, neutrophils, and DCs (Supplementary Figure 8A), while deep deletion of UBE2M was closely related to a decreased infiltrating level of macrophages (Supplementary Figure 8B). Taken together, the above results indicated that the expression of UBE2M and OTUB1 was related to decreased immune infiltration levels in prostate cancer. The underlying mechanisms of SHNG17 and the related ceRNA network are briefly shown in Figure 9.

## 4. Discussion

Accumulating studies have uncovered the functions and roles of SNHGs in the initiation and progression of cancer. SNHGs have been identified as critical tumor inducers in many types of human cancers, including hepatocellular carcinoma [22], colorectal cancer [23], ovarian cancer [24], esophageal cancer [25], bladder cancer [26], renal clear cell cancer [27], and prostate cancer [28]. Some SNHGs have been recognized as important cancer predictors and biomarkers, such as SNHG1 [29] and SNHG12 [30]. The biological functions of lncRNAs differ with their cellular locations, and the molecular mechanisms of SNHGs can vary, as most of them are located both in the nucleus and cytoplasm [5]. In the nucleus, SNHGs modulate methylation enzymes and influence DNA methylation or interact with transcription factors and influence gene transcription [31]. In the cytoplasm, SNHGs act as miRNA sponges and release the target genes of miRNAs or prevent protein ubiquitination through RNA–protein binding [31]. Herein, we explored the prognostic values of SNHG17 in PC and identified SNHG17 as an important predictive molecule by Cox regression univariate analysis, Kaplan–Meier analysis, and LASSO regression analysis. Moreover, enrichment analysis based on the expression profile of SNHG17 in PC suggested that it potentially participates in the progression of PC by modulating RNA splicing, protein ubiquitination, cell cycle arrest, and the cellular response to extracellular stimuli. Since SNHG17 is located both in the nucleus and cytoplasm, we built a novel ceRNA regulatory network that linked SNHG17 with two ubiquitination-related proteins, UBE2M and OTUB1. We also noticed that SNHG17 potentially interacts with miR-23a-3p, miR-23b-3p, and miR-23c, thereby suppressing their molecular functions and releasing the expression of their downstream target genes. SNHG17 expression in PC tumors and its prognostic signature were further examined in our cohort of 52 PC patients, and the SNHG17/miR-23a-3p/OTUB1 axis was validated in PC cell lines by luciferase assay and RIP assay. Our data also revealed that SNHG17 promoted cell invasive capacity by modulating OTUB1 expression. The underlying mechanisms of UBE2M, OTUB1, and miR-23a/23b/23c were analyzed by GO/KEGG and GSEA. Additionally, we observed that the expression of SNHG17, UBE2M, and OTUB1 was significantly correlated with a decreased infiltrating level of immune cells, indicating the immunological role of SNHG17 and its ceRNA regulatory network in prostate cancer.

It has been widely recognized that SNGH17 functions as an oncogene in many cancers, such as non-small-cell lung cancer (NSCLC) [32], breast cancer (BC) [33], gastric cancer (GC) [23], and renal cell carcinoma (RCC) [34]. The tumorigenesis function and related mechanisms of SNHG17 in driving prostate cancer have also been studied. Wu el al. found that knockdown of SNGH17 weakened the proliferation, invasion, and migration of prostate cancer cells. Mechanistically, SNHG17 and its homolog SNORA71B were transactivated by signal transducer and activator of transcription 5A (STAT5A), and SNHG17 sponged with miR-339-5p and released the expression of STAT5A, thereby forming a regulatory loop and exacerbating prostate cancer progression [35]. Bai et al. reported that SNHG17 promoted the progression of castration-resistant prostate cancer via the miR-144/CD51 axis, indicating that SNHG17 may serve as a therapeutic target in prostate cancer [36]. In the present study, we have concluded some similar results as literatures previously reported, that expression of SNHG17 was found to be significantly correlated with the progression of prostate cancer, and demonstrated great prognostic values for patients with PC. We also constructed a nomogram based on SNHG17 expression and other clinical features of PC patients for outcome prediction. Additionally, we explored novel mechanisms that may further uncover the carcinogenetic functions of SNHG17 in prostate cancer. SNHG17 was found to be closely related to the DNA damage response, cell cycle, and cellular response to external stimuli, suggesting that SNHG17 may promote the cell cycle and enhance cellular resistance to external stimuli and pressures.

miRNAs are a class of small noncoding RNAs that have been recognized as hallmarks of cancer development and progression; furthermore, some miRNAs have been proven to be promising prognostic biomarkers in cancers. Using bioinformatic tools, we discovered that miR-23a-3p, miR-23b-3p, and miR-23c were targeted by SNHG17, and the interactions were experimentally supported. miR-23a-3p and miR-23b-3p belong to the miR-23/24/27 cluster and share some similar biological functions and target genes. More importantly, they have been demonstrated as prognostic biomarkers in prostate cancer and were proven to influence the malignant behavior of PC cells. Strand et al. identified a four-miRNA prognostic biomarker panel for the prediction of long-term prostate cancer, and they found that inhibition of miR-23a-3p significantly reduced survival of PC3 and DU145 cells, indicating the oncogenic role of miR-23a-3p in PC [37]. In contrast, Cai et al. reported that downregulation of miR-23a suppresses prostate cancer metastasis by targeting the PAK9-LIMK signaling pathway, suggesting the antitumor function of miR-23a in PC [38]. Most studies have recognized miR-23b-3p as a tumor suppressor in prostate cancer. Jiang et al. reported that CRISPR/Cas9-mediated miR-23b knockout significantly increased the proliferation, invasion, and metabolism of LNCaP cells [39]. Majid et al. found that miR-23b functions as a tumor suppressor with diagnostic and prognostic signatures in prostate cancer and represses the expression of the proto-oncogene Src kinase [40]. The miR-23b/27b cluster was also demonstrated to suppress prostate cancer metastasis via Huntingtin-interacting protein 1 [41]. There are few reports implicating the role of miR-23c in prostate cancer. Martínez-González suggested that miR-23c can be an aggressiveness biomarker for PC and may drive the expression of MAPK1 and FGFR3 [42]. In the present study, we showed that the expression of miR-23a-3p/23b-3p/23c was notably decreased in prostate cancer tumor tissue samples and was negatively correlated with SNHG17 expression. GO/KEGG analysis suggested that they may induce the progression of PC by modulating biological processes such as autophagy, histone methylation, protein monoubiquitination, and mTOR signaling.

In the current study, OTUB1 and UBE2M were recognized as downstream targets of miR-23a-3p/23b-3p/23c. OTUB1 is overexpressed in human cancers and acts to suppress ferroptosis of prostate cancer by promoting SLC7A11 stability [43]. Although OTUB1 belongs to the OTU family of deubiquitinases, emerging evidence has revealed that OTUB1 stabilizes its substrate protein by blocking the E2-conjugating enzymes that are essential for their polyubiquitination rather than functioning as a deubiquitinase [44, 45]. OTUB1 has also been demonstrated to stabilize several oncoproteins, including cIAP [46], FOXM1 [47], and Mdmx [48]. Moreover, OTUB1 was recently reported to promote cancer cell immunosuppression by preventing ER-associated degradation of the immune checkpoint protein PD-L1 [49]. Interestingly, UBE2M belongs to the E2-conjugating enzyme family; therefore, it possesses completely different or even opposite functions than OTUB1. However, similar to OTUB1, UBE2M is widely reported to play an oncogenic role in cancers. In prostate cancer, UBE2M interacts with NRPL2 and stabilizes its protein, and depletion of NRPL2 or UBE2M significantly increases the niraparib sensitivity of CRPC cells and enhances niraparib-induced tumor growth inhibition [50]. In hepatocellular cancer, UBE2M negatively regulates p53 by binding to MDM2 and ribosomal protein L11 [51] and promotes cellular proliferation via *β*-catenin/cyclin D1 signaling [52].

GSEA suggested that OUTB1 and UBE2M were both enriched in biological processes such as stabilization of p53, regulation of RUNX3 expression and activity, and response to metal ions. We also noticed that both genes were negatively related to JAK-STAT signaling, chemokine signaling, and type II interferon signaling, which are pathways closely associated with the immune response and antitumor immunity. OTUB1 has been identified to regulate NK/CD8+ T cell activation, autoimmune diseases, PD-L1-mediated immune evasion, and viral or bacterial infection-related immune responses [53, 54]. Furthermore, its role in tumorigenesis and antitumor immunity has drawn great attention [53, 55]. UBE2M functions as an E2 NEDD8-conjugating enzyme and plays a critical role in the process of protein neddylation, which has been proven to be associated with antitumor immunity and tumor microenvironment modification [56–58]. Taken together, SNHG17 is very likely to influence immune infiltration by upregulating the expression of OTUB1 and UBE2M in prostate cancer.

However, there are some limitations in our study. First, our analysis is mainly based on bioinformatic analysis and data mining. The prognostic value, biological function, and regulatory network of SNHG17 in prostate cancer still need to be further validated by experiments, although we have done some work to verify our hypothesis. Second, although we included a cohort of 52 patients in this study, the size of our cohort is still not large enough. In addition, due to the short-term follow-up of our cohort, the result of analysis based on the expression of SNHG17 and overall survival of the patients was not significant. We need to enlarge the size of our cohort and continue with the follow-up to better screen the survival of the patients.

To conclude, we herein demonstrated that SNHG17 has great prognostic value in predicting the outcomes of PC patients and is closely associated with the infiltrating level of immune cells. SNHG17 potentially interacts with miR-23a-3p, 23b-3p, and miR-23c, thereby overexpressing their target genes OTUB1 and UBE2M. Through this ceRNA network, SNHG17 may promote the progression of prostate cancer and modulate the microenvironment of PC tumors, and targeting SNHG17 may suppress the malignancy of prostate cancer, activate antitumor immunity, and enhance the therapeutic effects of immune checkpoint blockade.

## Figures and Tables

**Table 1:** The clinicopathological characteristics and SNHG17 expression of 499 patients with prostate cancer.

Characteristic	Low expression of SNHG17	High expression of SNHG17	*p*
*n*	249	250	
T stage, *n* (%)			0.647
T2	96 (19.5%)	93 (18.9%)	
T3	145 (29.5%)	147 (29.9%)	
T4	4 (0.8%)	7 (1.4%)	
N stage, *n* (%)			0.175
N0	177 (41.5%)	170 (39.9%)	
N1	33 (7.7%)	46 (10.8%)	
M stage, *n* (%)			1.000
M0	225 (49.1%)	230 (50.2%)	
M1	1 (0.2%)	2 (0.4%)	
PSA (ng/ml), *n* (%)			1.000
<4	213 (48.2%)	202 (45.7%)	
≥4	14 (3.2%)	13 (2.9%)	
Gleason score, *n* (%)			0.259
6	24 (4.8%)	22 (4.4%)	
7	134 (26.9%)	113 (22.6%)	
8	28 (5.6%)	36 (7.2%)	
9	62 (12.4%)	76 (15.2%)	
10	1 (0.2%)	3 (0.6%)	
Age, *n* (%)			0.164
≤60	120 (24%)	104 (20.8%)	
>60	129 (25.9%)	146 (29.3%)	
Age, median (IQR)	61 (55, 66)	62 (57, 66)	0.226

**Table 2:** Univariate and multivariate Cox regression analyses of clinicopathological characteristics along with SNHG17 expression in PC patients.

Characteristics	Total (*N*)	Univariate analysis		Multivariate analysis	
		Hazard ratio (95% CI)	*p* value	Hazard ratio (95% CI)	*p* value
T stage	492				
T2	189	Reference			
T3&T4	303	3.294 (0.612-17.727)	0.165		
N stage	426				
N0	347	Reference			
N1	79	3.516 (0.778-15.896)	0.102		
M stage	458				
M0	455	Reference			
M1	3	59.383 (6.520-540.817)	**<0.001**	16.693 (1.705-163.463)	**0.016**
PSA (ng/ml)	442				
<4	415	Reference			
≥4	27	10.479 (2.471-44.437)	**0.001**	4.325 (0.955-19.585)	0.057
Gleason score	499				
6&7	293	Reference			
8&9&10	206	6.664 (1.373-32.340)	**0.019**	8.387 (0.831-84.642)	0.071
SNHG17	499				
Low	249	Reference			
High	250	8.428 (1.064-66.730)	**0.043**	13.520 (1.053-173.563)	**0.046**

**Table 3:** Enriched biological terms and pathways of SNHG17 in prostate cancer.

Ontology	ID	Description	Gene ratio	BgRatio	*p* value	*p*.adjust	*q* value
BP	GO:0051101	Regulation of DNA binding	13/560	124/18670	9.07*e* − 05	0.009	0.008
BP	GO:0090068	Positive regulation of cell cycle process	22/560	298/18670	1.02*e* − 04	0.010	0.009
BP	GO:1904668	Positive regulation of ubiquitin protein ligase activity	4/560	12/18670	3.27*e* − 04	0.019	0.018
CC	GO:0000779	Condensed chromosome, centromeric region	18/612	118/19717	2.43*e* − 08	1.13*e* − 05	1.07*e* − 05
CC	GO:0000777	Condensed chromosome kinetochore	16/612	105/19717	1.48*e* − 07	3.43*e* − 05	3.26*e* − 05
MF	GO:0001228	DNA-binding transcription activator activity, RNA polymerase II-specific	30/561	439/17697	7.13*e* − 05	0.008	0.007
MF	GO:0048018	Receptor ligand activity	30/561	482/17697	3.62*e* − 04	0.033	0.031
KEGG	hsa03010	Ribosome	14/229	158/8076	1.50*e* − 04	0.025	0.024
KEGG	hsa04110	Cell cycle	12/229	124/8076	1.95*e* − 04	0.025	0.024

**Table 4:** Associations between SNGH17 and clinicopathological characteristics of 52 PC patients.

Characteristics	*N*	SNHG17 expression		*p*
		Low	High	
Total cases	52	26	26	
Age (year)				
≤70	32	10	22	Not significant
>70	20	7	13	
Gleason score				
≤7	35	19	16	0.2453
>7	17	6	11	
Serum PSA (ng/*μ*l)				
≤20	19	6	13	0.4389
>20	33	14	19	
Tumor stage				
I-IIa	33	18	15	0.0484^∗^
IIb/IIc	19	5	14	
Bone metastasis				
Yes	29	13	16	0.0744
No	23	16	7	
Biochemical recurrence				
Yes	19	4	15	0.0031^∗∗^
No	33	21	12	
Overall survival				
Alive	44	26	18	0.258
Death	8	3	5	

## Data Availability

The datasets generated and analyzed during the current study are available from the corresponding author on reasonable request.

## Abbreviations

SNHGs: Small nucleolar RNA host genes
LncRNA: Long noncoding RNA
PC: Prostate cancer
CRPC: Castration-resistant prostate cancer
ceRNA: Competitive endogenous RNA
UBE2M: Ubiquitin-conjugating enzyme E2 M
OTUB1: Ovarian tumor deubiquitinase, ubiquitin aldehyde binding 1
TCGA: The Cancer Genome Atlas
LASSO: Least absolute shrinkage and selection operator
GO: Gene Ontology
KEGG: Kyoto Encyclopedia Genes and Genomes
GSEA: Gene set enrichment analysis.

## Supplementary Materials

Supplementary Materials Supplementary Figure 1: KM plotters indicating the relationship between SNHGs and OS/PFI of PC patients. Supplementary Figure 2: relationship between immune infiltration and expression of SNHG17 in prostate cancer. Supplementary Figure 3: implication of potential miRNAs in PC progression and GO/KEGG analysis of miRNAs. Supplementary Figure 4: validation of transfection efficiency. Supplementary Figure 5: correlation between UBE2M/OTUB1 expression and pathological features of PC and the related biological processes and pathways. Supplementary Figure 6: relationship between UBE2M/OTUB1 expression and lymph node metastasis and TP53 mutation status, and analysis of promoter methylation of UBE2M/OTUB1, in prostate cancer. Supplementary Figure 7: correlation between UBE2M/OTUB1 and infiltration level of immune cells in PC. Supplementary Figure 8: relationship between somatic mutation of UBE2M/OTUB1 and immune infiltration in prostate cancer.
