# Supplementary material for: Integrative Analysis and Experimental Validation Indicated That SNHG17 Is a Prognostic Marker in Prostate Cancer and a Modulator of the Tumor Microenvironment via a Competitive Endogenous RNA Regulatory Network
Source: Oxid Med Cell Longev. 2022 Jul 12;2022:1747604. doi: 10.1155/2022/1747604 (PMC9296331; doi:10.1155/2022/1747604)
Supplement: Supplementary Materials — Supplementary Figure 1: KM plotters indicating the relationship between SNHGs and OS/PFI of PC patients. Supplementary Figure 2: relationship between immune infiltration and expression of SNHG17 in prostate cancer. Supplementary Figure 3: implication of potential miRNAs in PC progression and GO/KEGG analysis of miRNAs. Supplementary Figure 4: validation of transfection efficiency. Supplementary Figure 5: correlation between UBE2M/OTUB1 expression and pathological features of PC and the related biological processes and pathways. Supplementary Figure 6: relationship between UBE2M/OTUB1 expression and lymph node metastasis and TP53 mutation status, and analysis of promoter methylation of UBE2M/OTUB1, in prostate cancer. Supplementary Figure 7: correlation between UBE2M/OTUB1 and infiltration level of immune cells in PC. Supplementary Figure 8: relationship between somatic mutation of UBE2M/OTUB1 and immune infiltration in prostate cancer. [file 1747604.f1.docx]

**Supplementary materials**


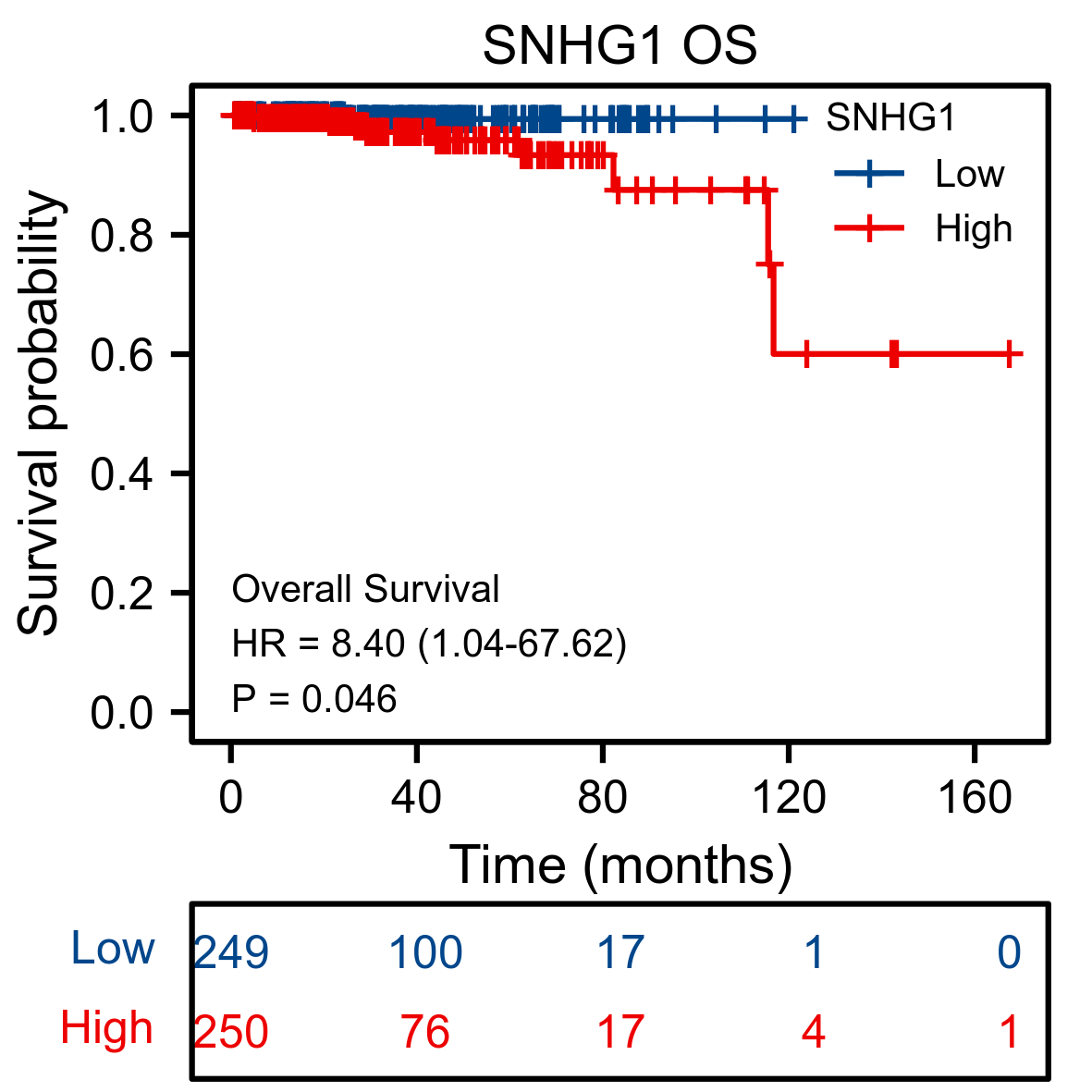

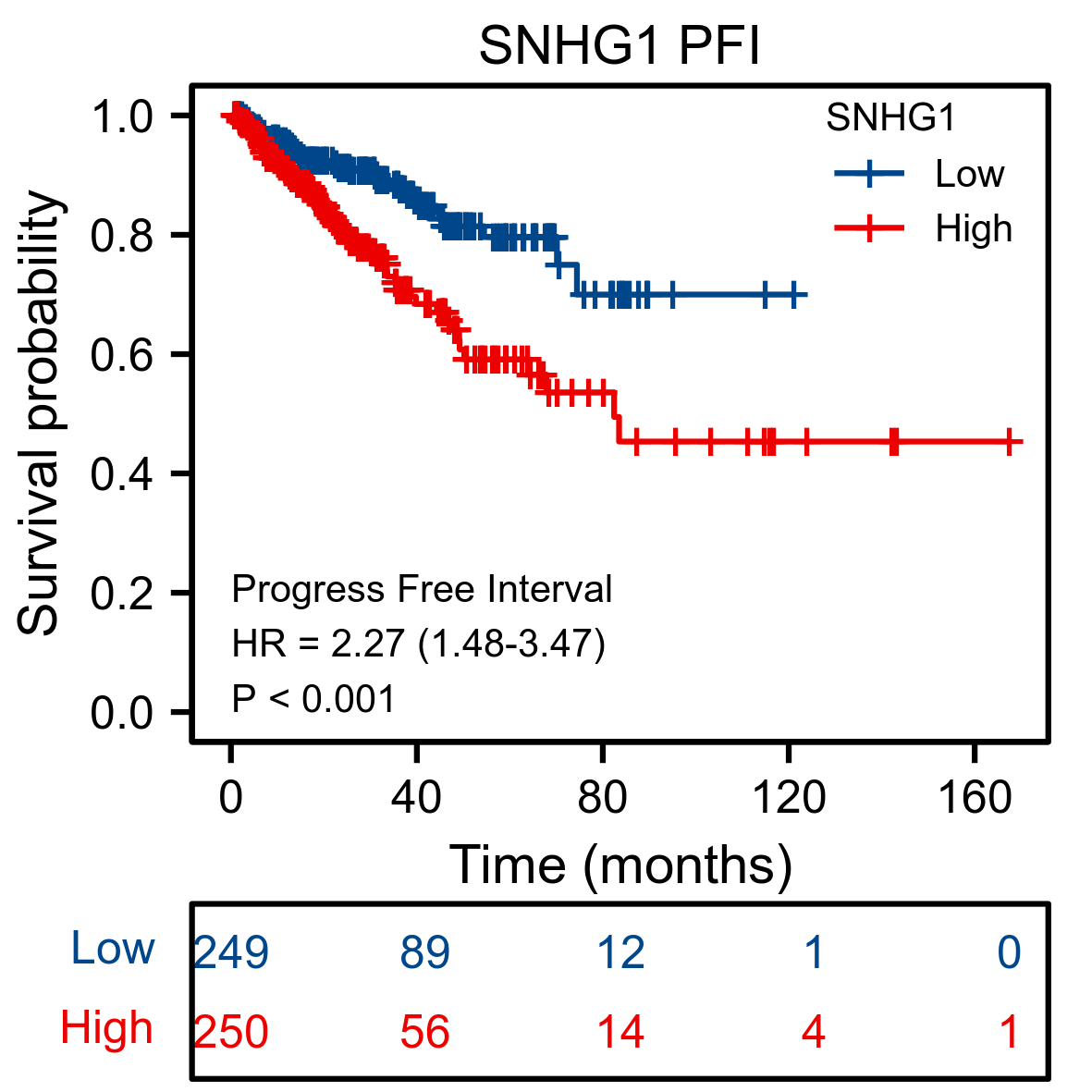

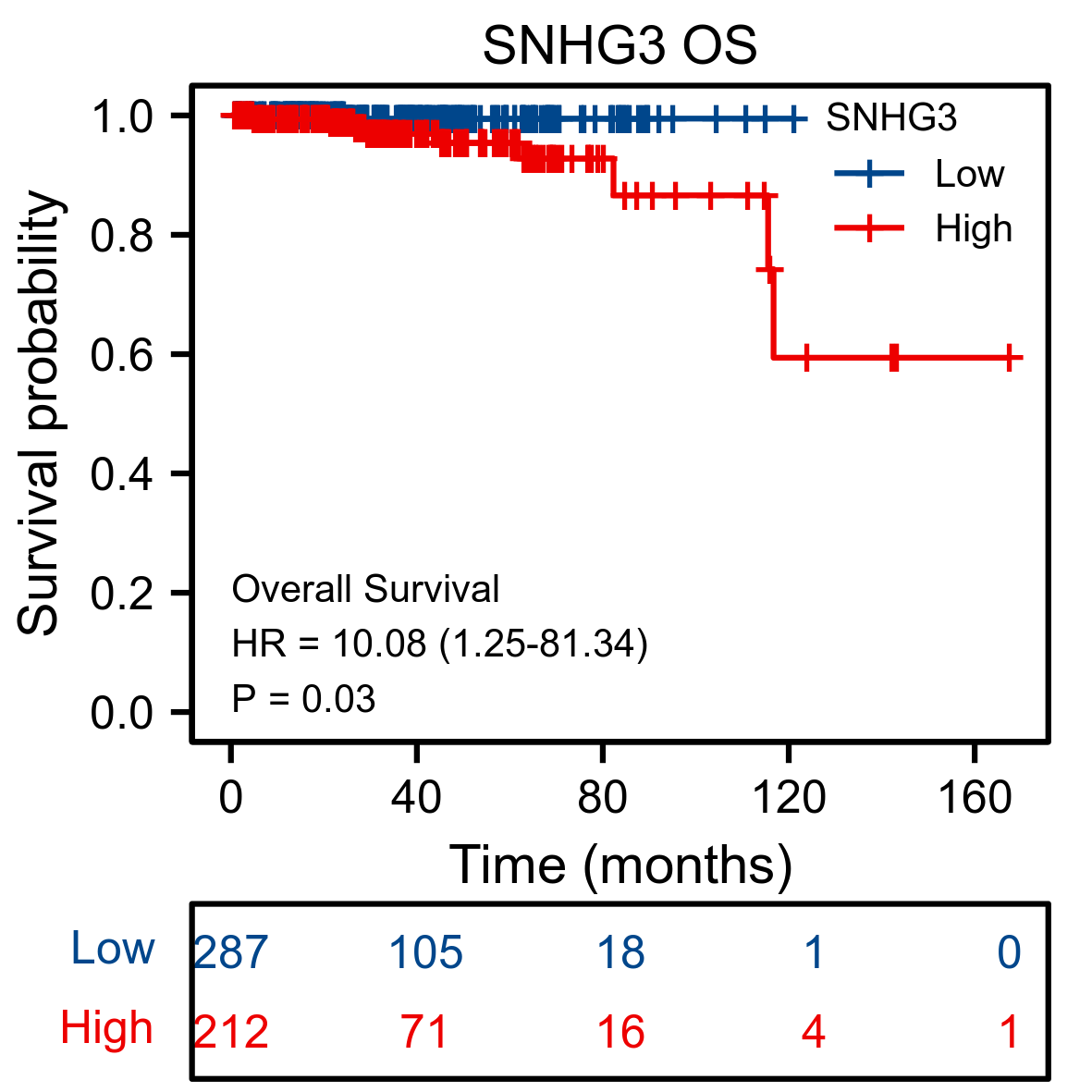

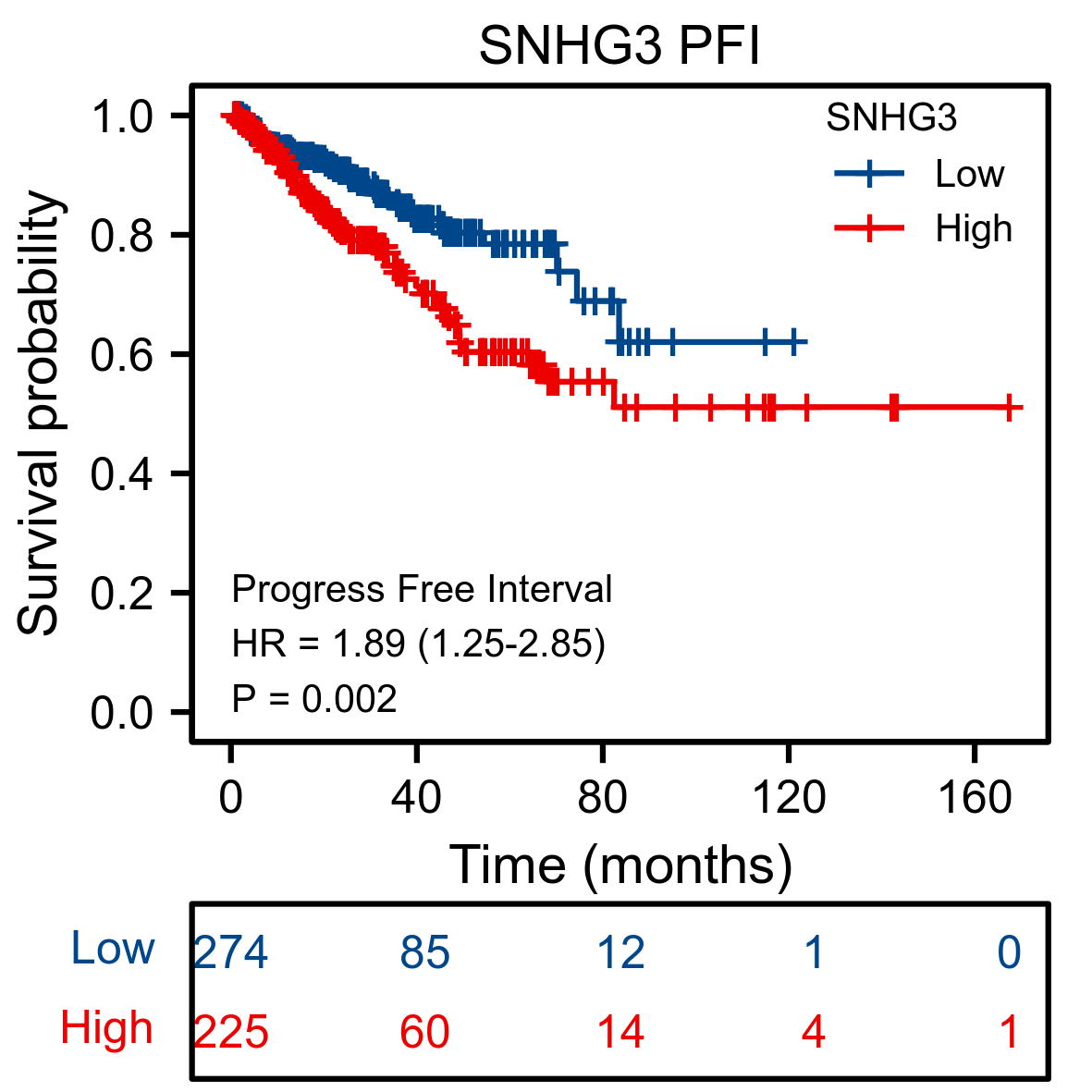

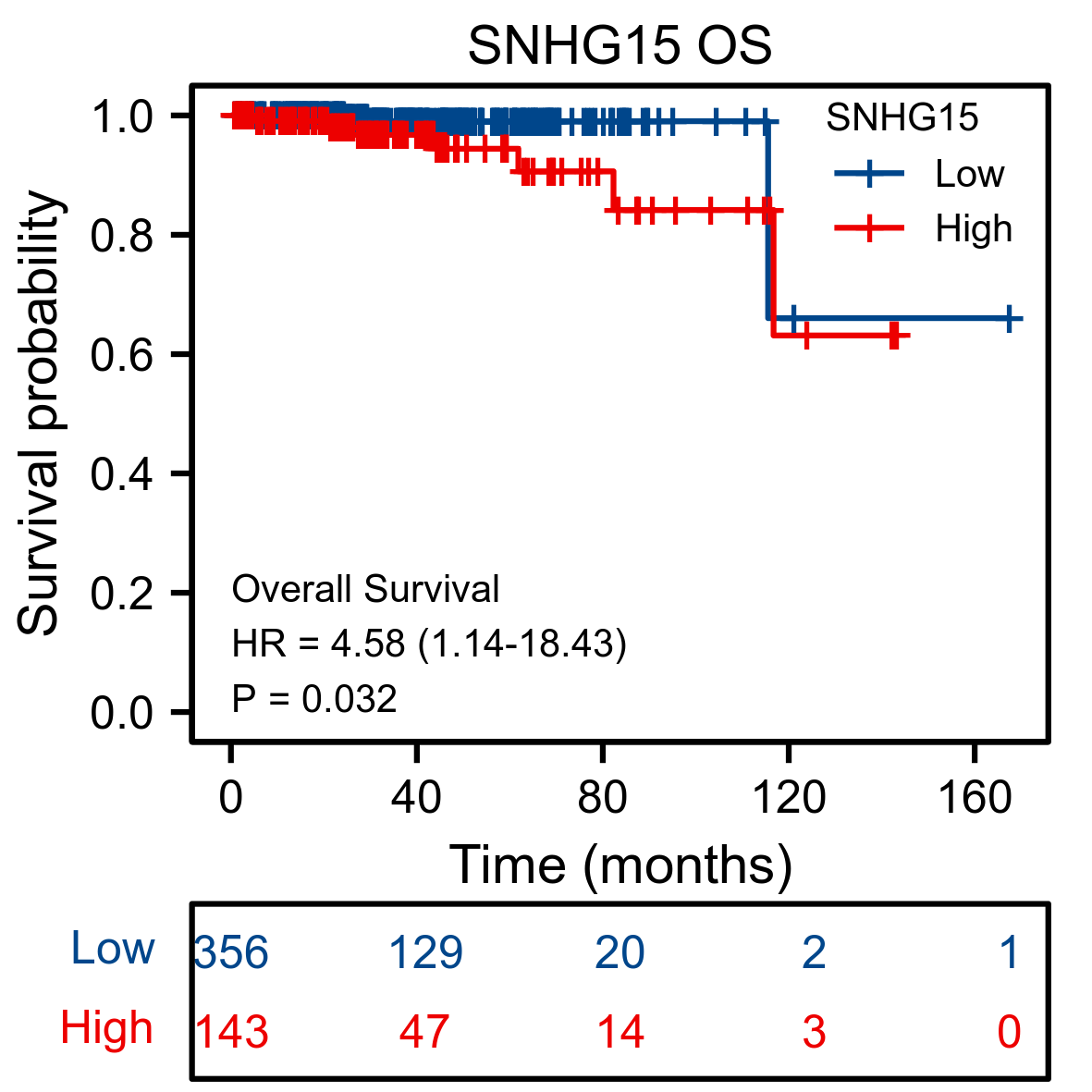

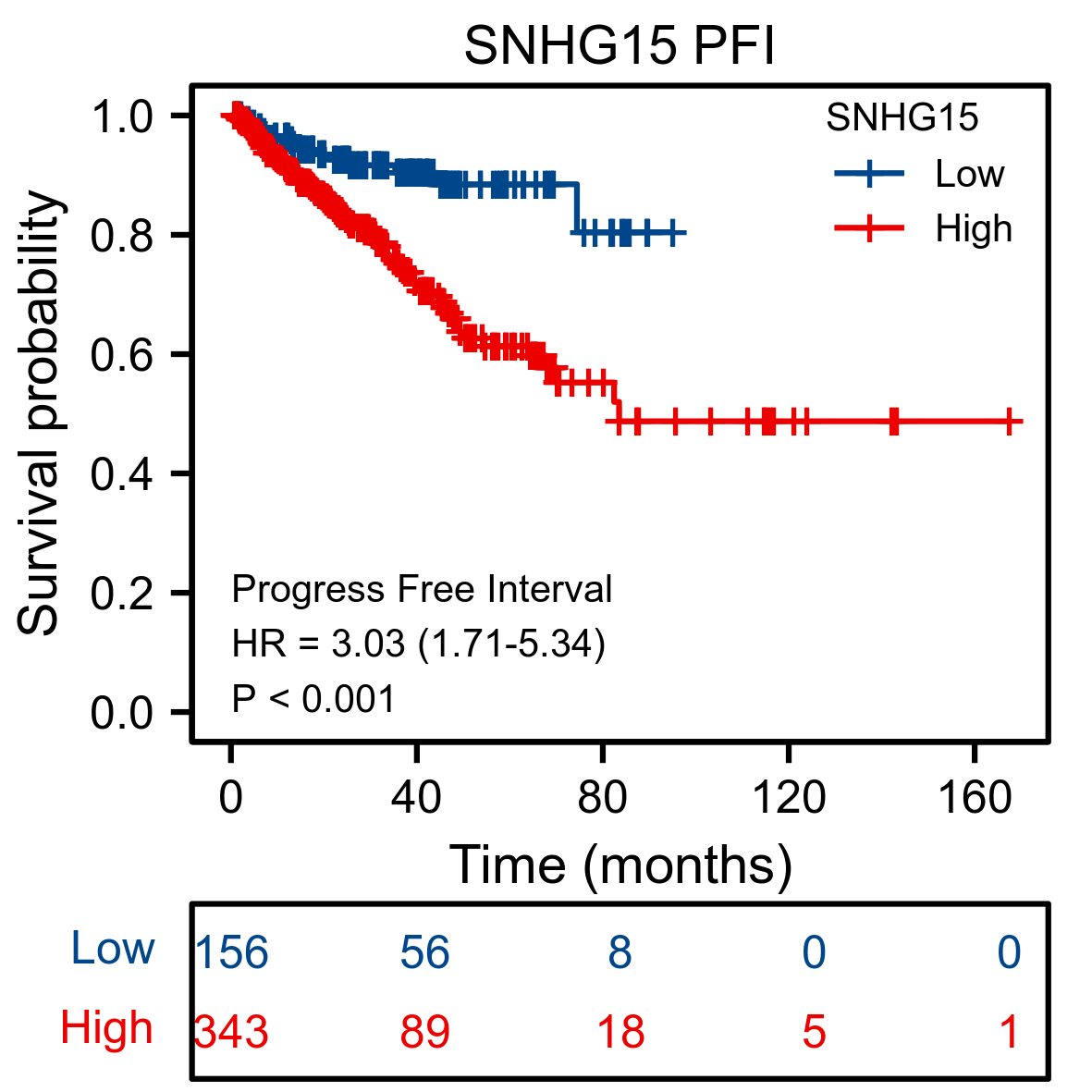

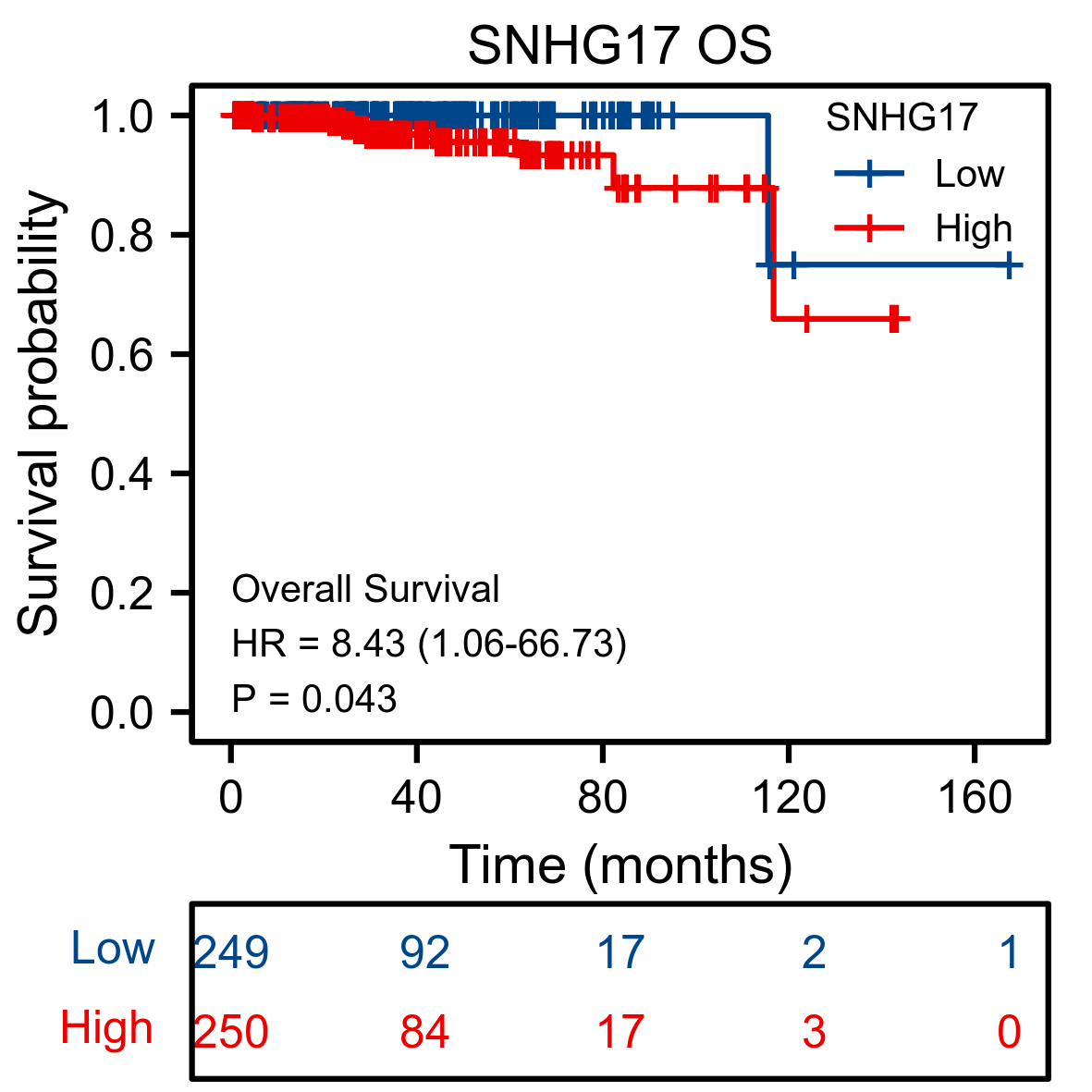

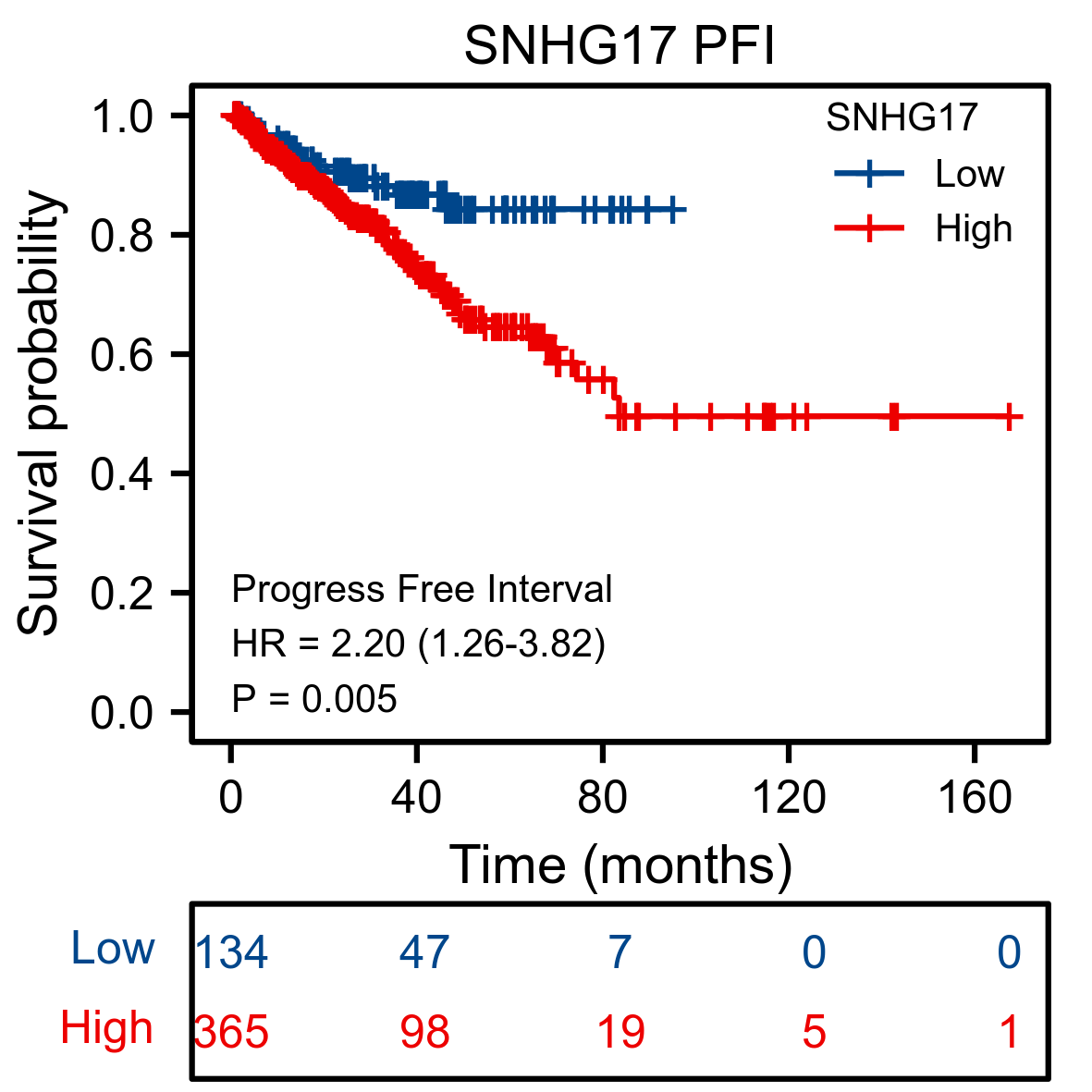

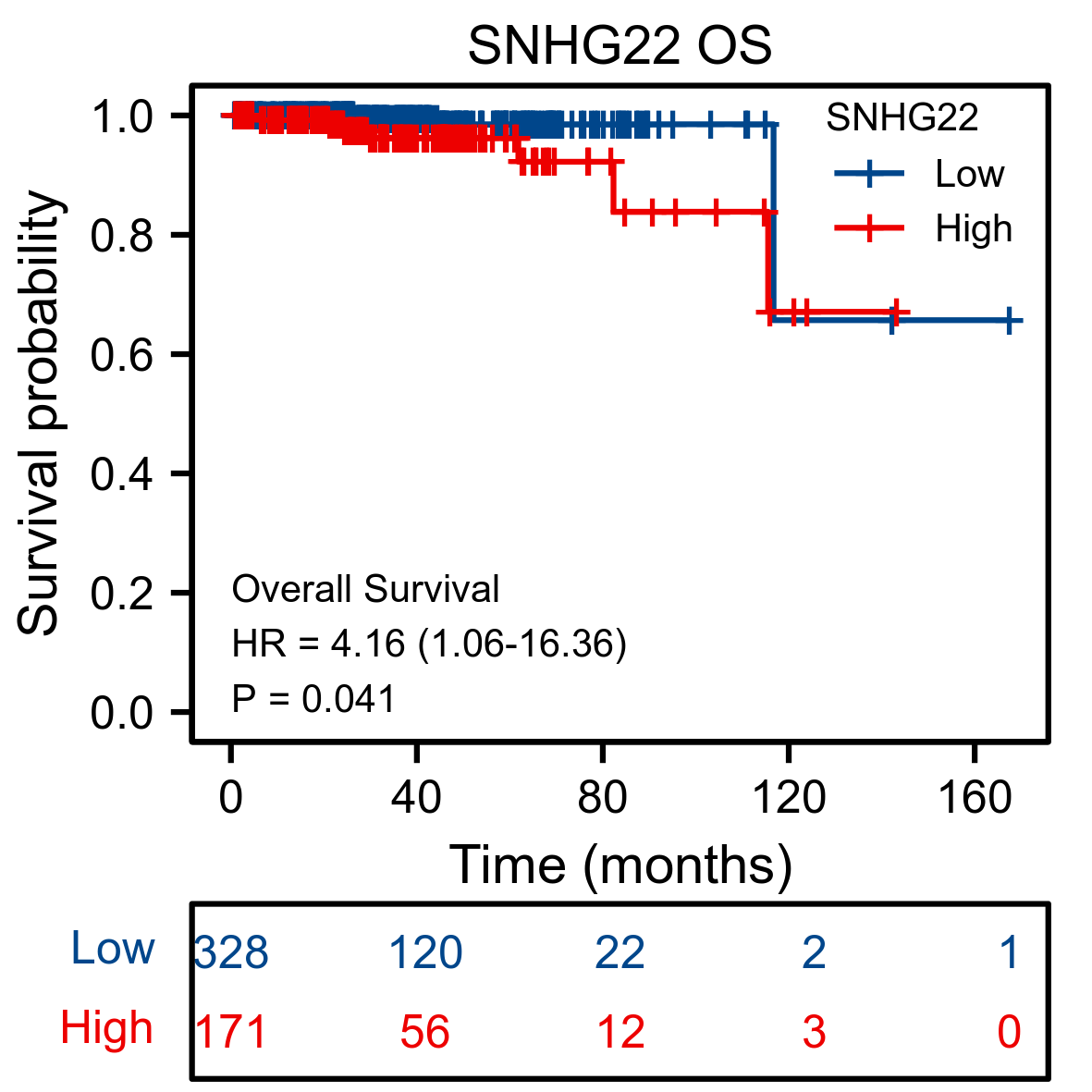

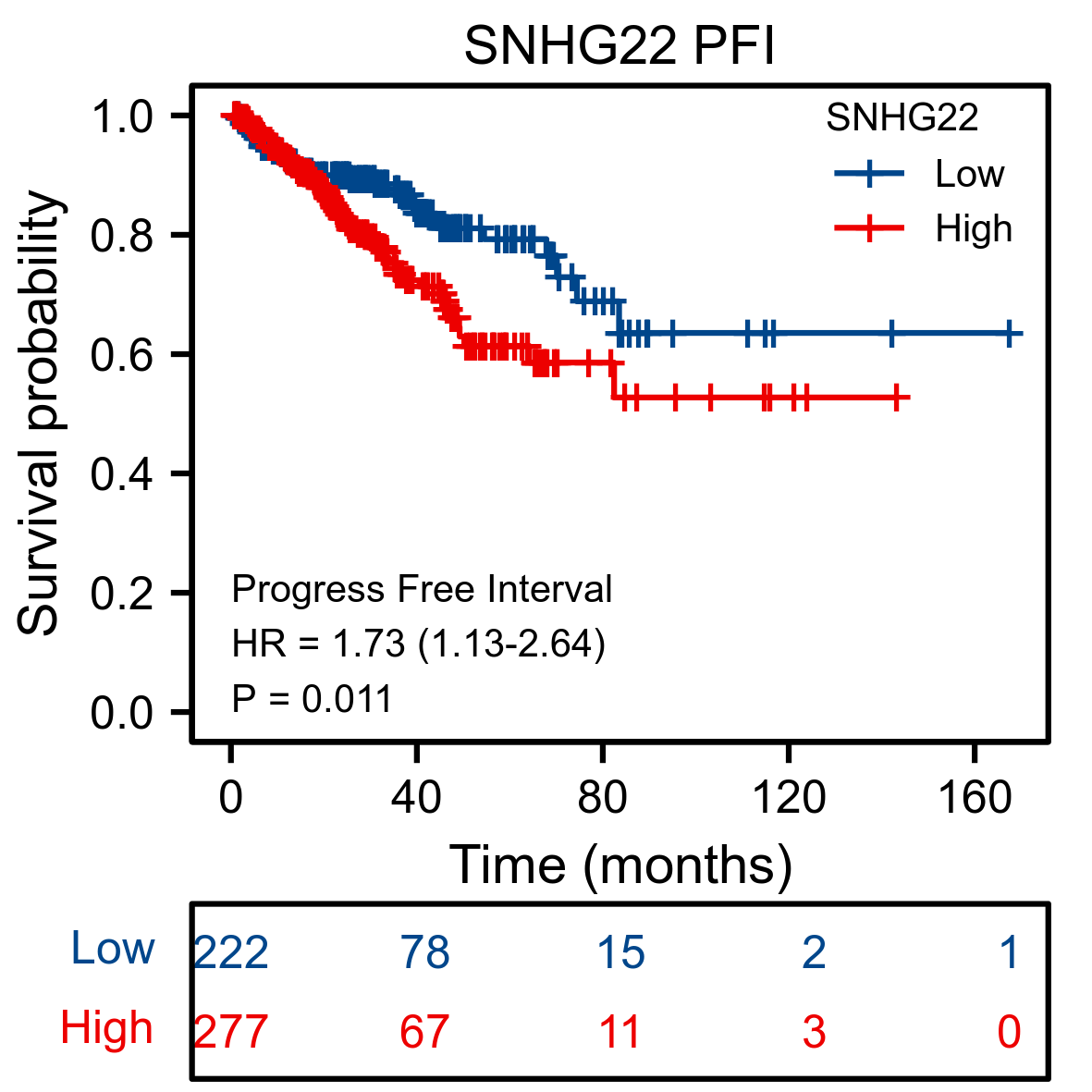

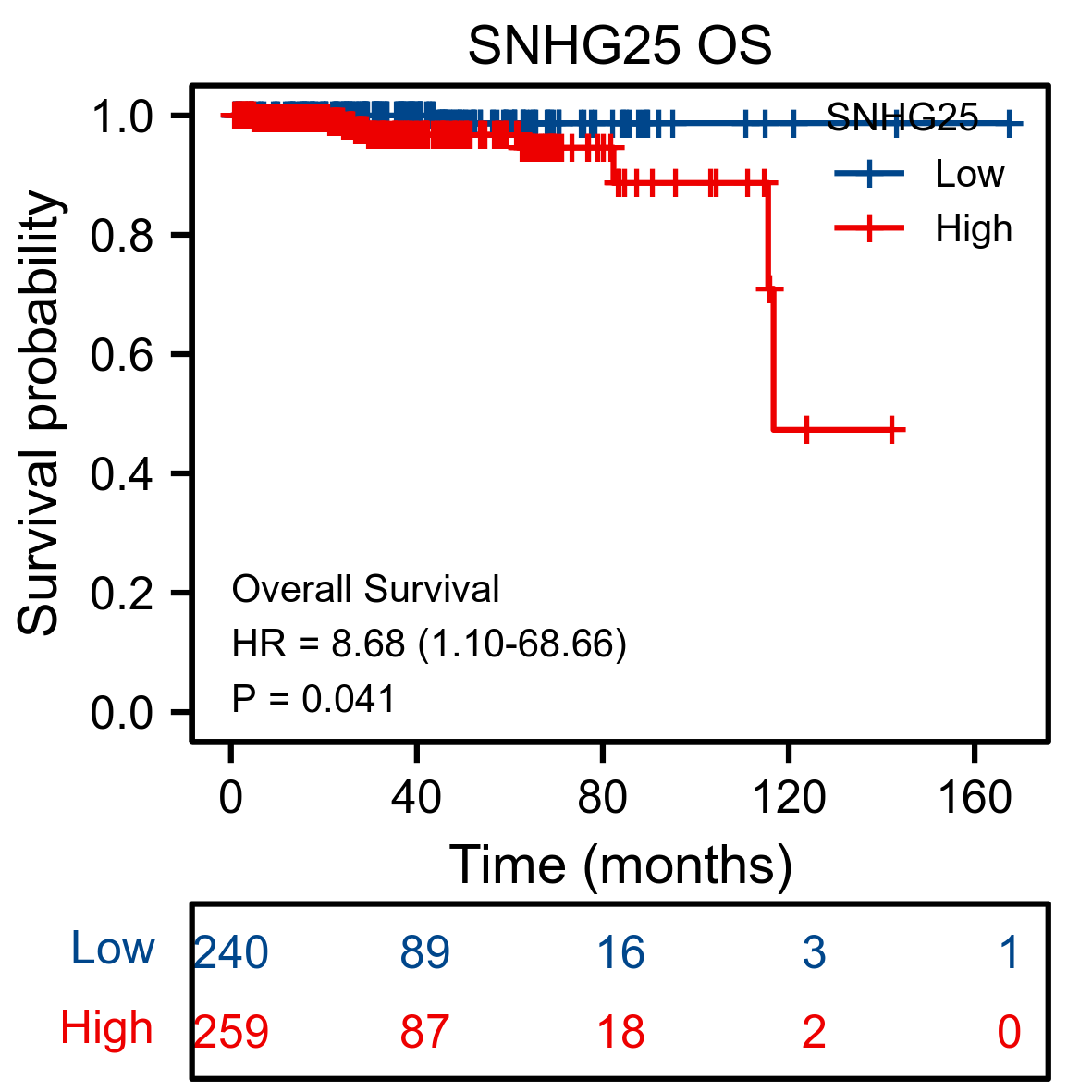

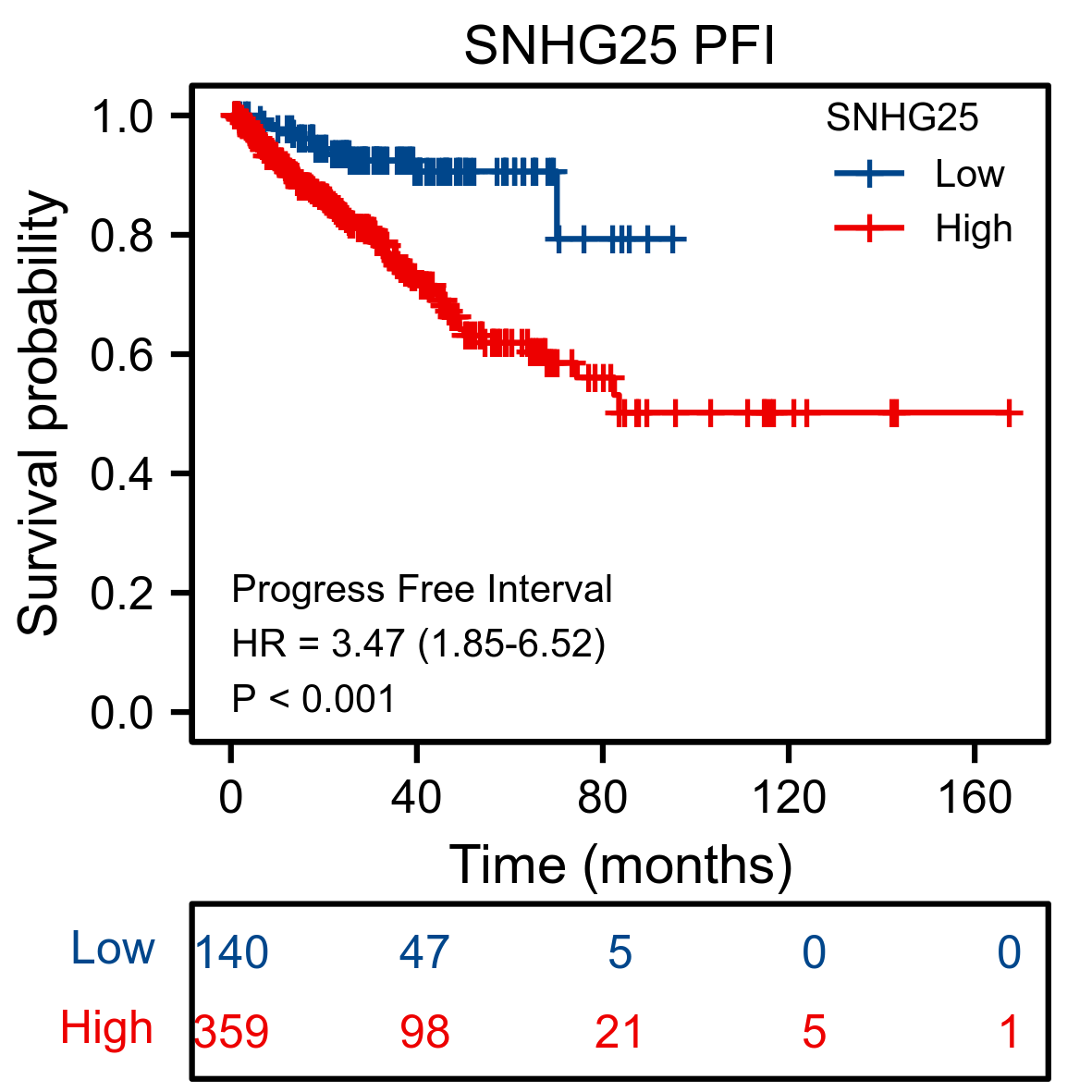


**A**

**B**

**C**

**D**

**E**

**F**

**G**

**H**

**I**

**J**

**K**

**L**

**Supplementary Figure 1. KM plotters indicating the relationship between SNHGs and OS/PFI of PC patients.**

**(A - L)** KM plotters indicating the relationship between SNHGs expression and OS/PFI of PC patients, the cut-off values for high/low SNHGs expression were set as the best separate for each SNHG.

**Supplementary Figure 2. Relationship between immune infiltration and expression of SNHG17 in prostate cancer.**


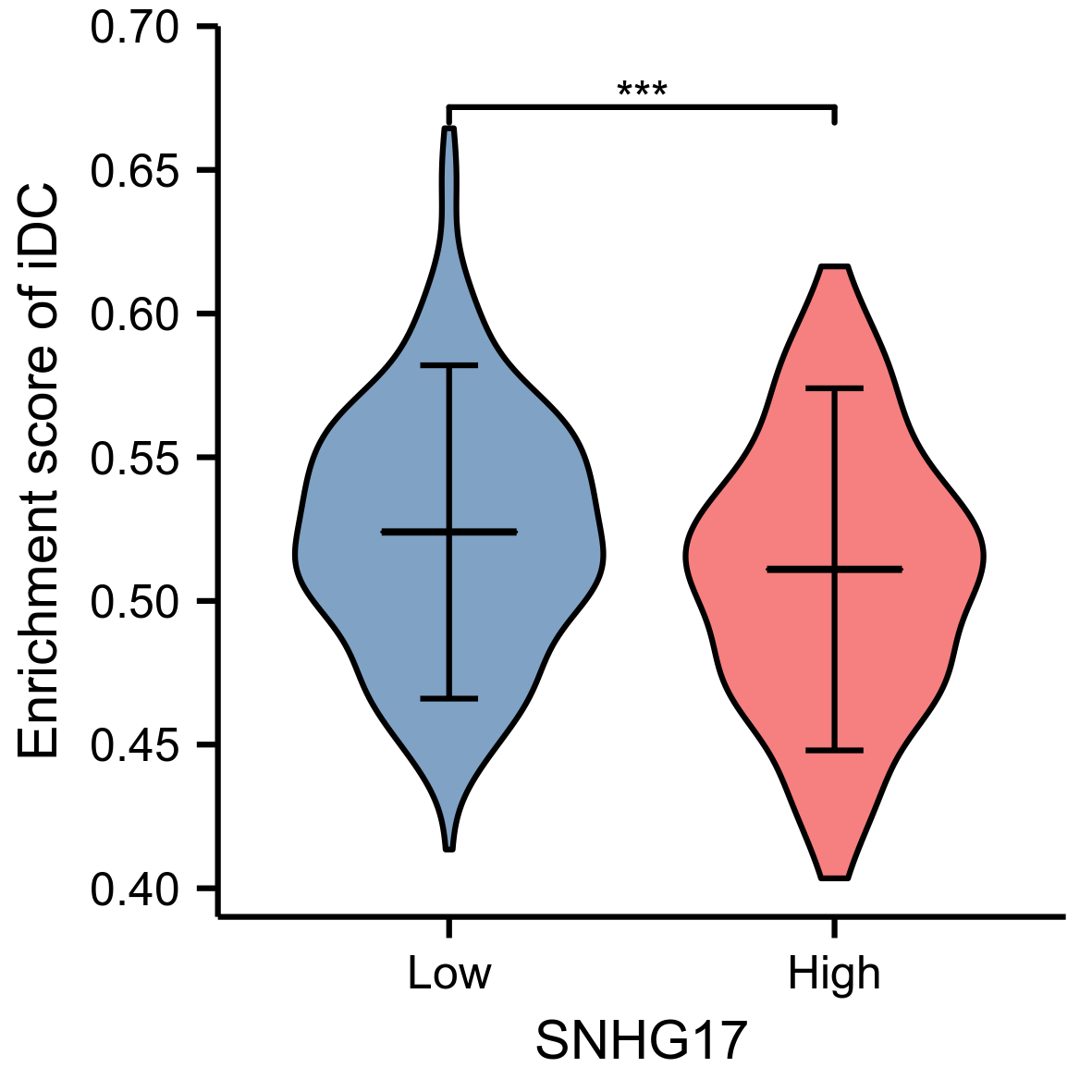

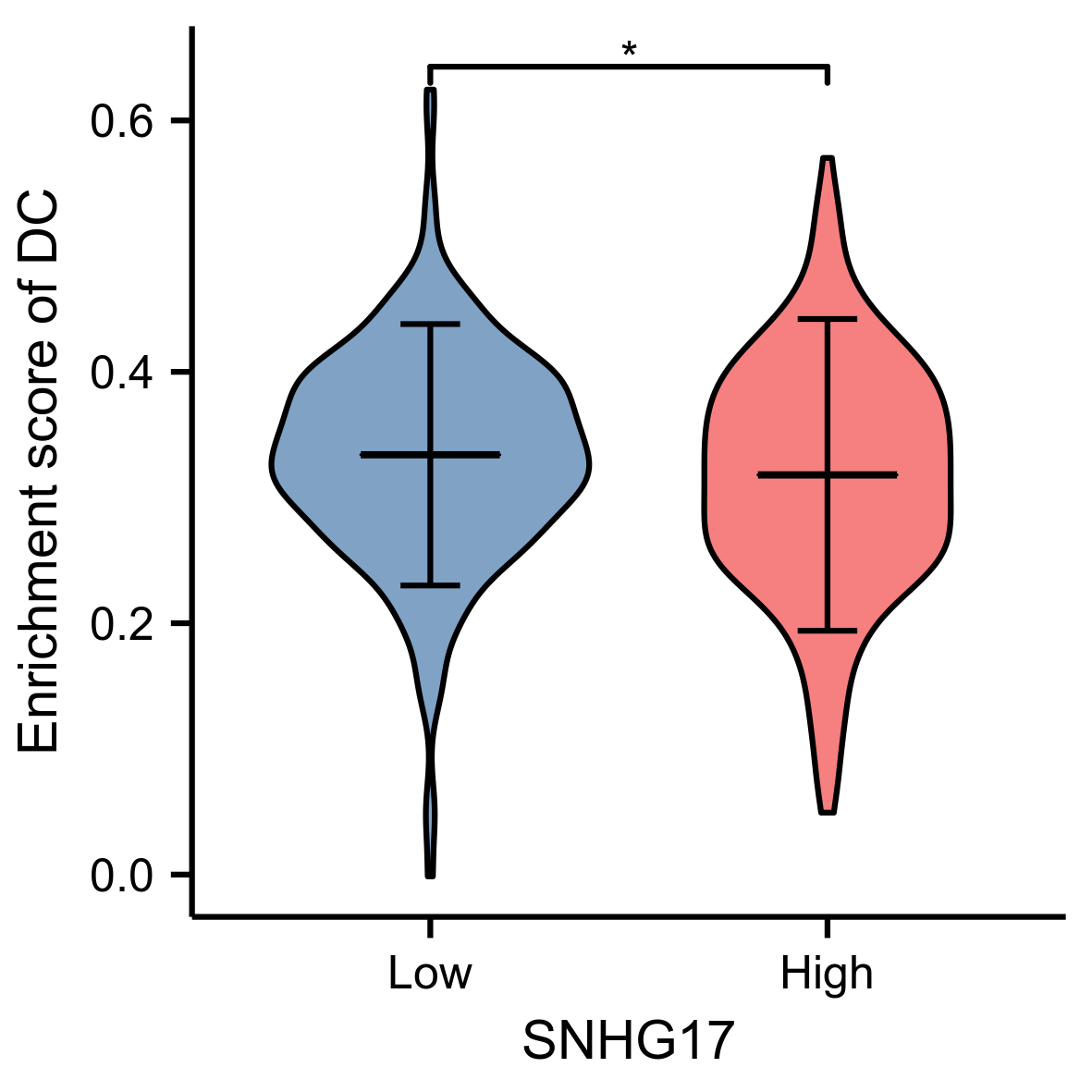

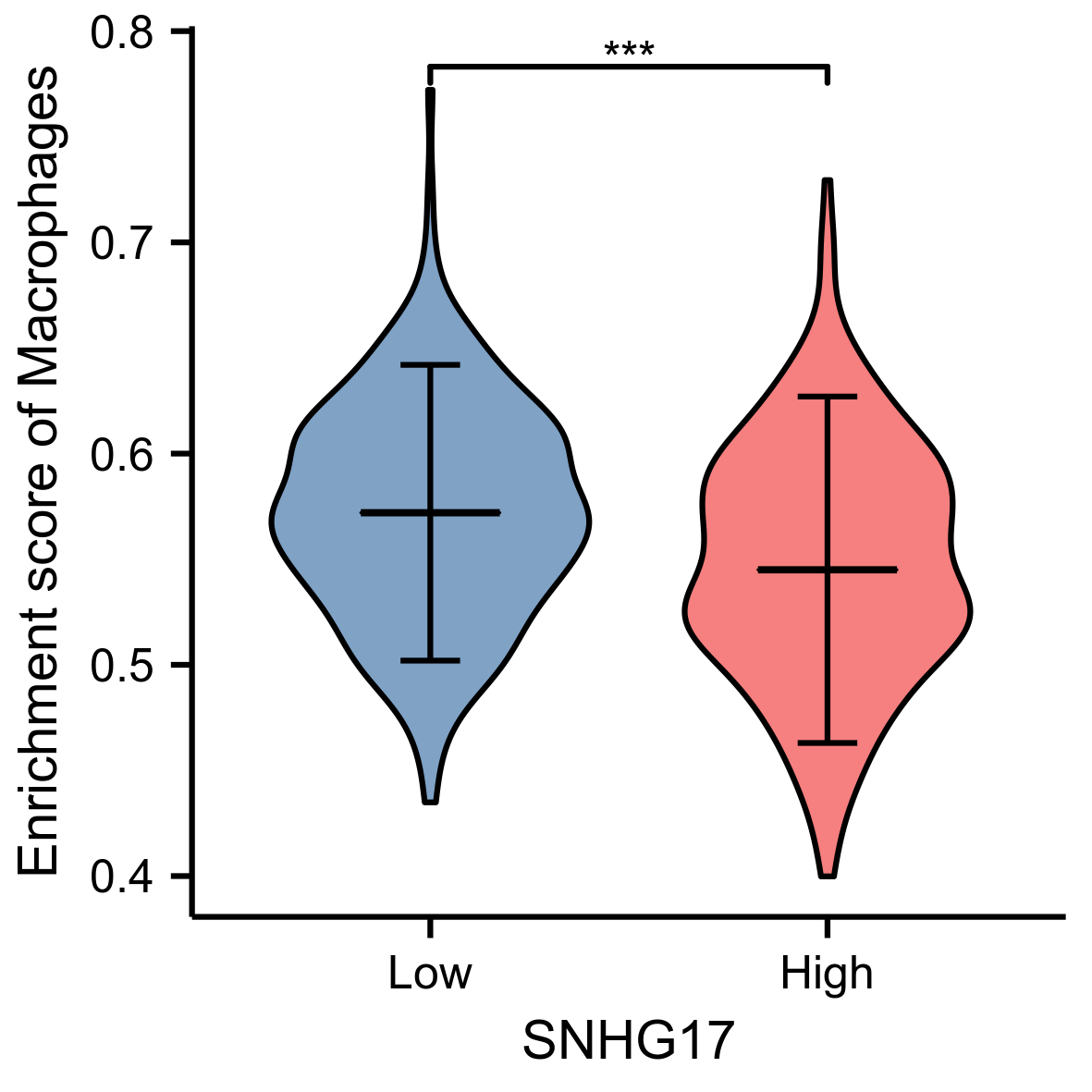

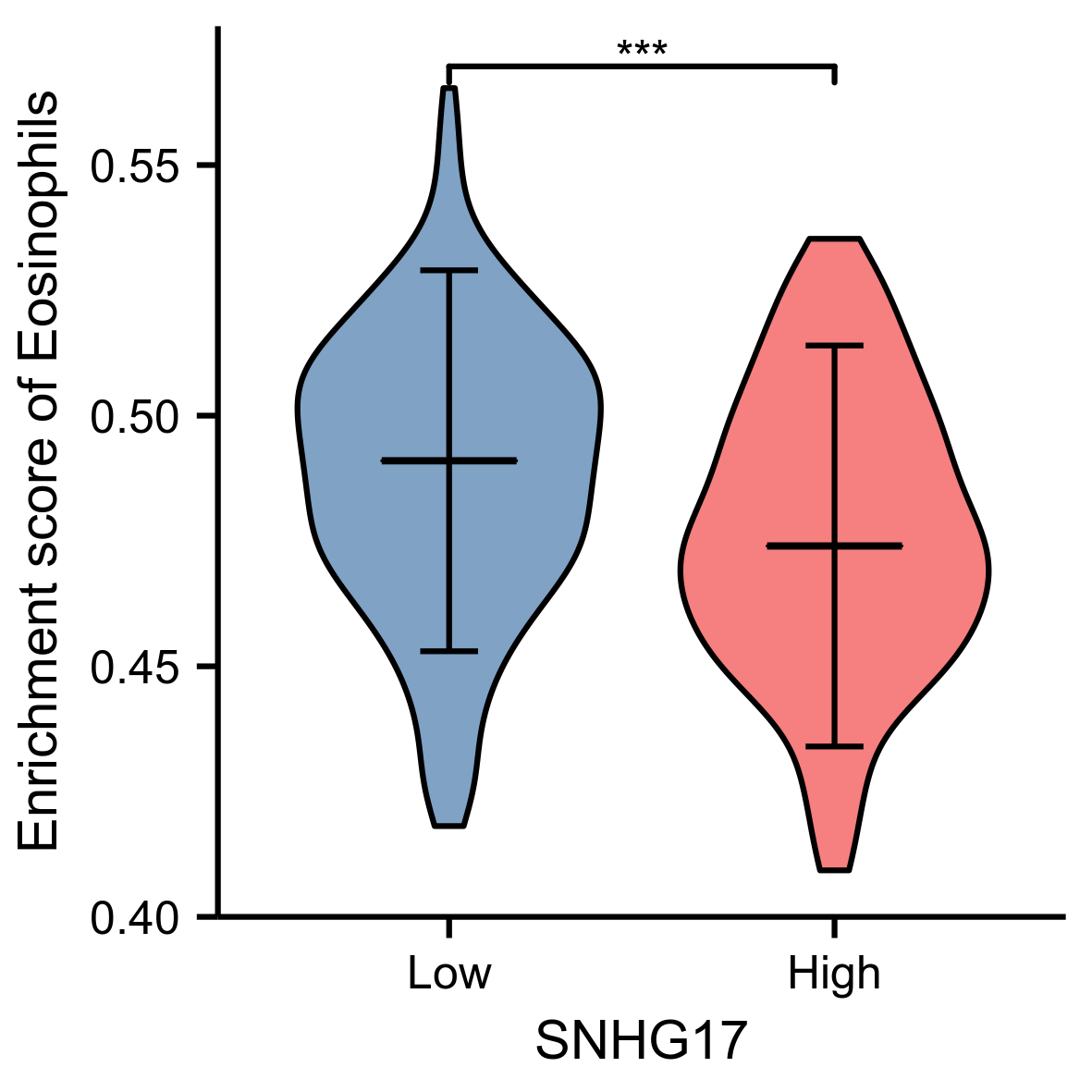

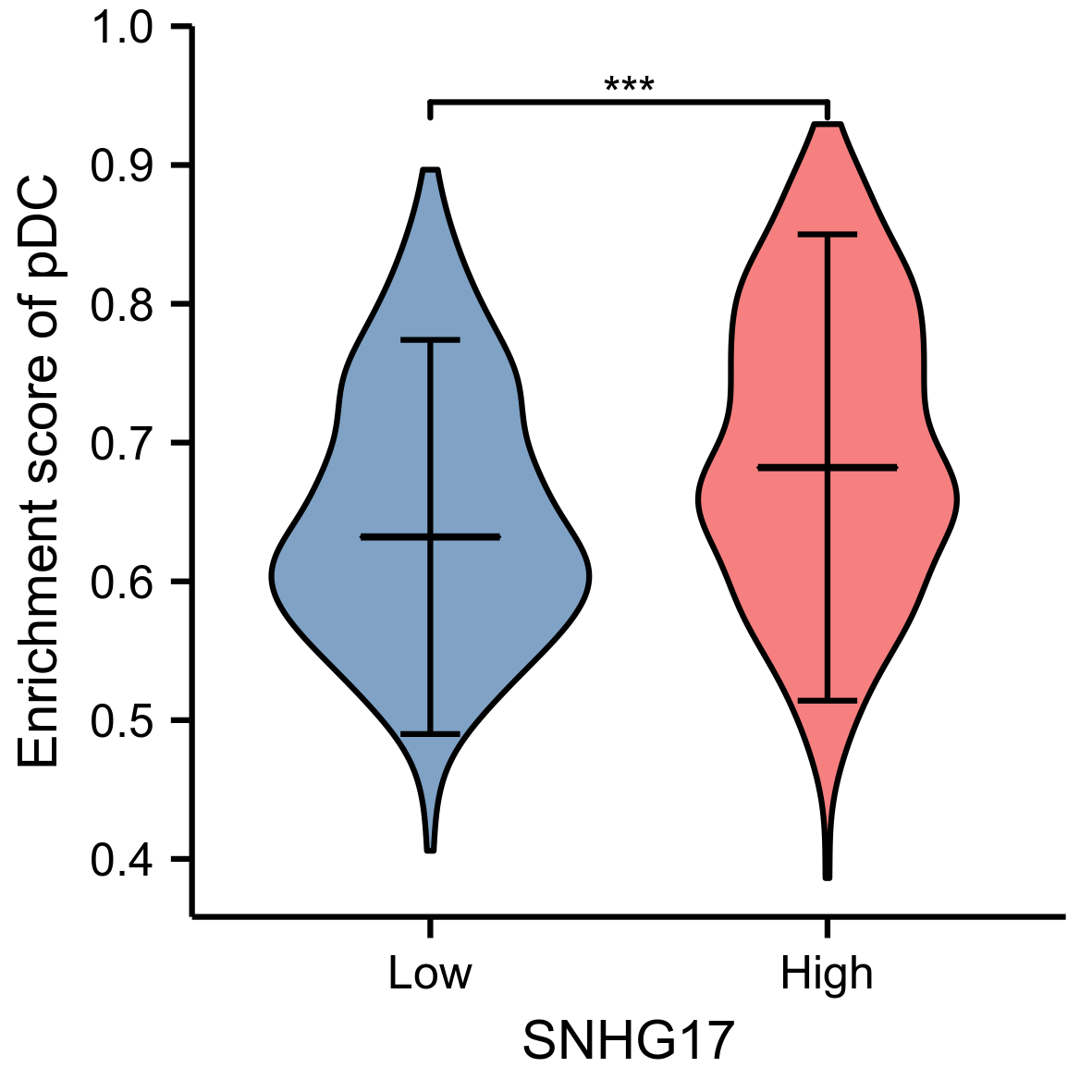

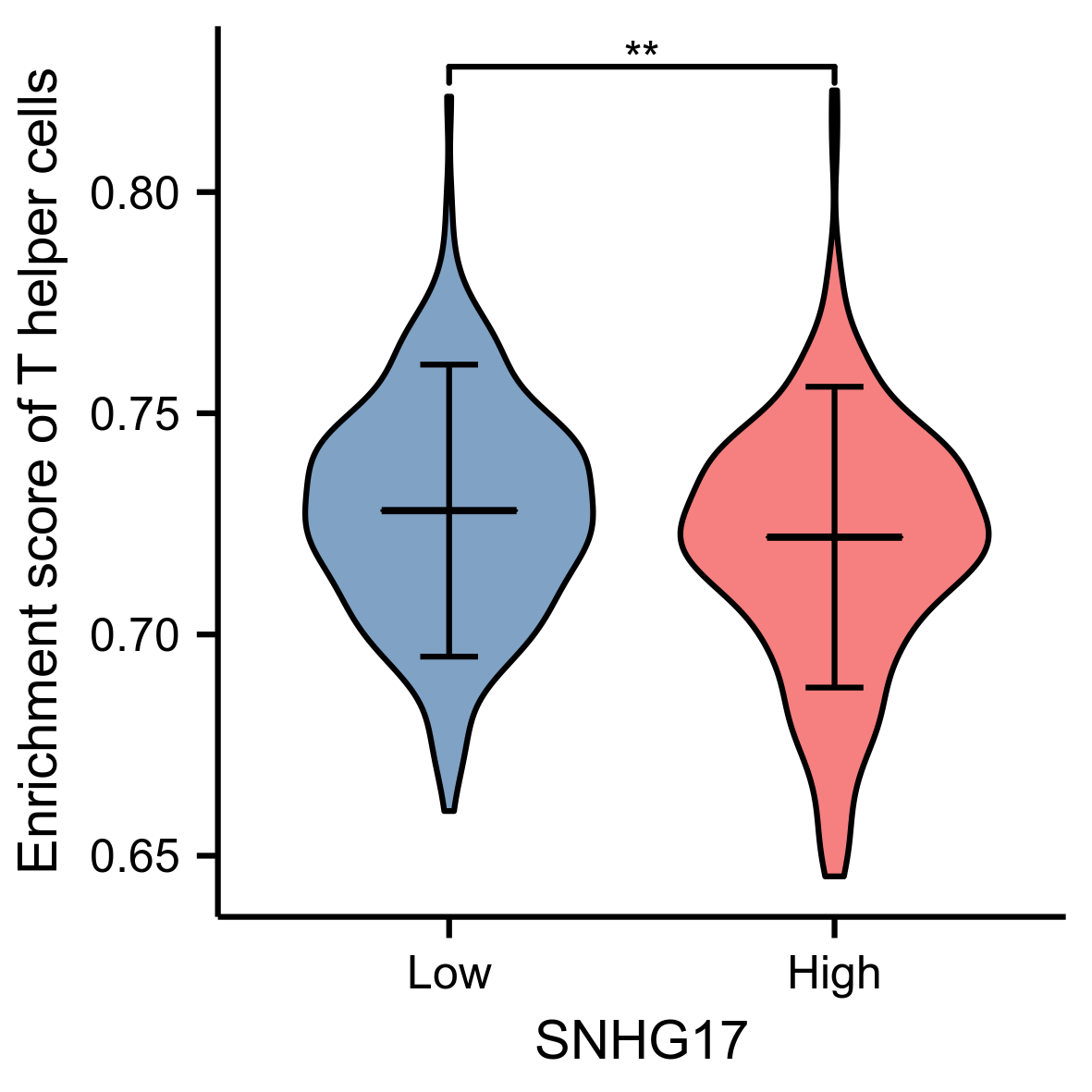

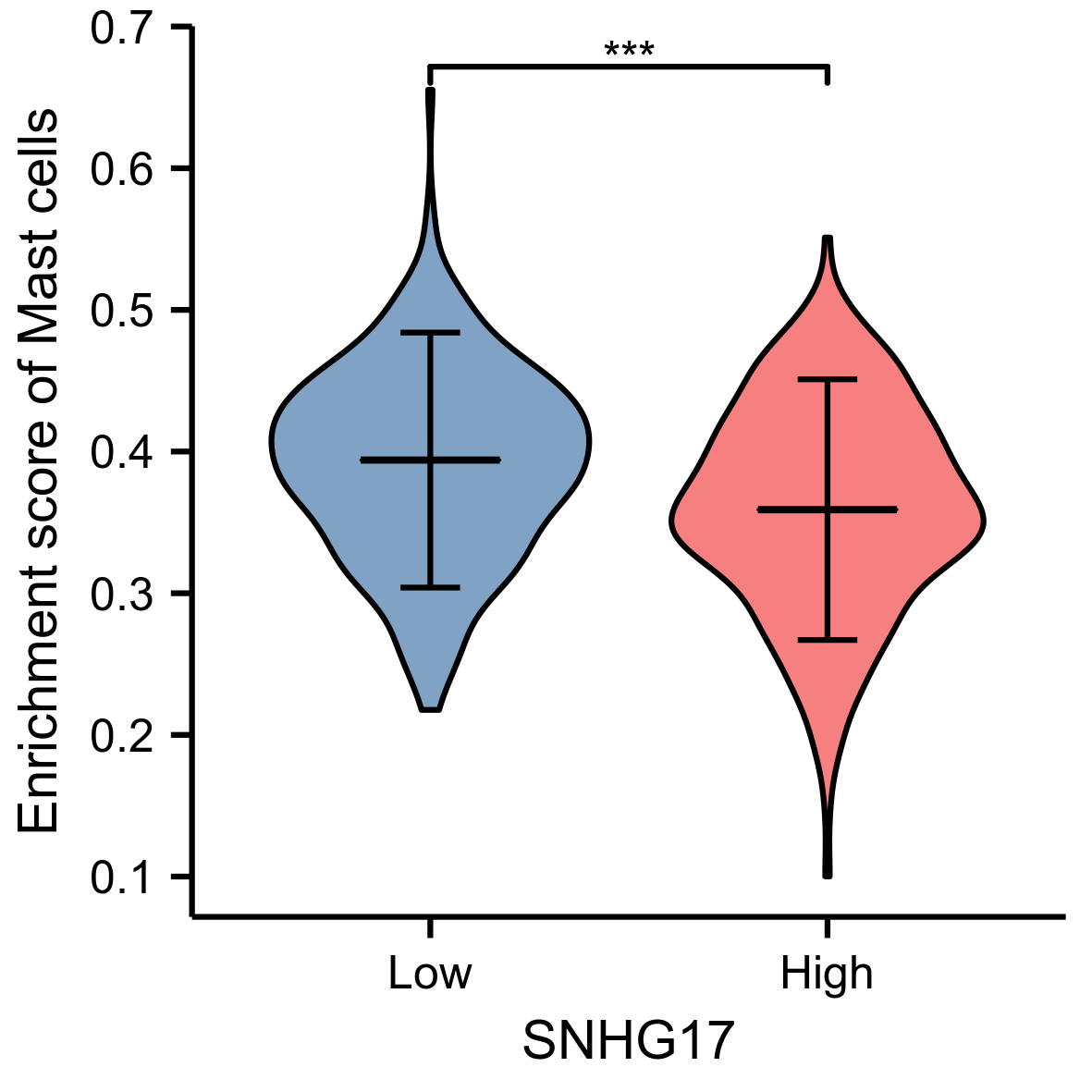

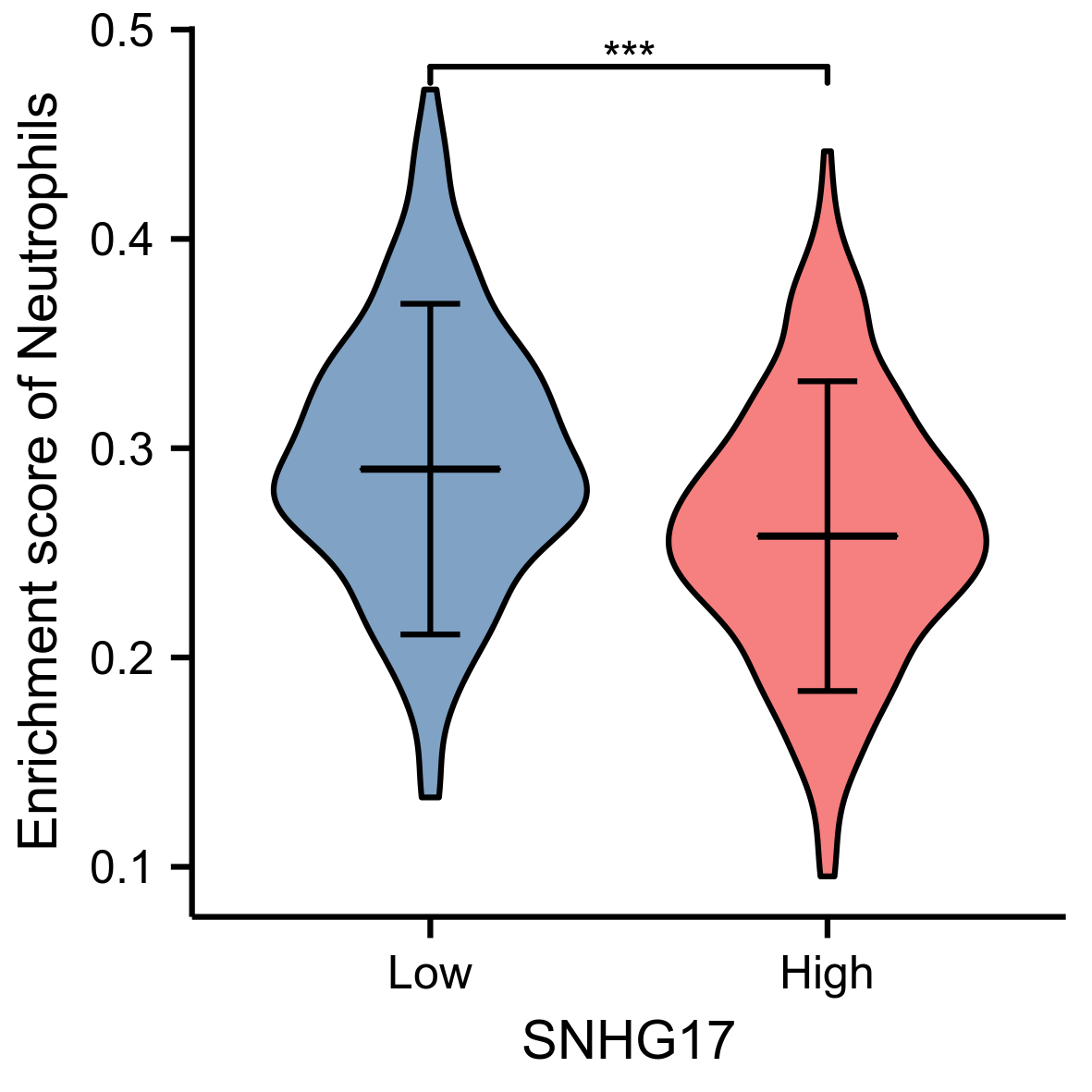

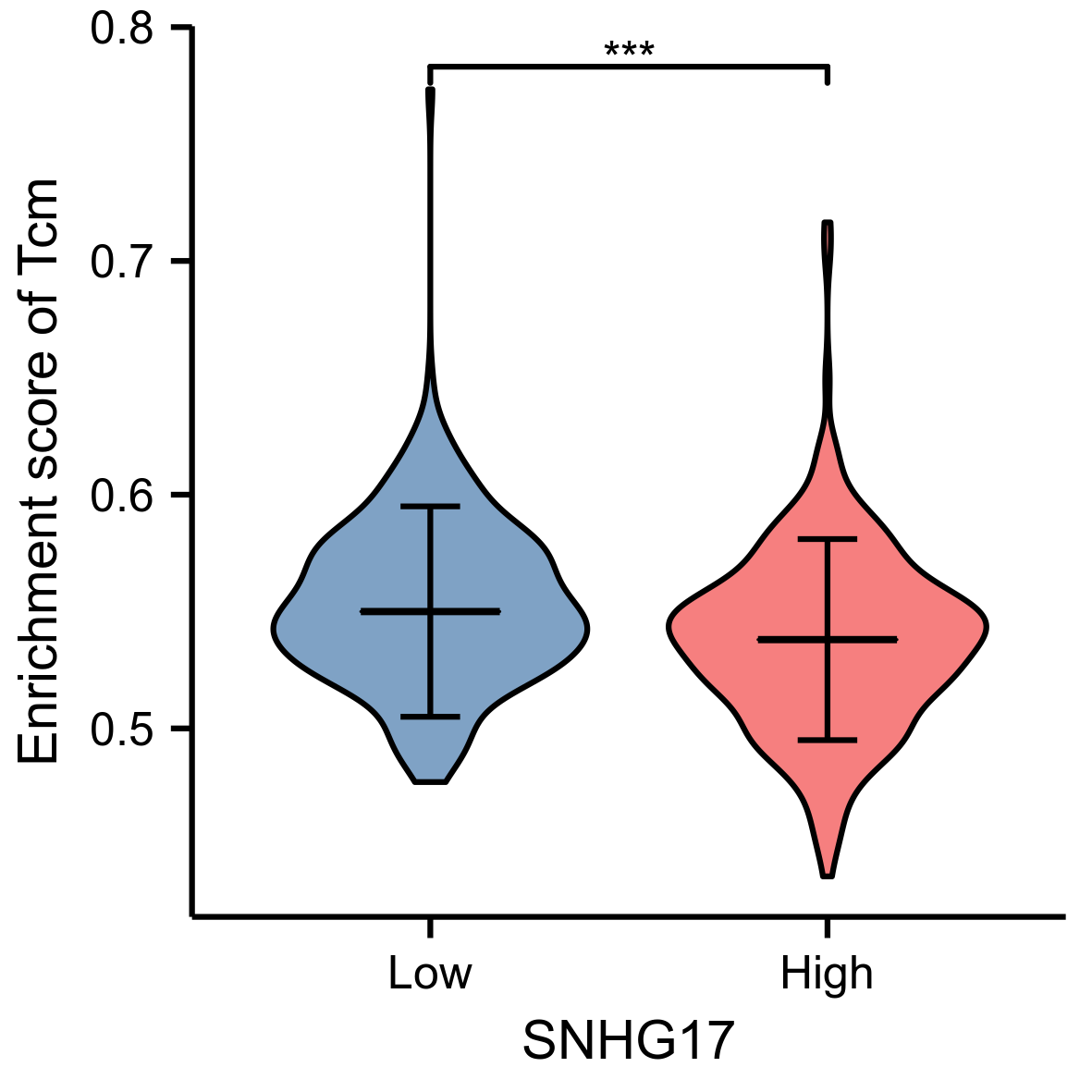

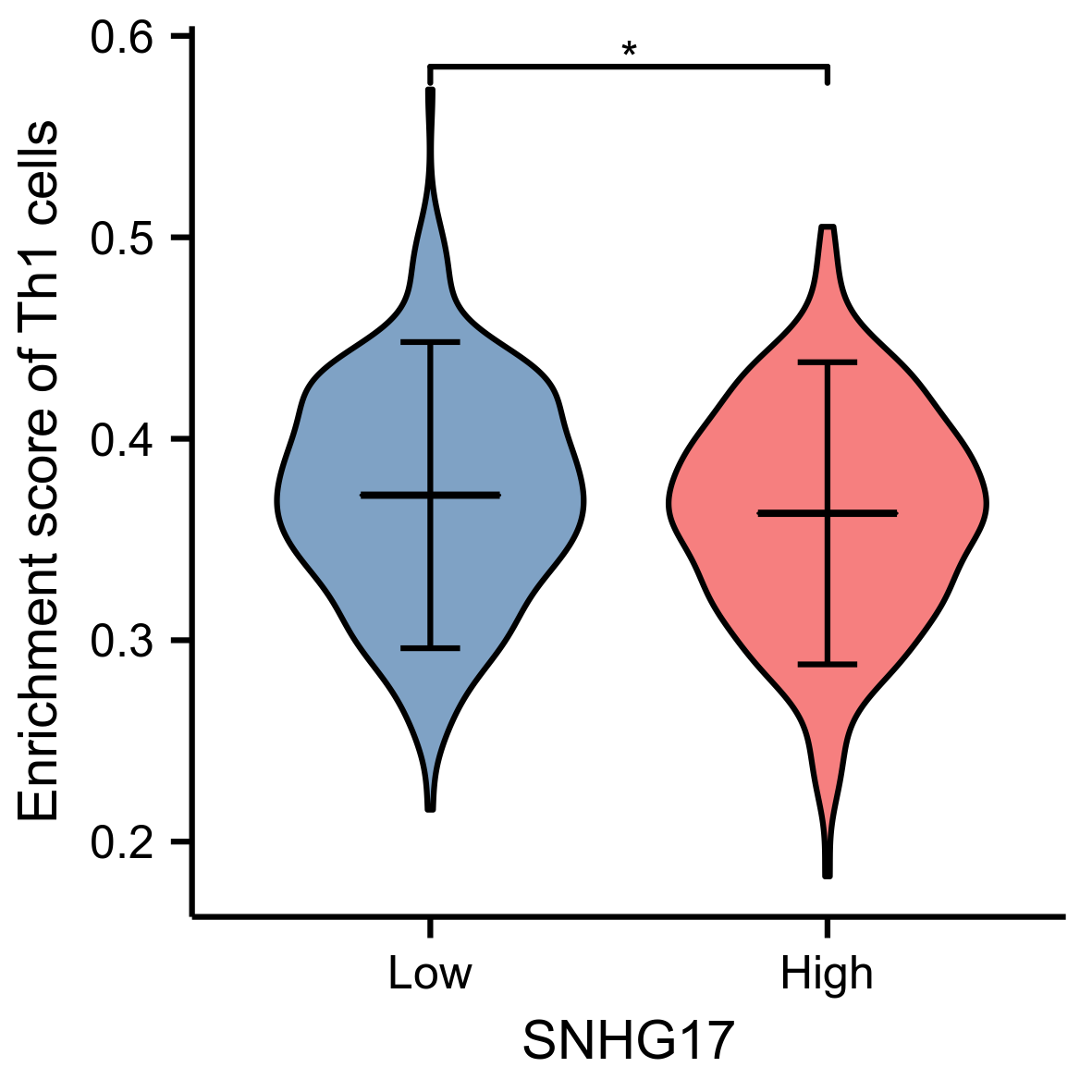

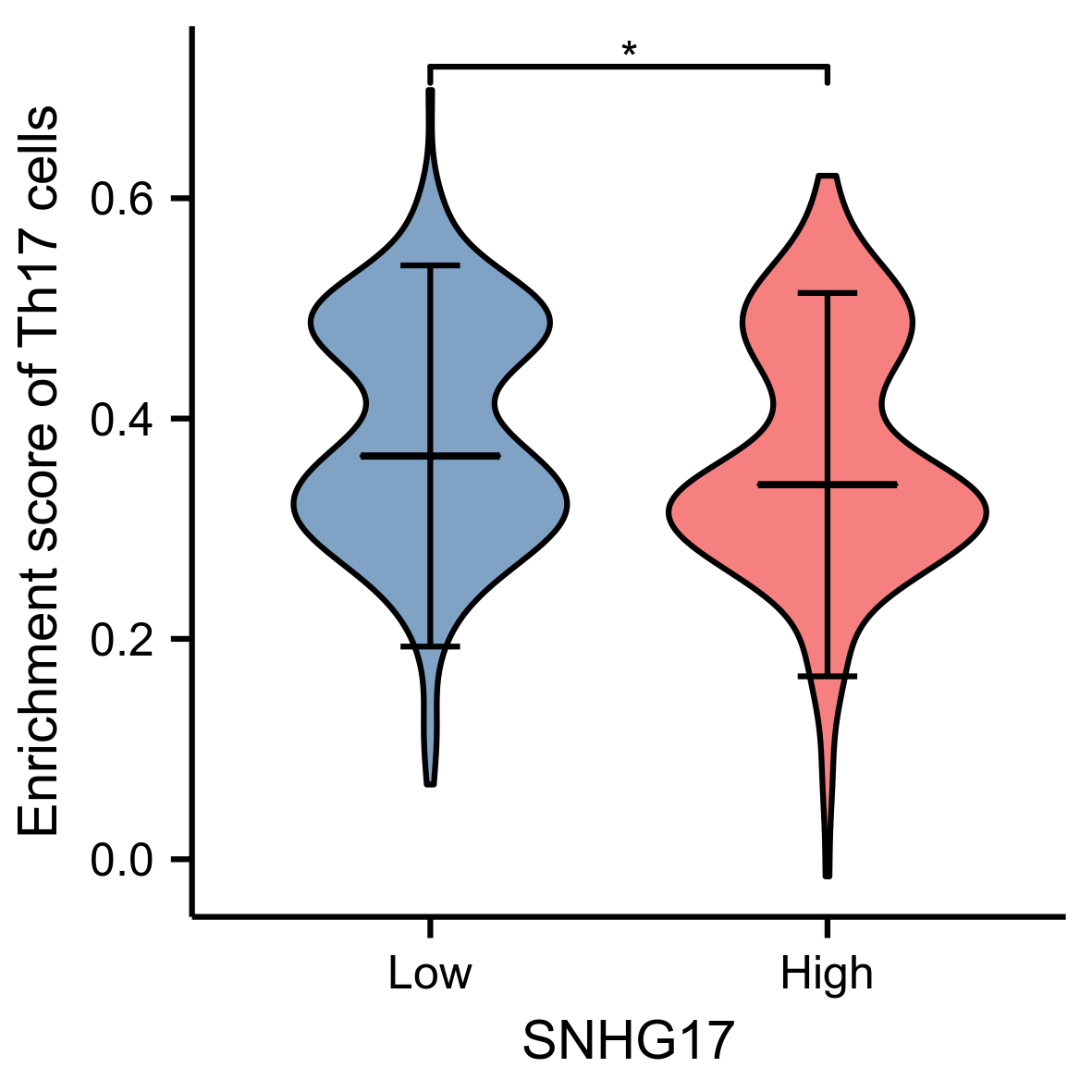

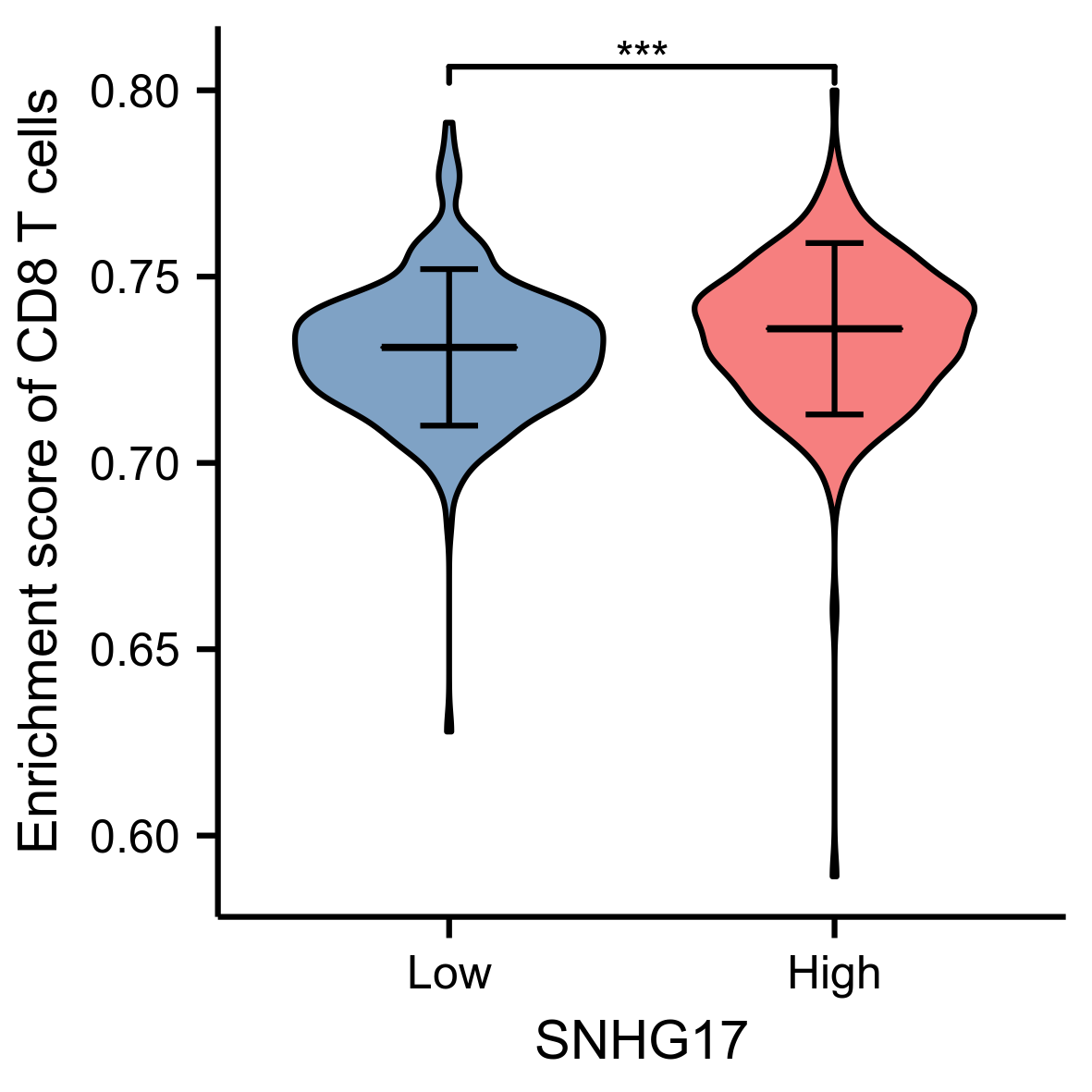


**A**

**B**

**C**

**D**

**E**

**F**

**G**

**H**

**I**

**J**

**K**

**L**

**(A - L)** Violin plots indicating the infiltration level of immune cells in different groups with low/high expression of SNHG17 in prostate cancer. The 499 patients were separated by the mean value of SNHG17 expression. * indicates *p* < 0.05, ** indicates *p* < 0.01, *** indicates *p* < 0.001.

**Supplementary Figure 3. Implication of potential miRNAs in PC progression and GO/KEGG analysis of miRNAs.**


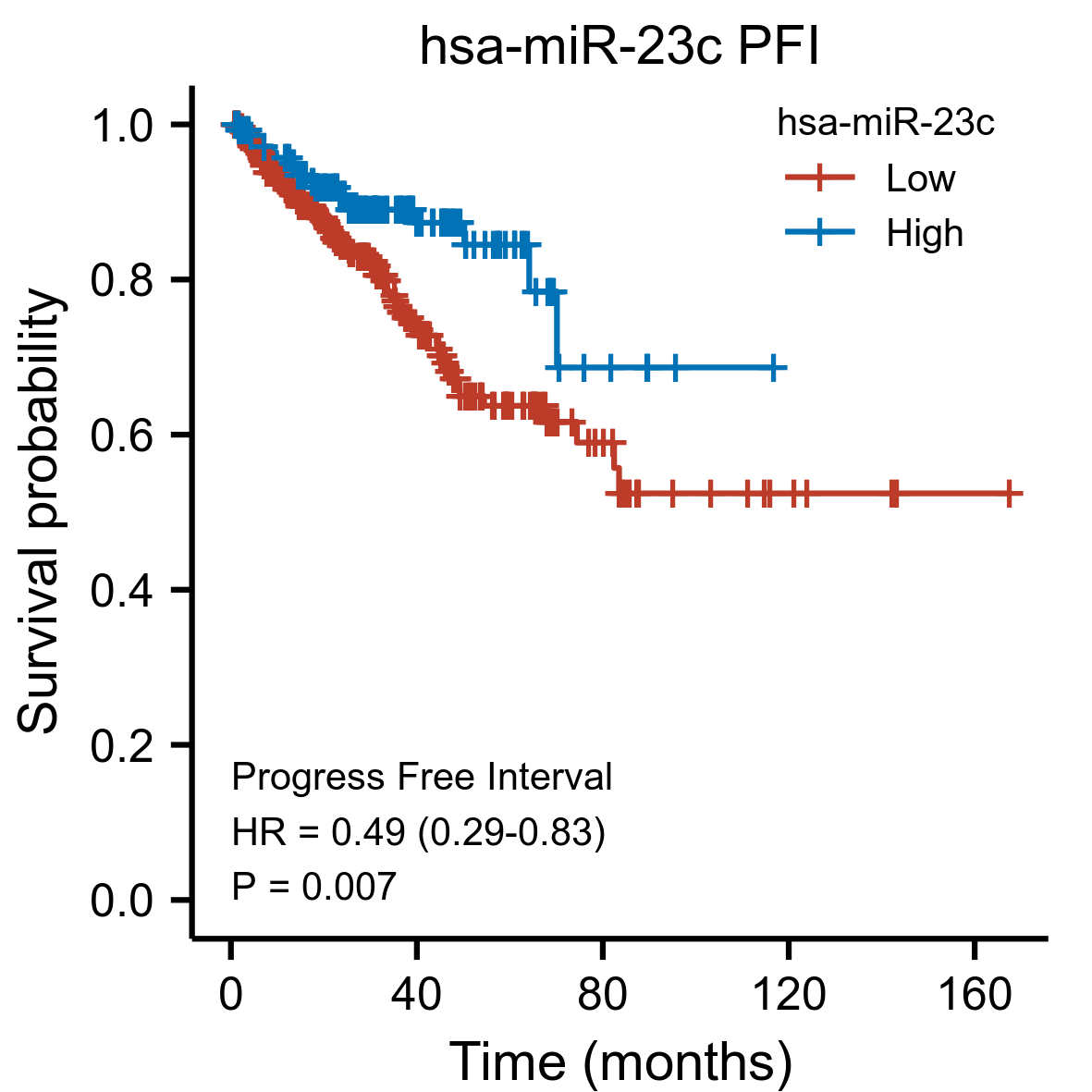

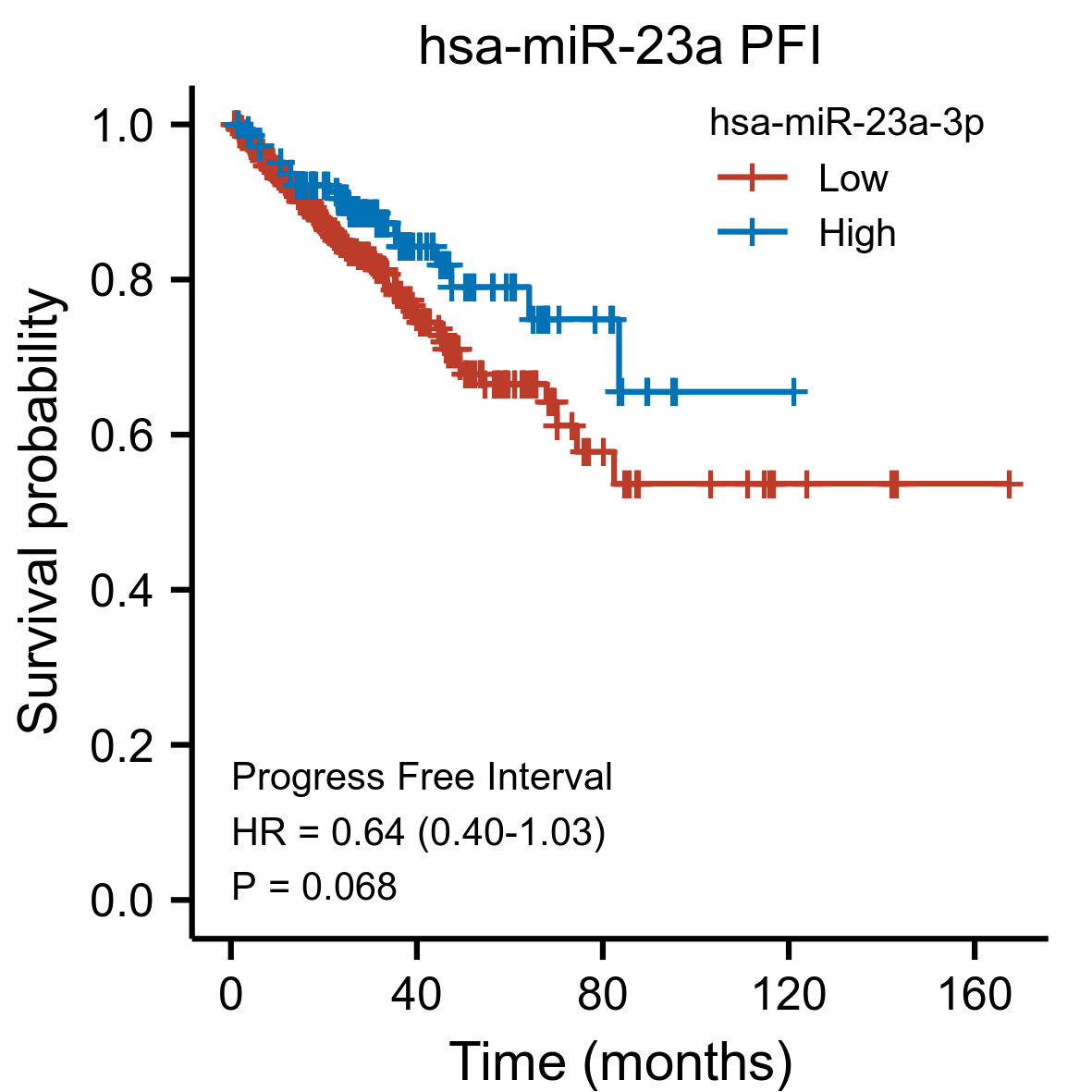

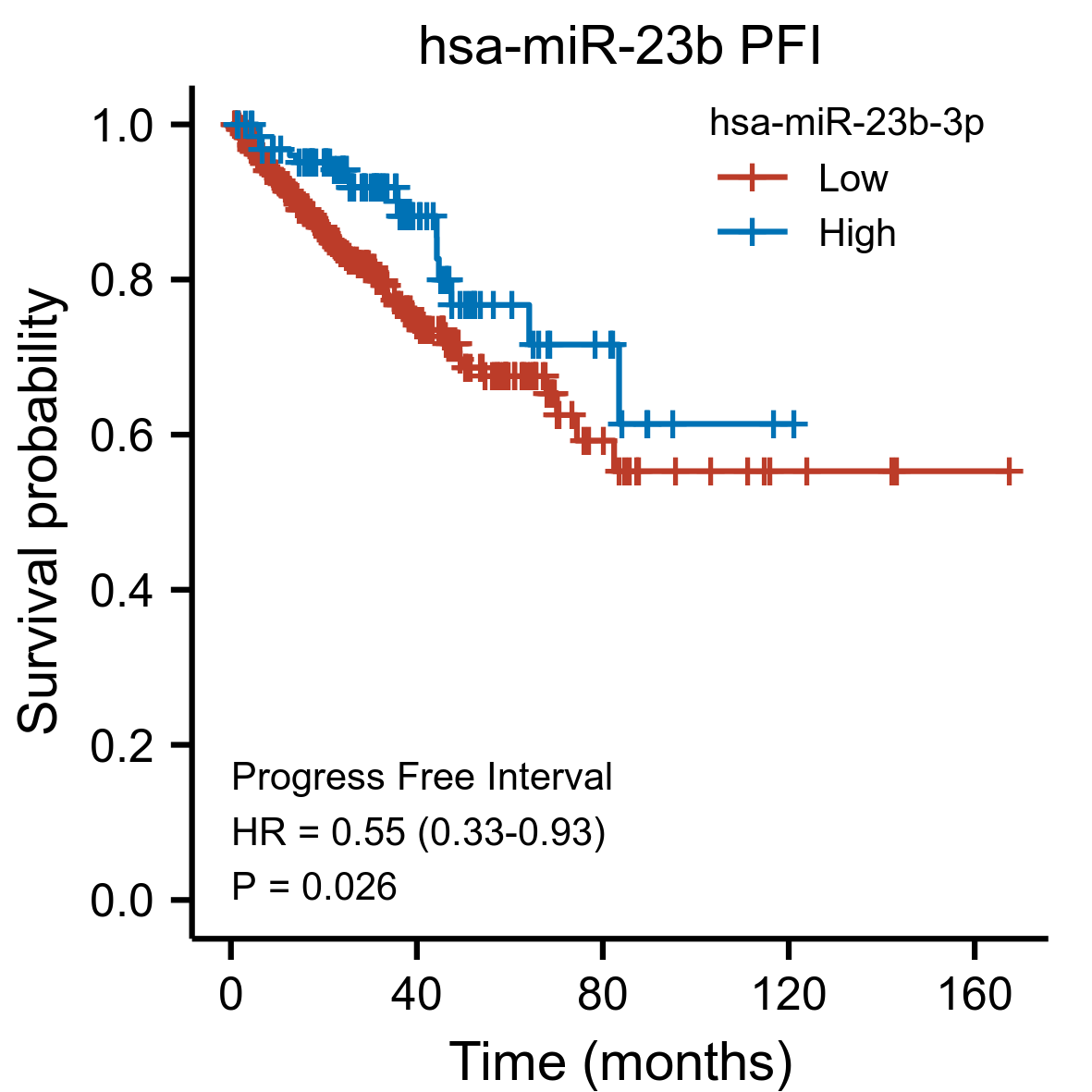

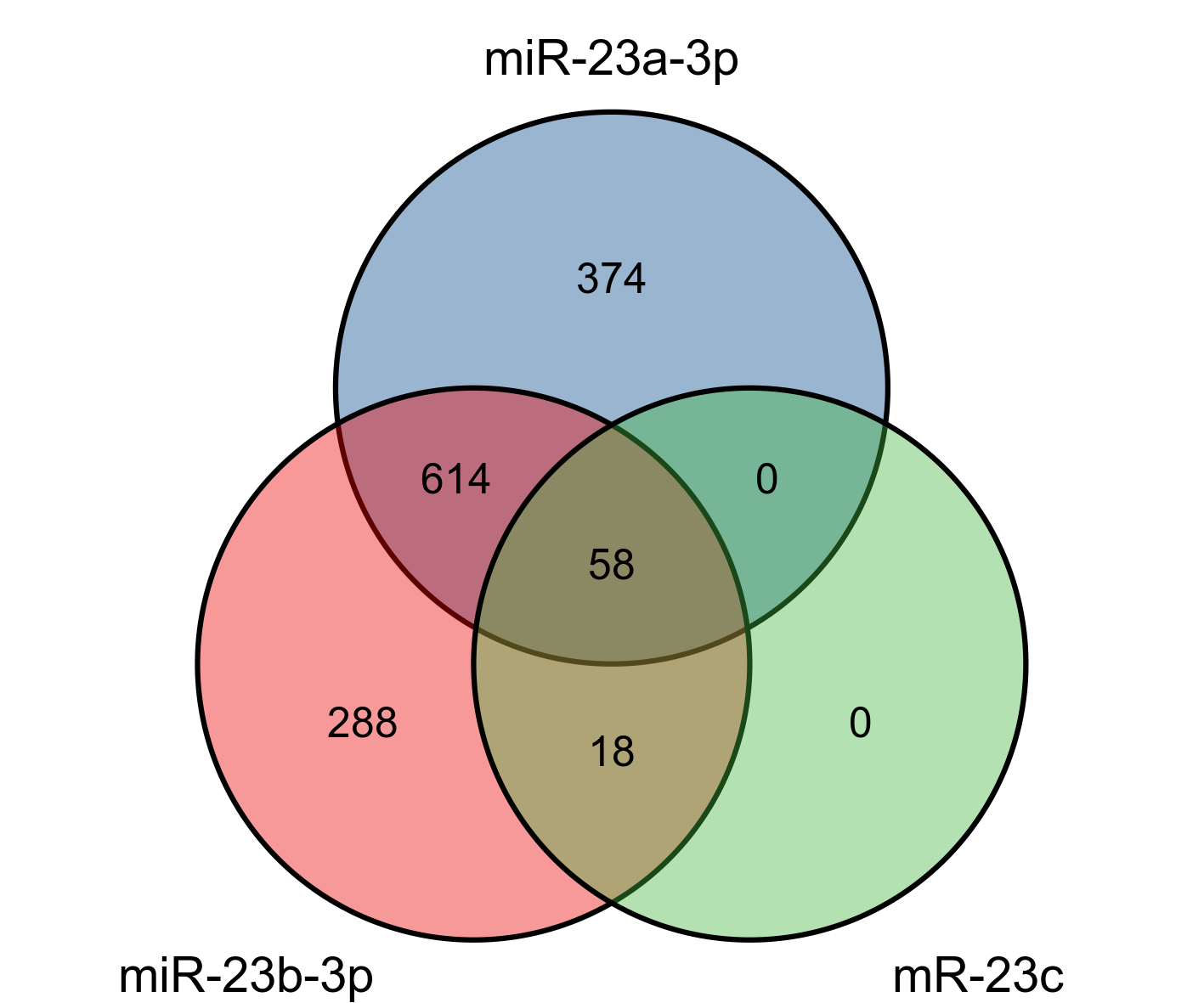

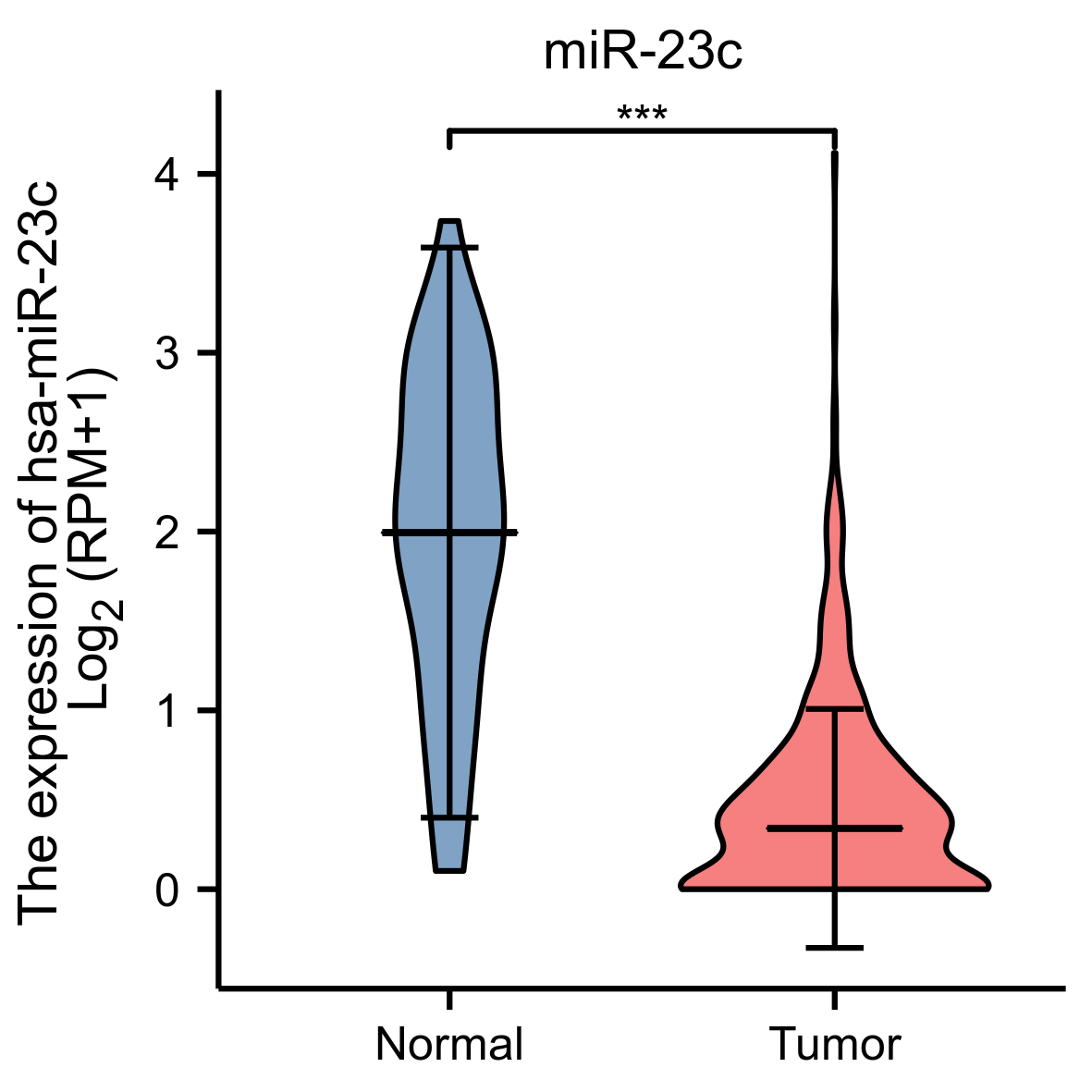

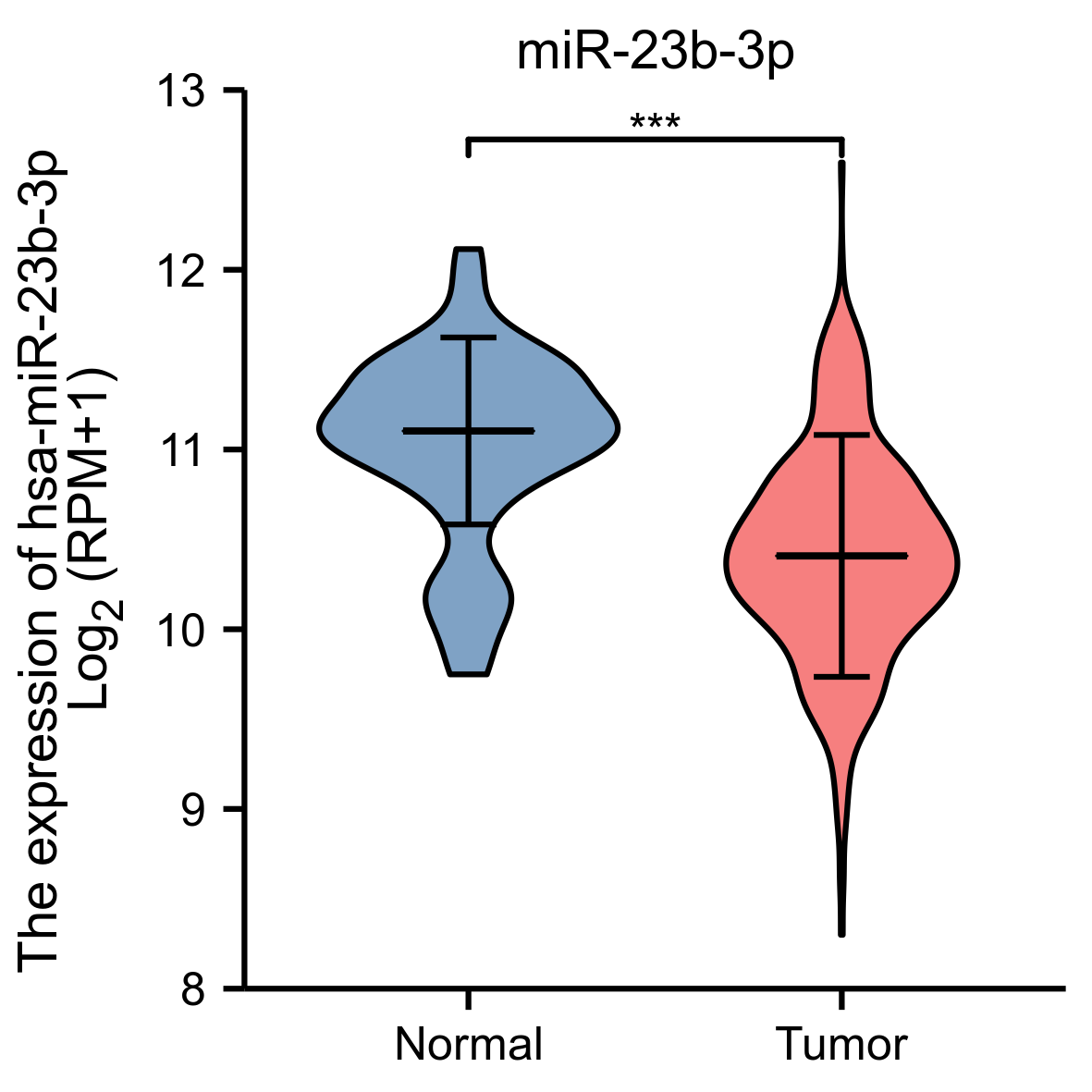

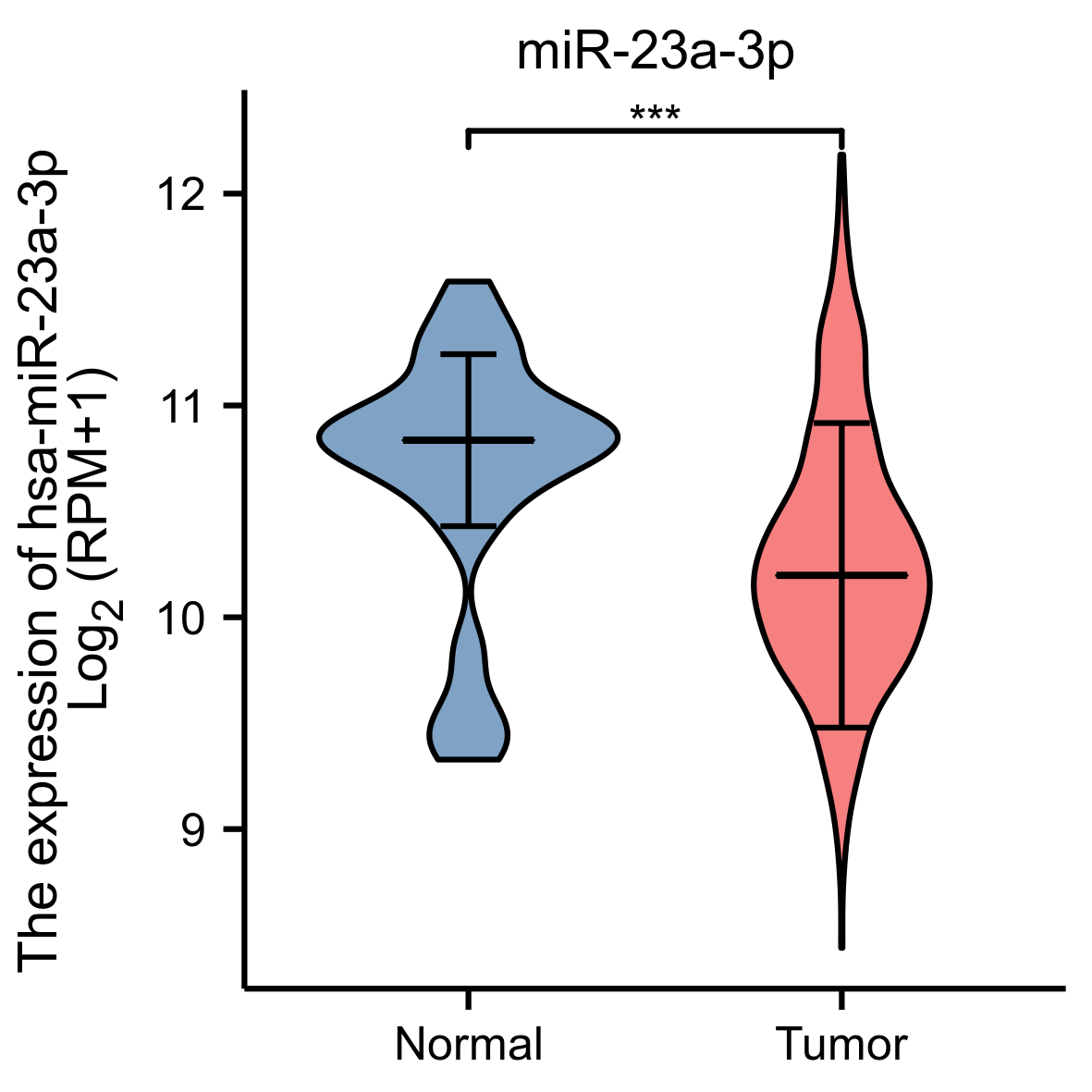


**A**

**B**

**C**

**D**

**E**

**F**

**G**

**H**


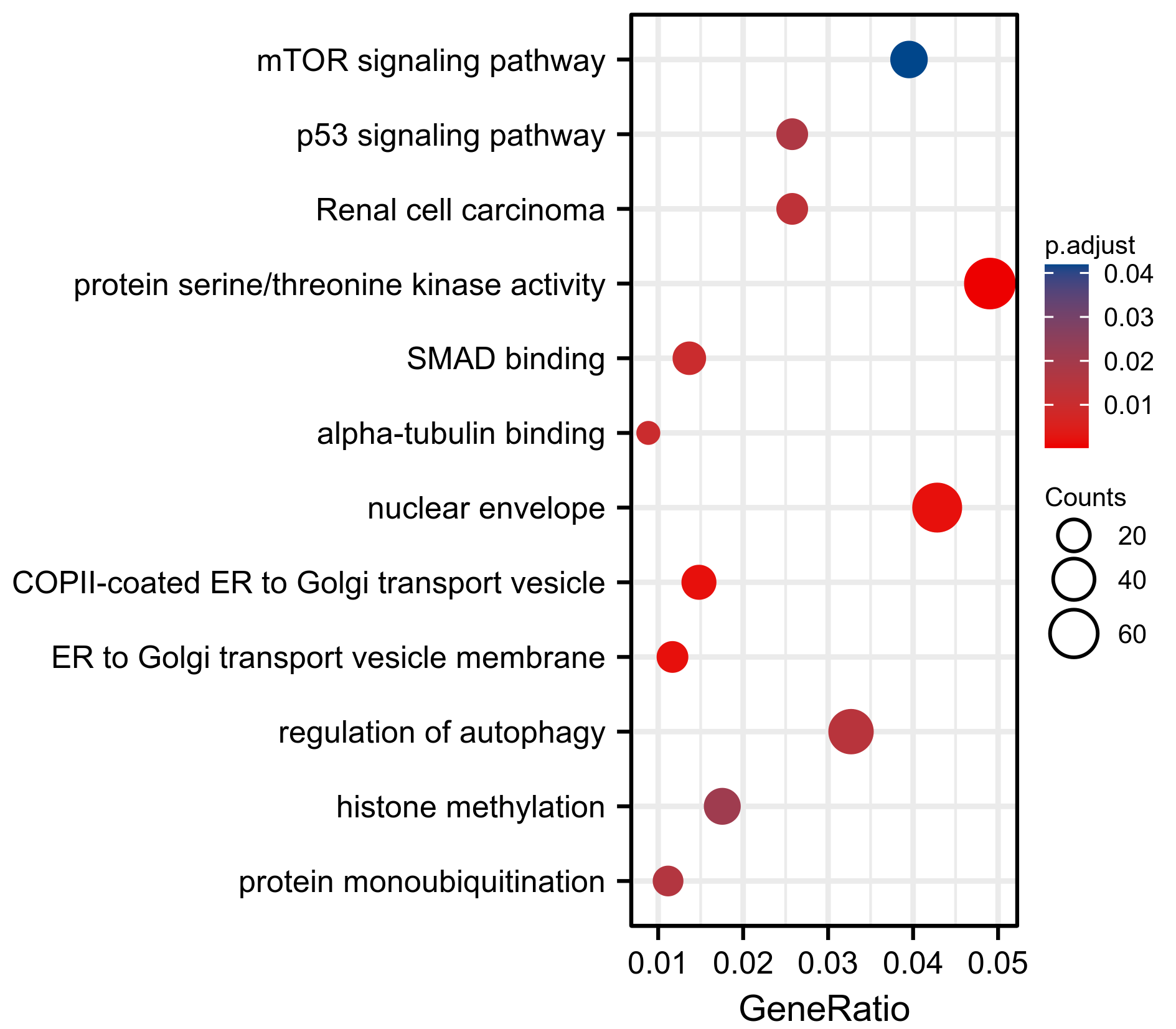


**(A-C)** Expression of miR-23a-3p, miR-23b-3p and miR-23c in adjacent normal prostate samples and prostate tumor tissue samples. *** indicates *p* < 0.001, by Wilcoxon rank sum test. **(D-F)** KM plots indicating the correlation between expression of miR-23a-3p, miR-23b-3p and miR-23c and PFI of PC patients, by one-way Cox regression test. **(G)** Venn diagram indicating the potential target genes of miR-23a-3p, miR-23b-3p and miR-23c. **(H)** GO/KEGG analysis of combined target genes of miR-23a-3p, miR-23b-3p and miR-23c.

**Supplementary Figure 4. Validation of transfection efficiency.**


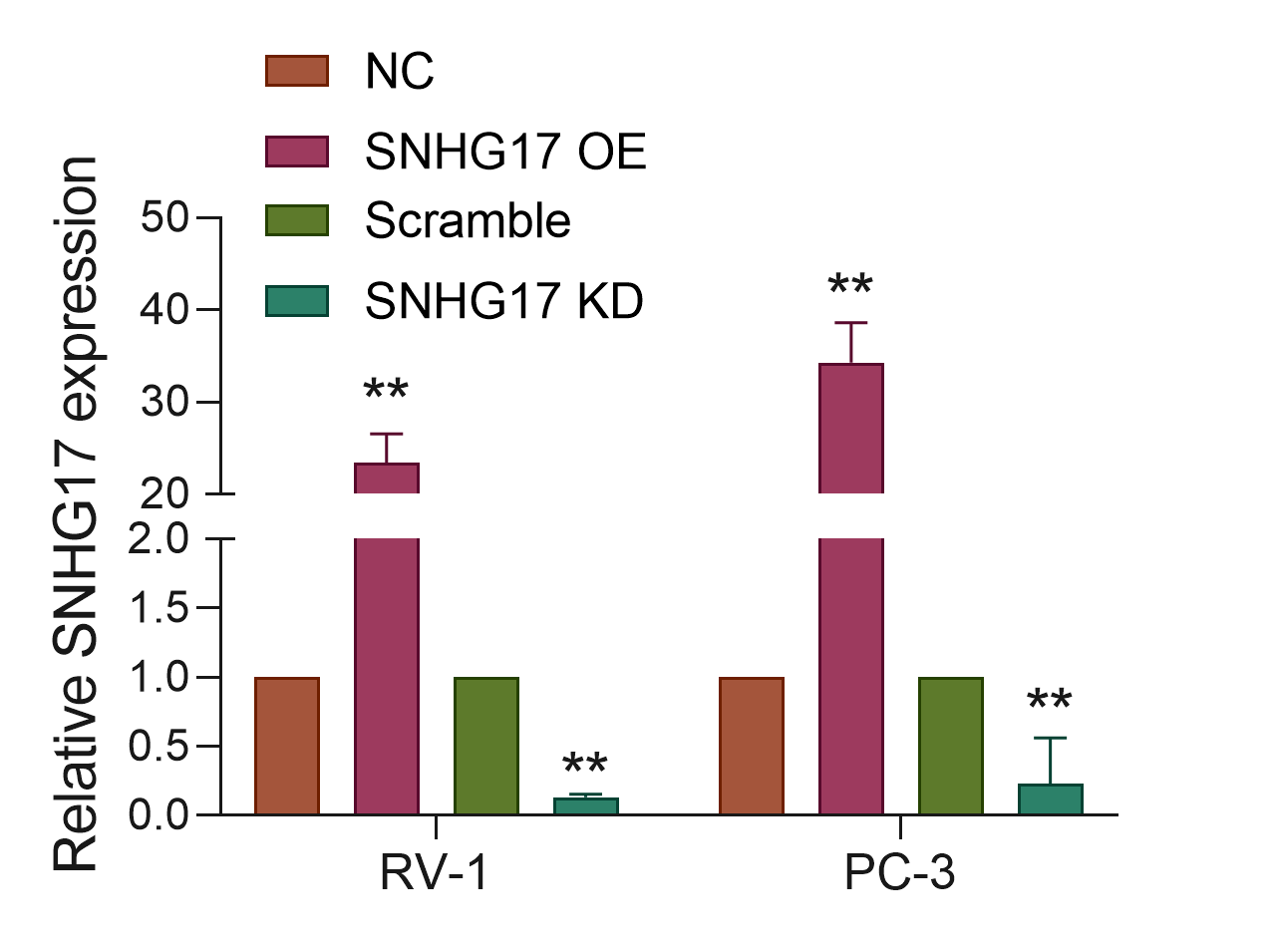

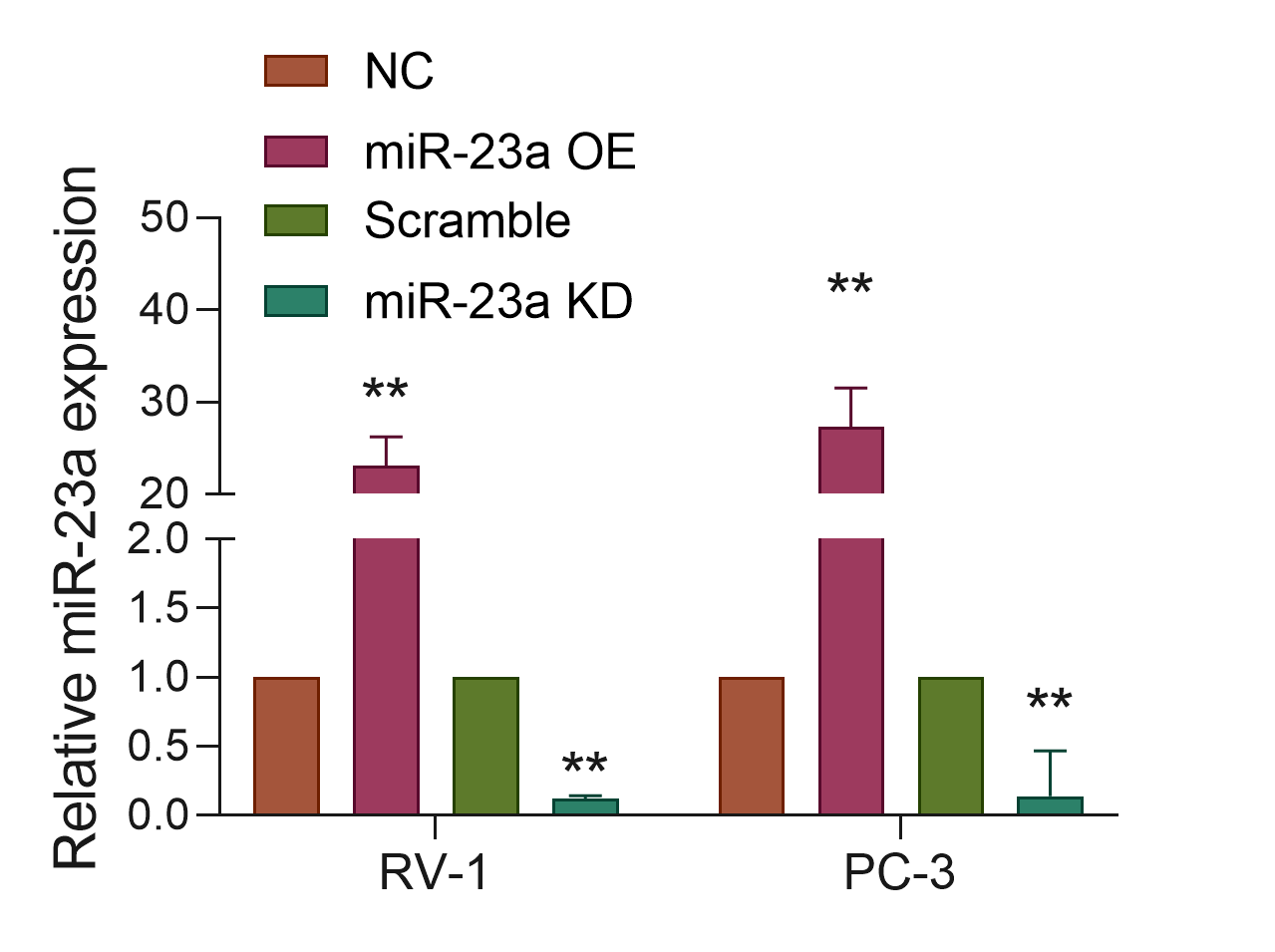

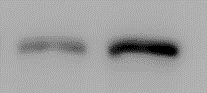

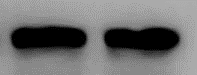


OTUB1

Actin

NC

OTUB1 OE

**A**

**B**

**C**

(A, B) Transfection efficiency of Knockdown or overexpression of SNHG17 and miR-23a-3p in RV-1 and PC-3 cells was assessed by qRT-PCR. (C) Transfection efficiency of overexpression of OTUB1 in RV-1 cells by western blot. ** indicates *p* < 0.01.

**Supplementary Figure 5. Correlation between UBE2M/OTUB1 expression and pathological features of PC and the related biological processes and pathways.**


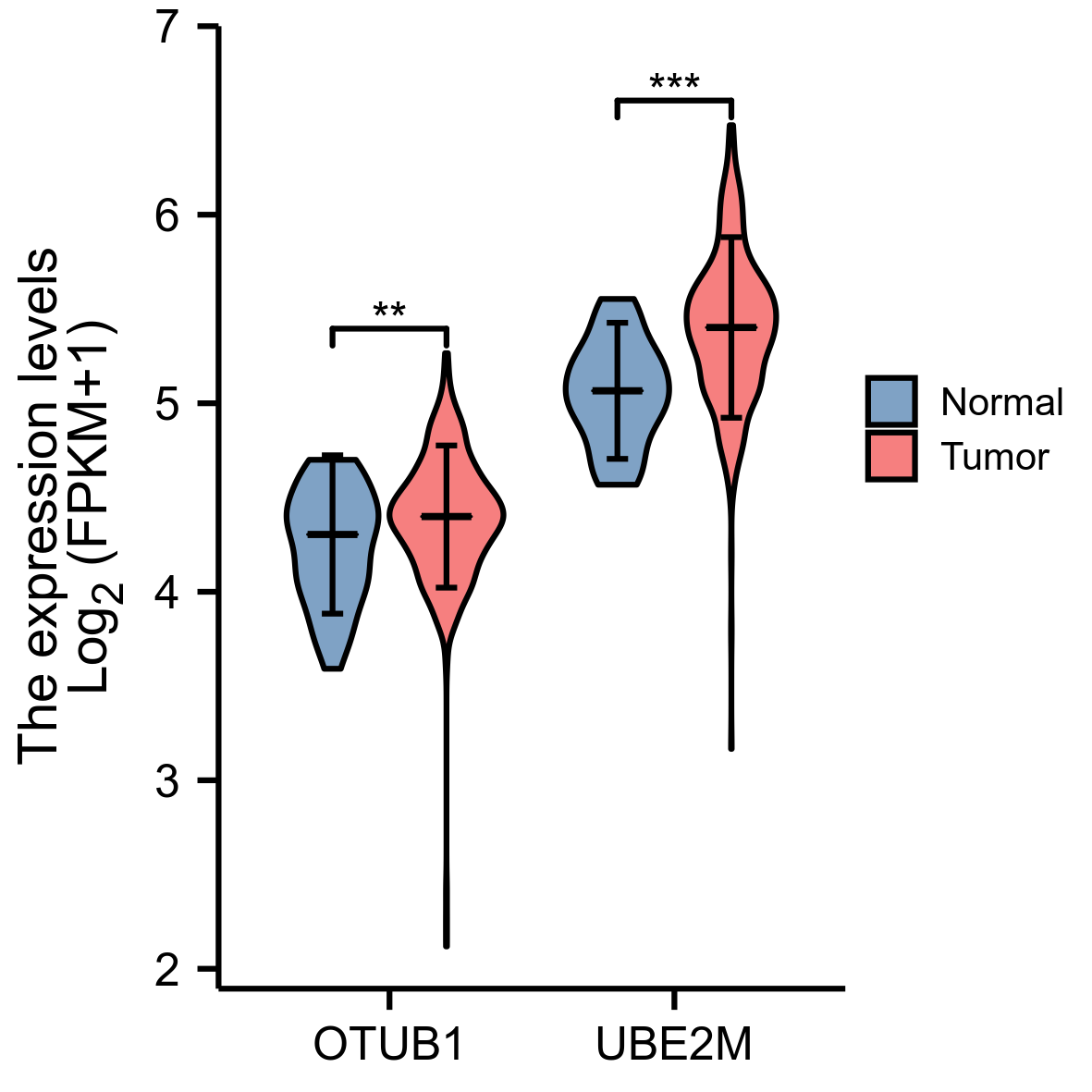

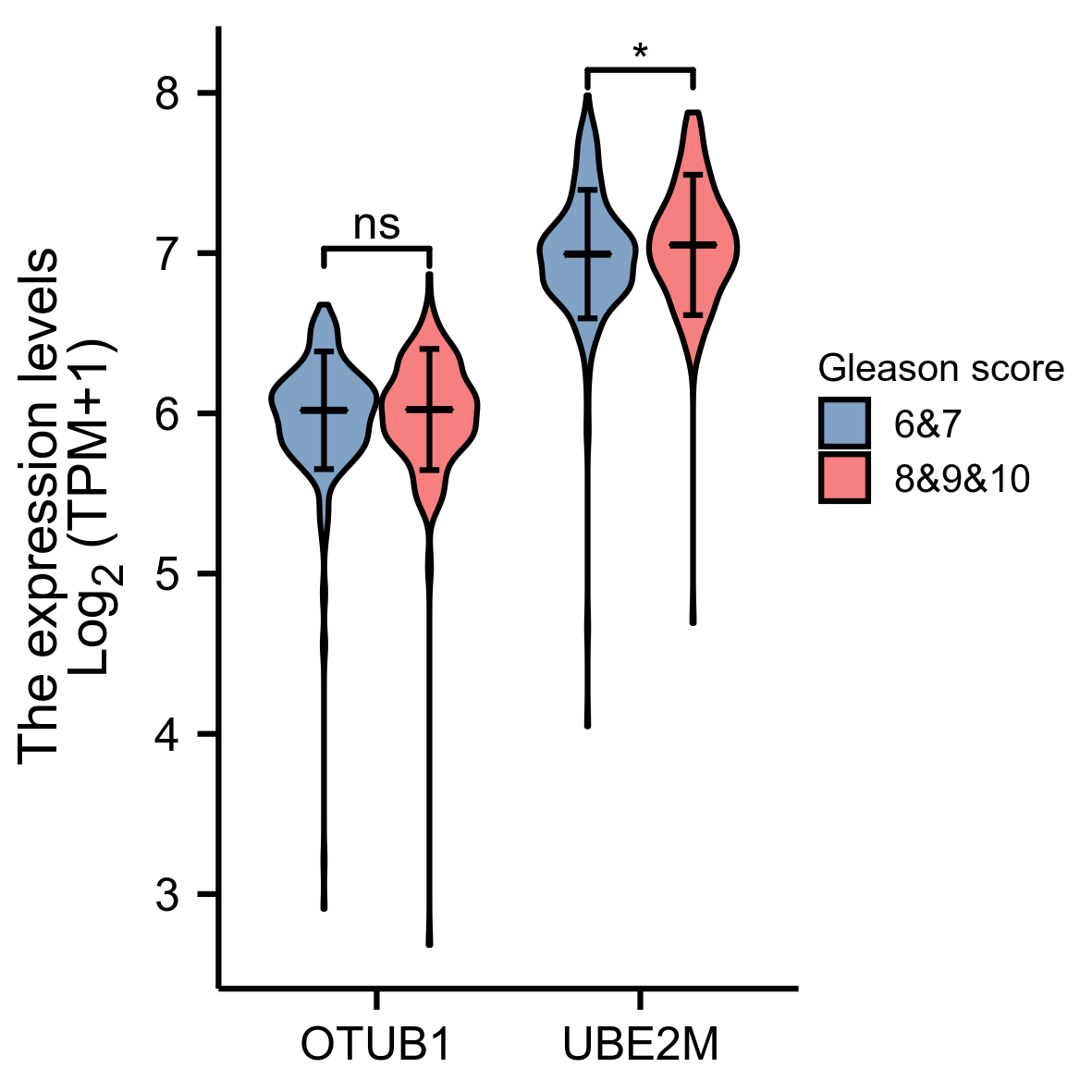

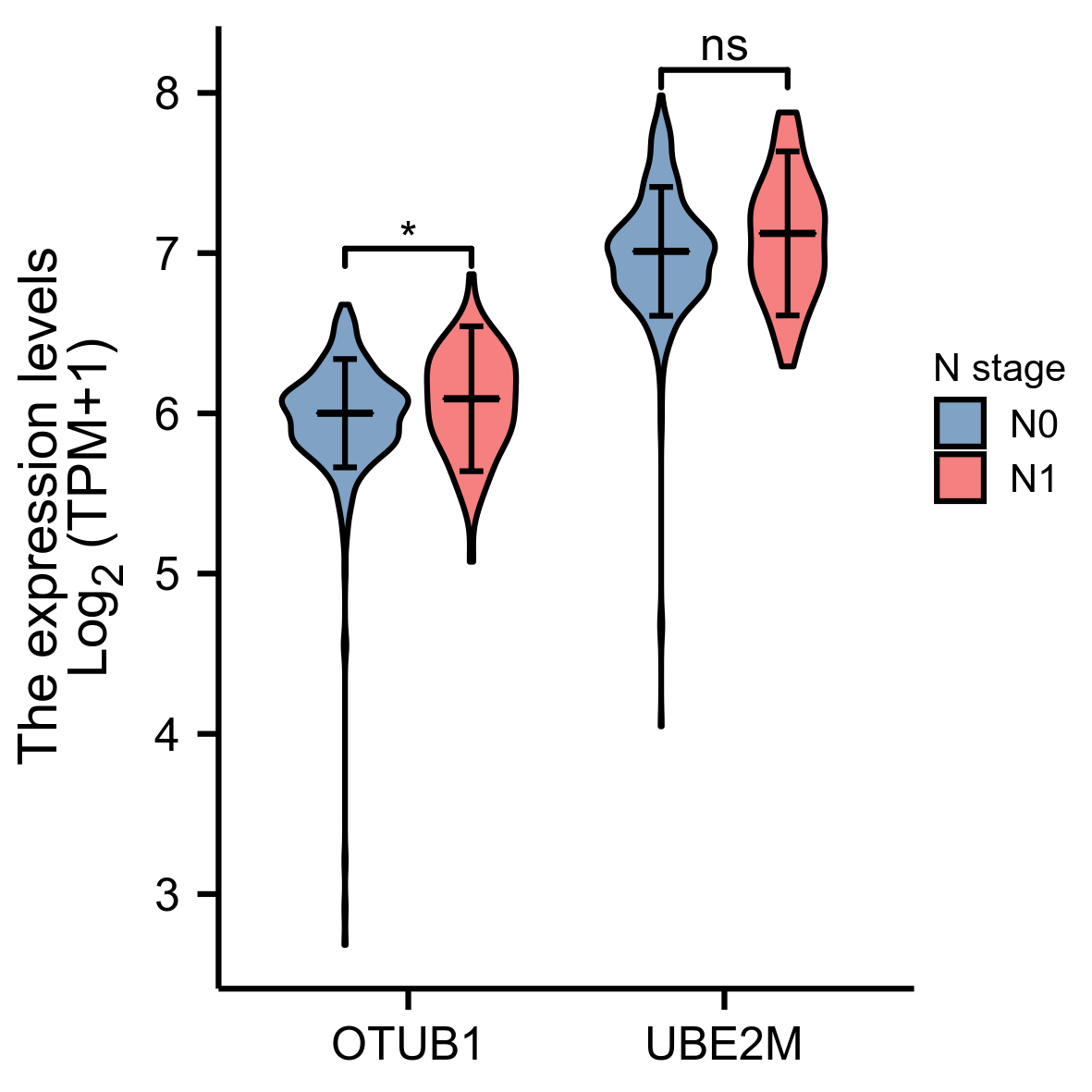


**A**

**B**

**C**


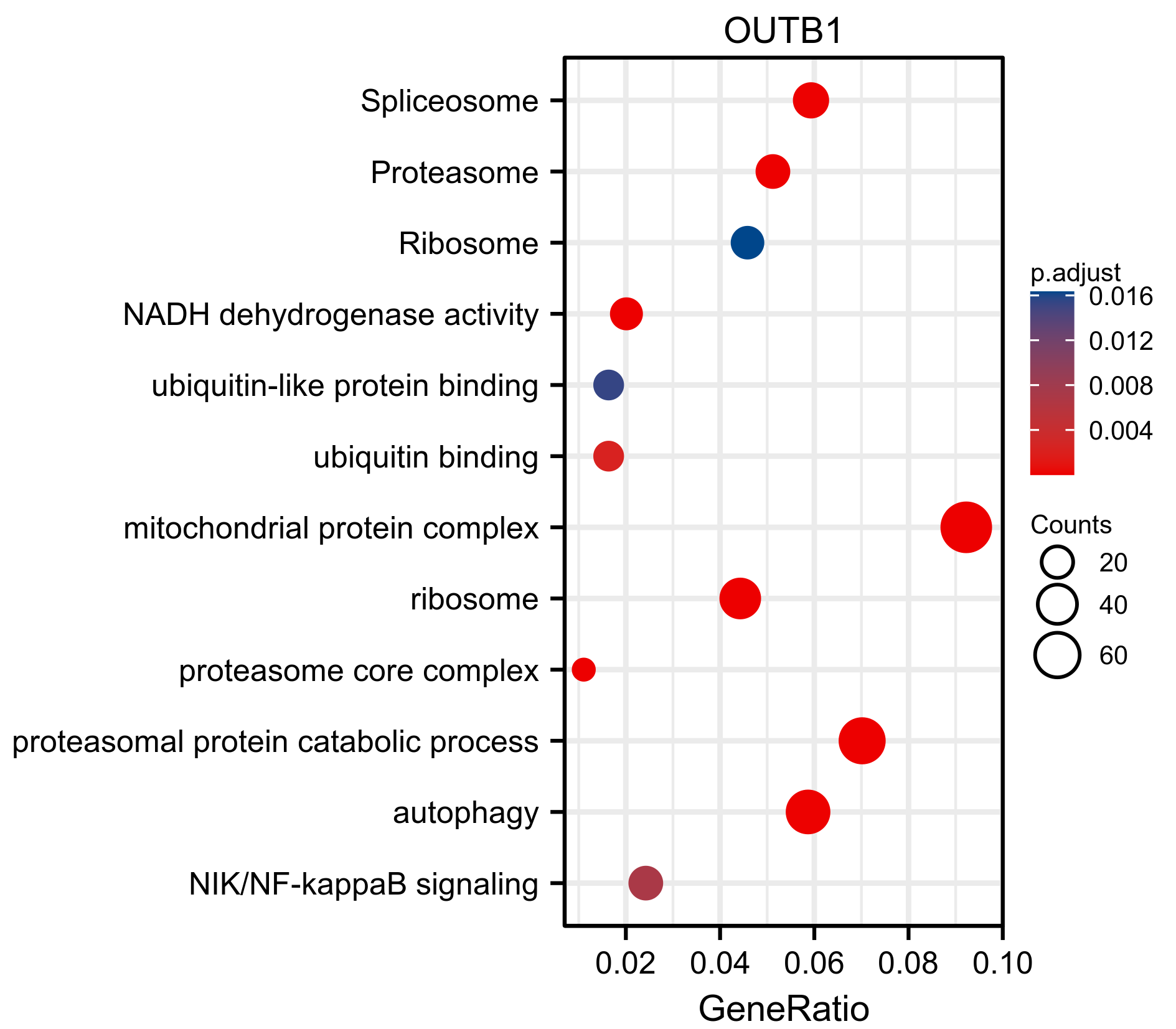

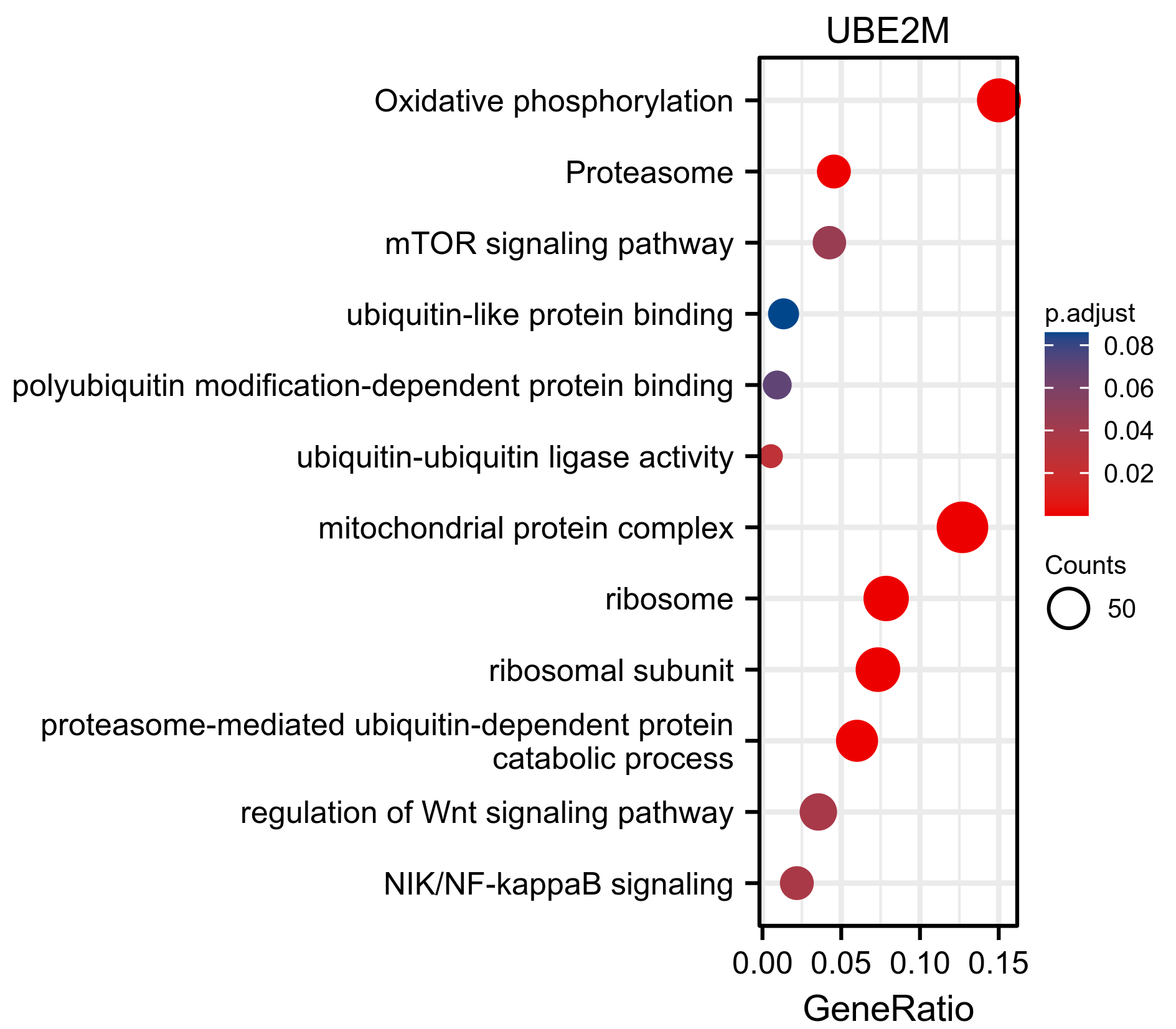


**F**

**G**


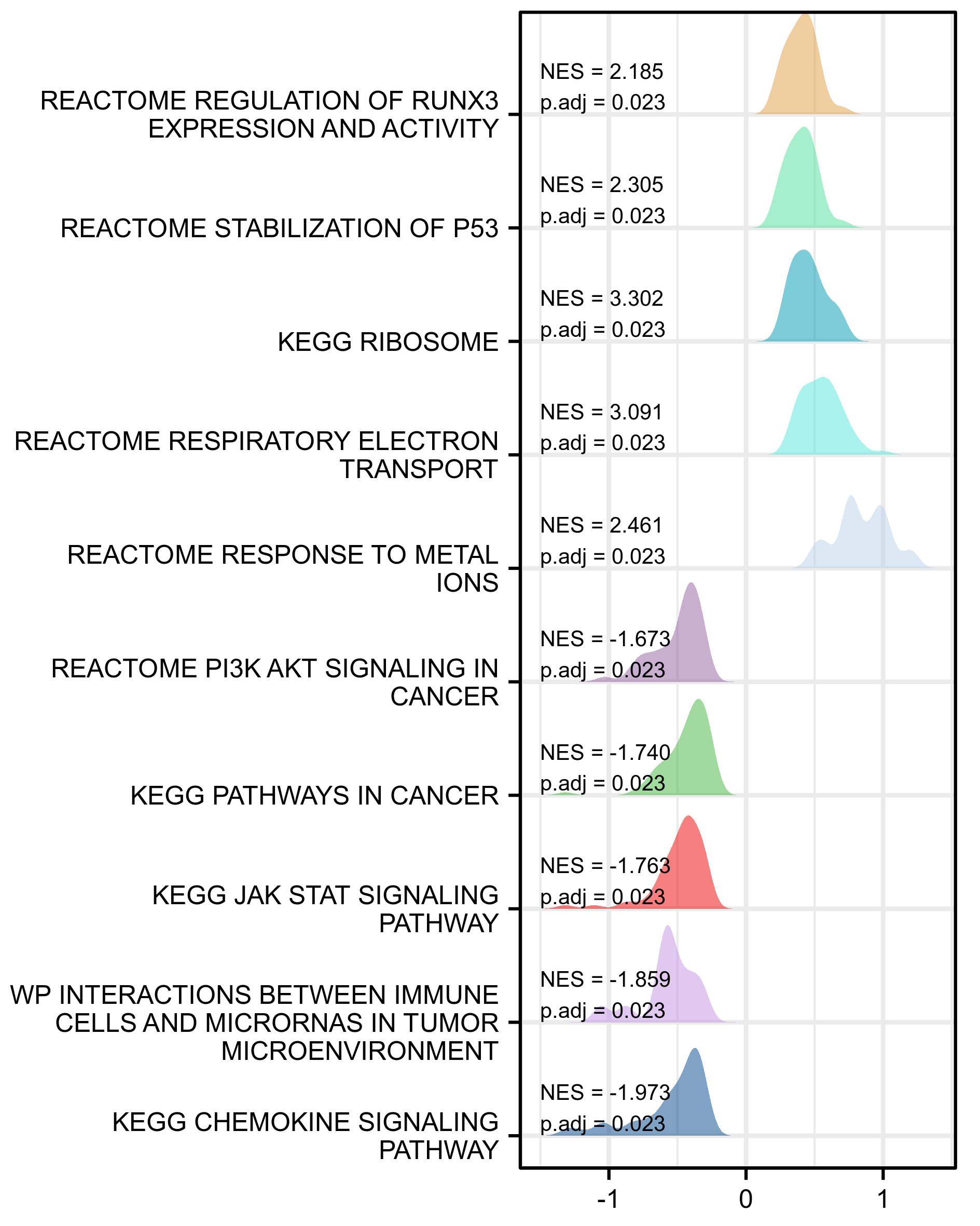

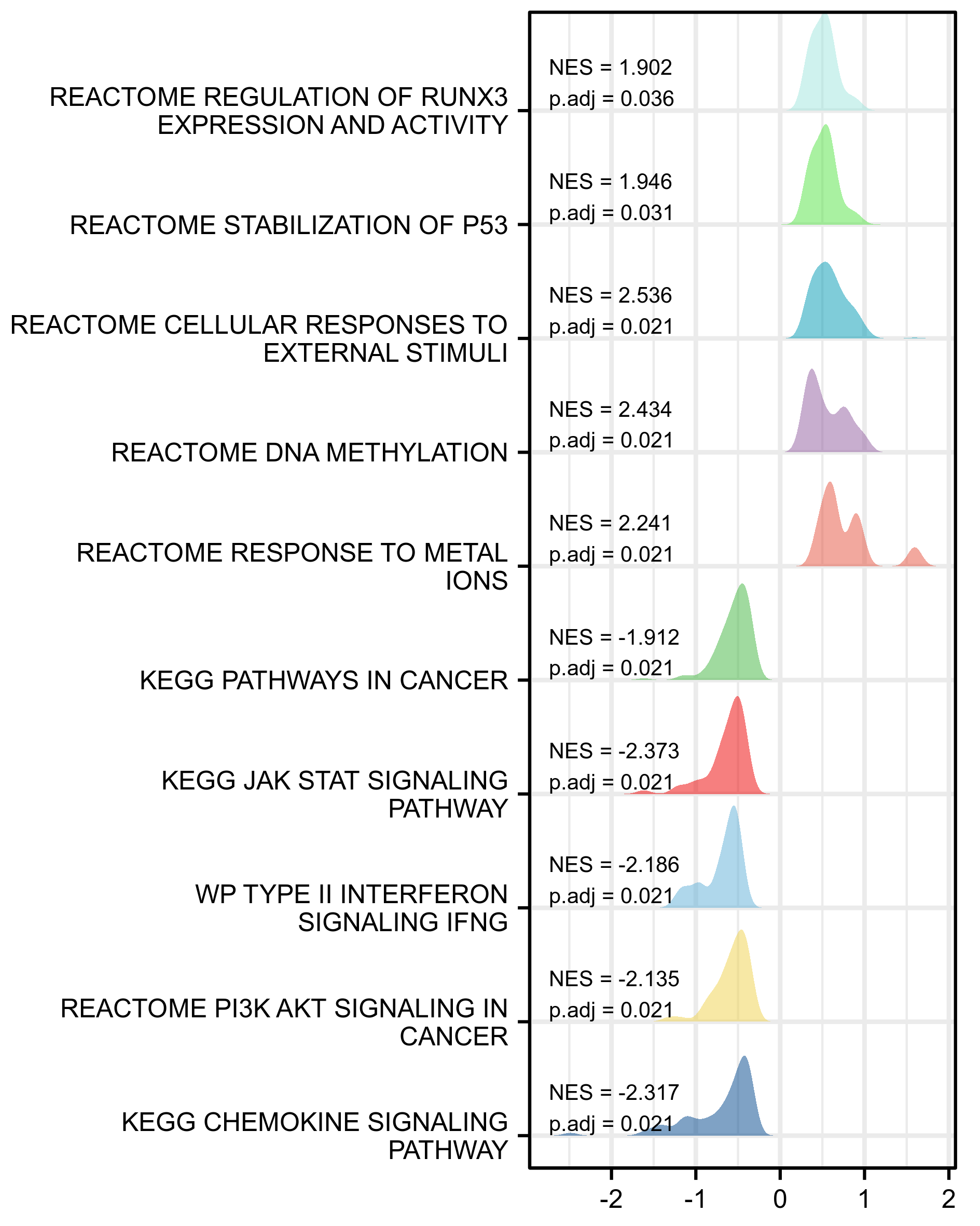


**D**

**E**

UBE2M

OTUB1

**(A)** Analysis of UBE2M/OTUB1 expression between adjacent normal prostate samples and prostate tumor tissue samples. **(B)** Analysis of UBE2M/OTUB1 expression between tumors with low Gleason’s score (6 & 7) and high Gleason’s score (8 &9 & 10). ns indicates not significant, * indicates *p* < 0.05, ** indicates *p* < 0.01, *** indicates *p* < 0.001. **(C)** UBE2M/OTUB1 expression between tumors with or without lymph node metastasis. **(D, E)** Wave plots indicating the enriched pathway of UBE2M and OTUB1 in prostate cancer. NES = normalize enrichment score. **(F, G)** GO/KEGG analysis of UBE2M and OTUB1 in prostate cancer.


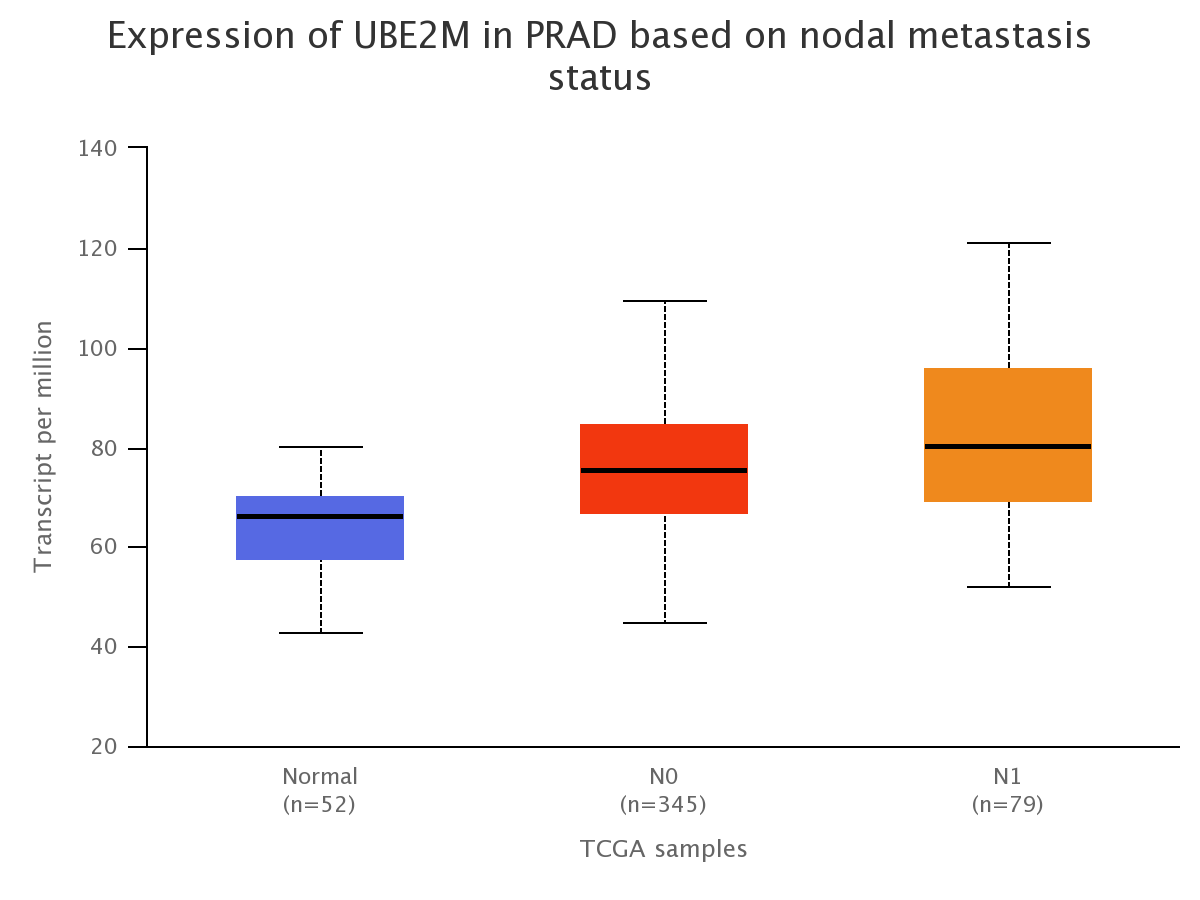

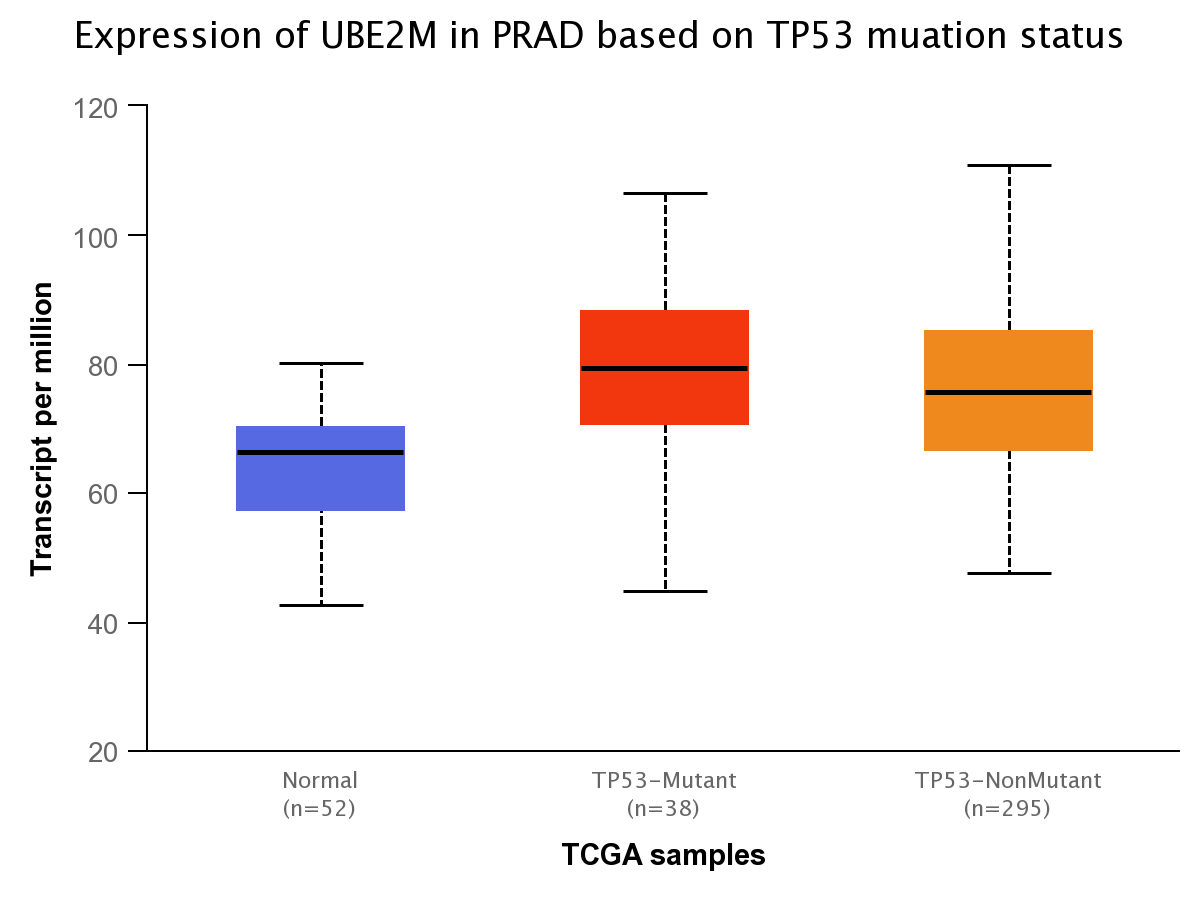

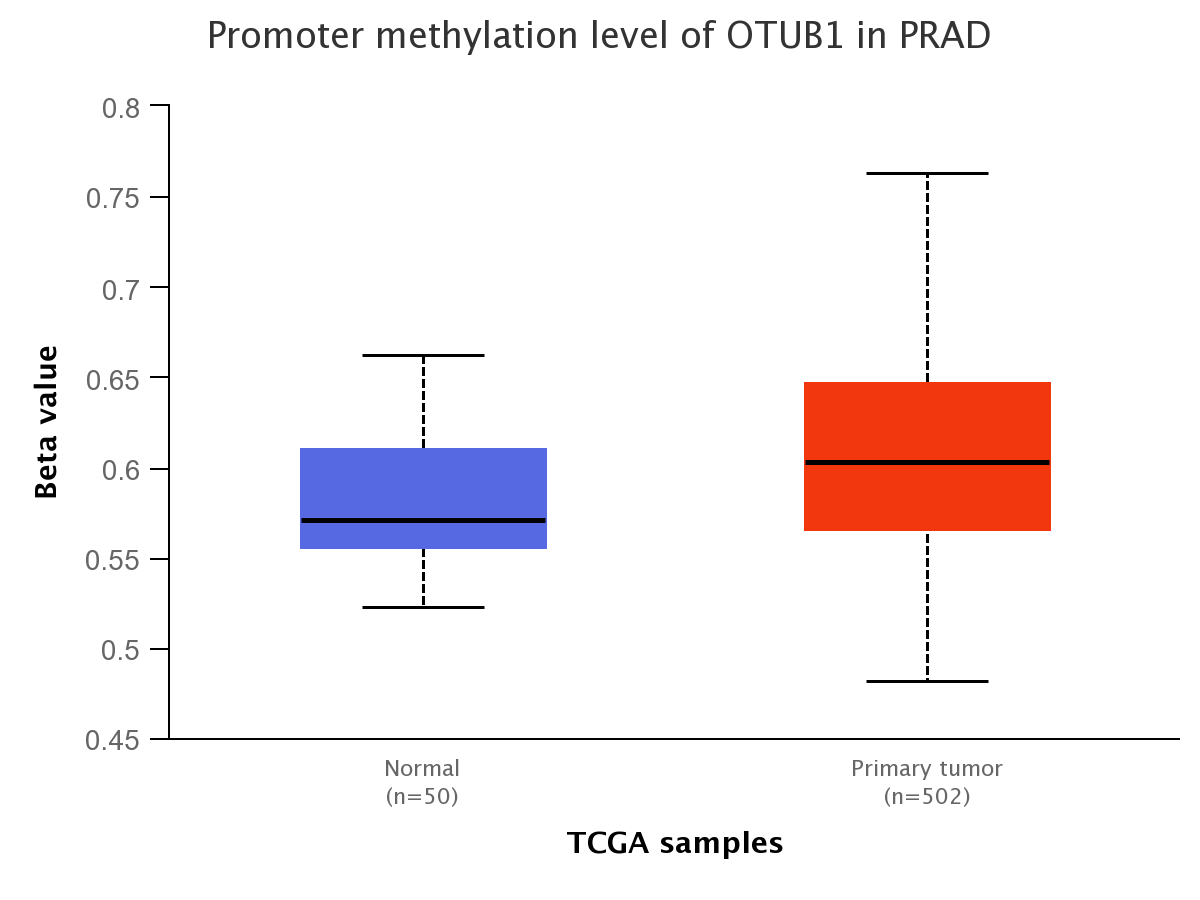

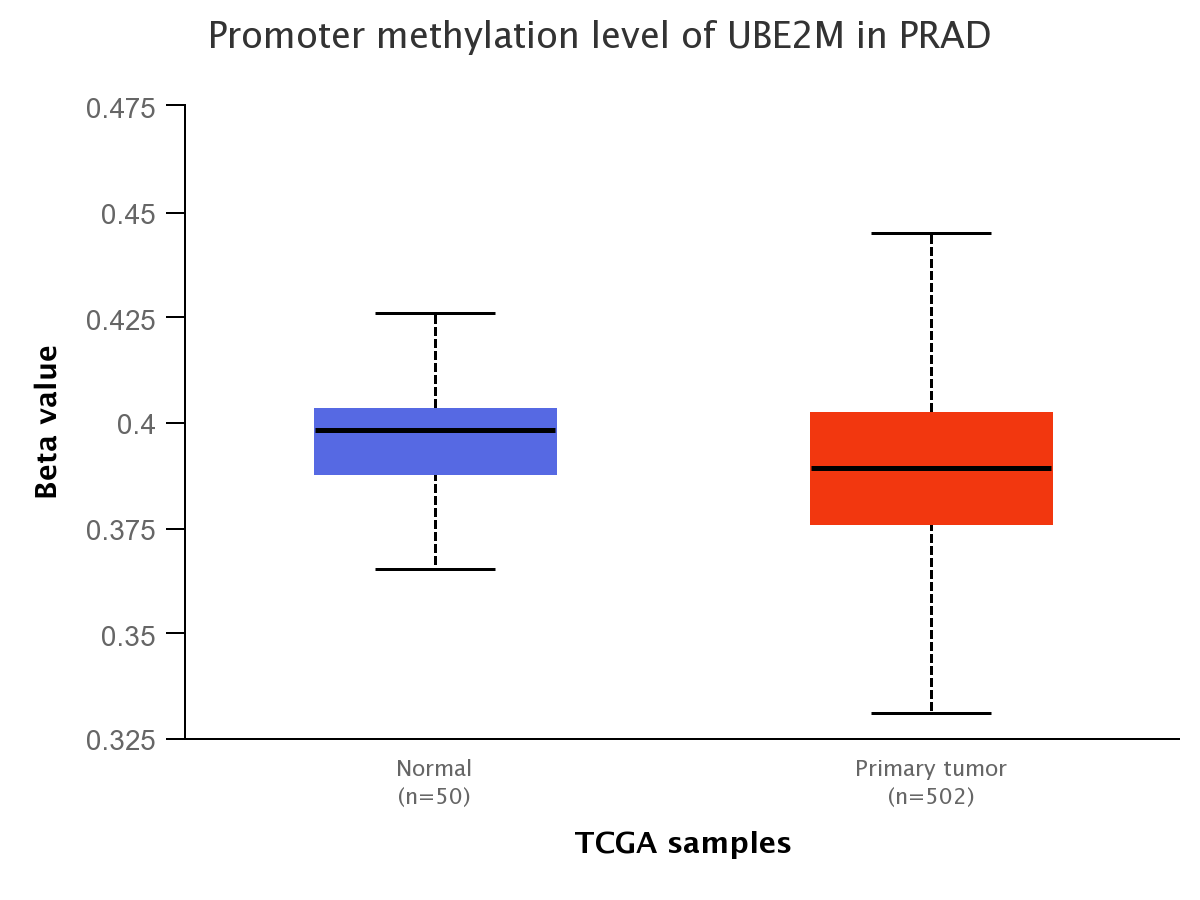

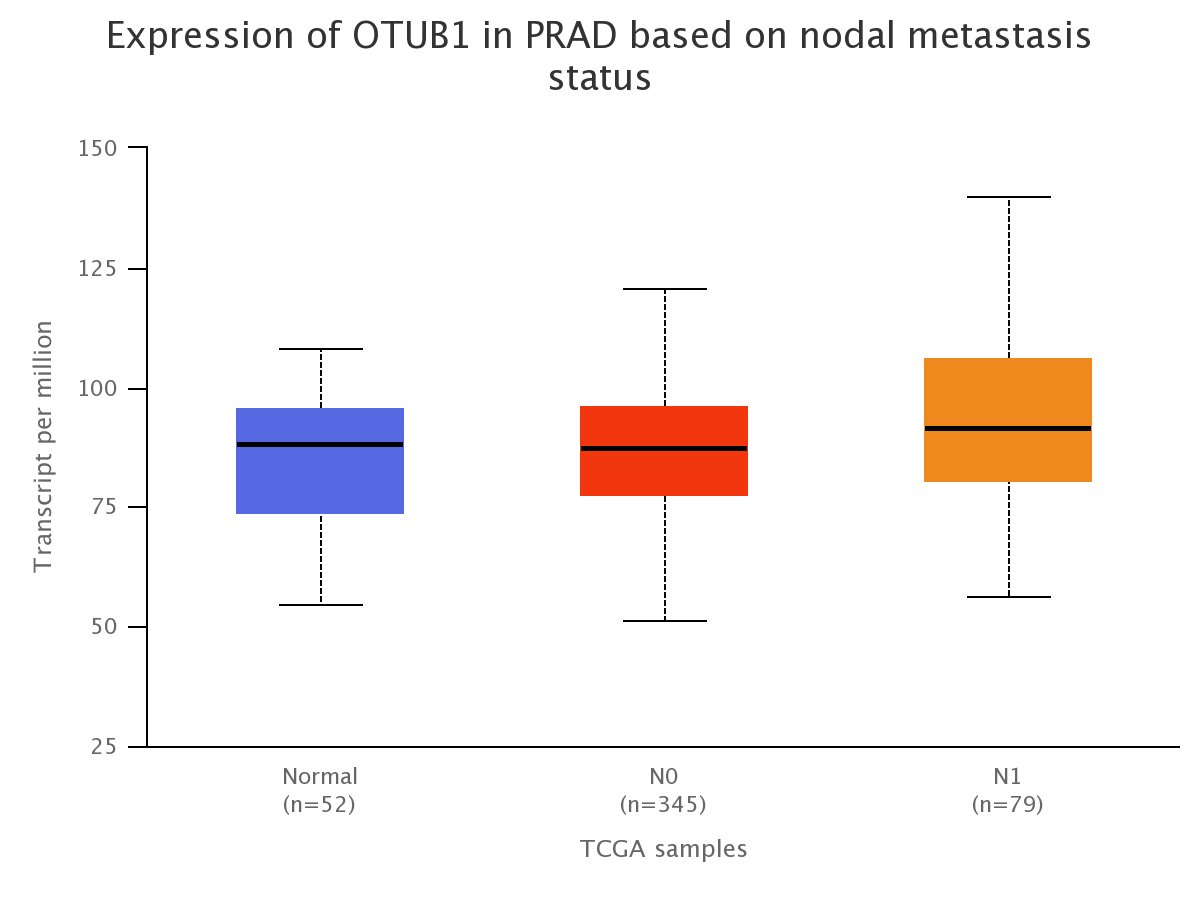

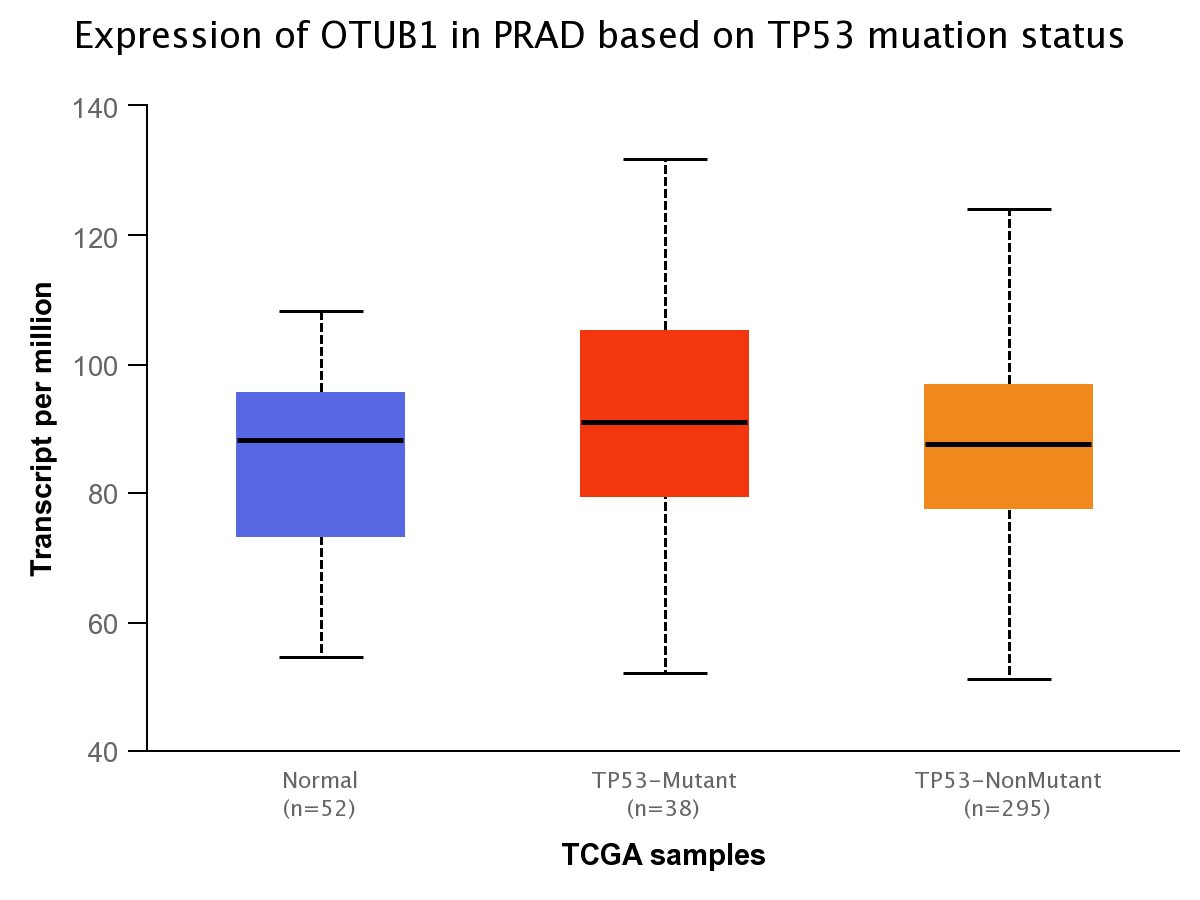


**A**

**B**

**C**

**D**

**E**

**F**

***

***

**

***

***

ns

***

ns

**

**

*

***

**Supplementary Figure 6. Relationship between UBE2M/OTUB1 expression and lymph node metastasis and TP53 mutation status, and analysis of promoter methylation of UBE2M/OTUB1, in prostate cancer.**

**(A, B)** Relationship between UBE2M/OTUB1 expression and lymph node metastasis in PC. **(C, D)** Relationship between UBE2M/OTUB1 expression and TP53 mutation status in PC. **(E, F)** Promoter methylation level of UBE2M/OTUB1 in prostate cancer. ns indicates not significant, * indicates *p* < 0.05, ** indicates *p* < 0.01, *** indicates *p* < 0.001.


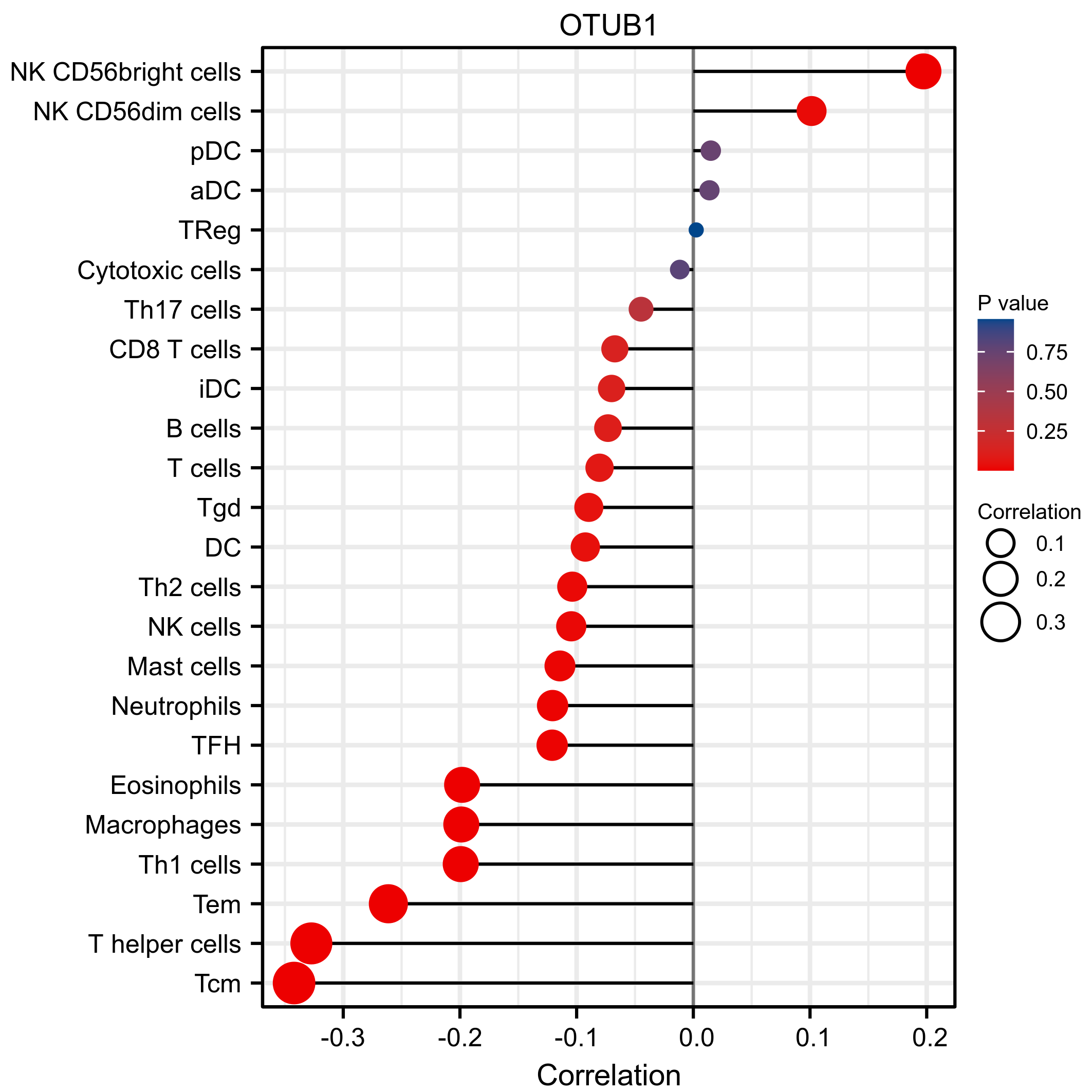

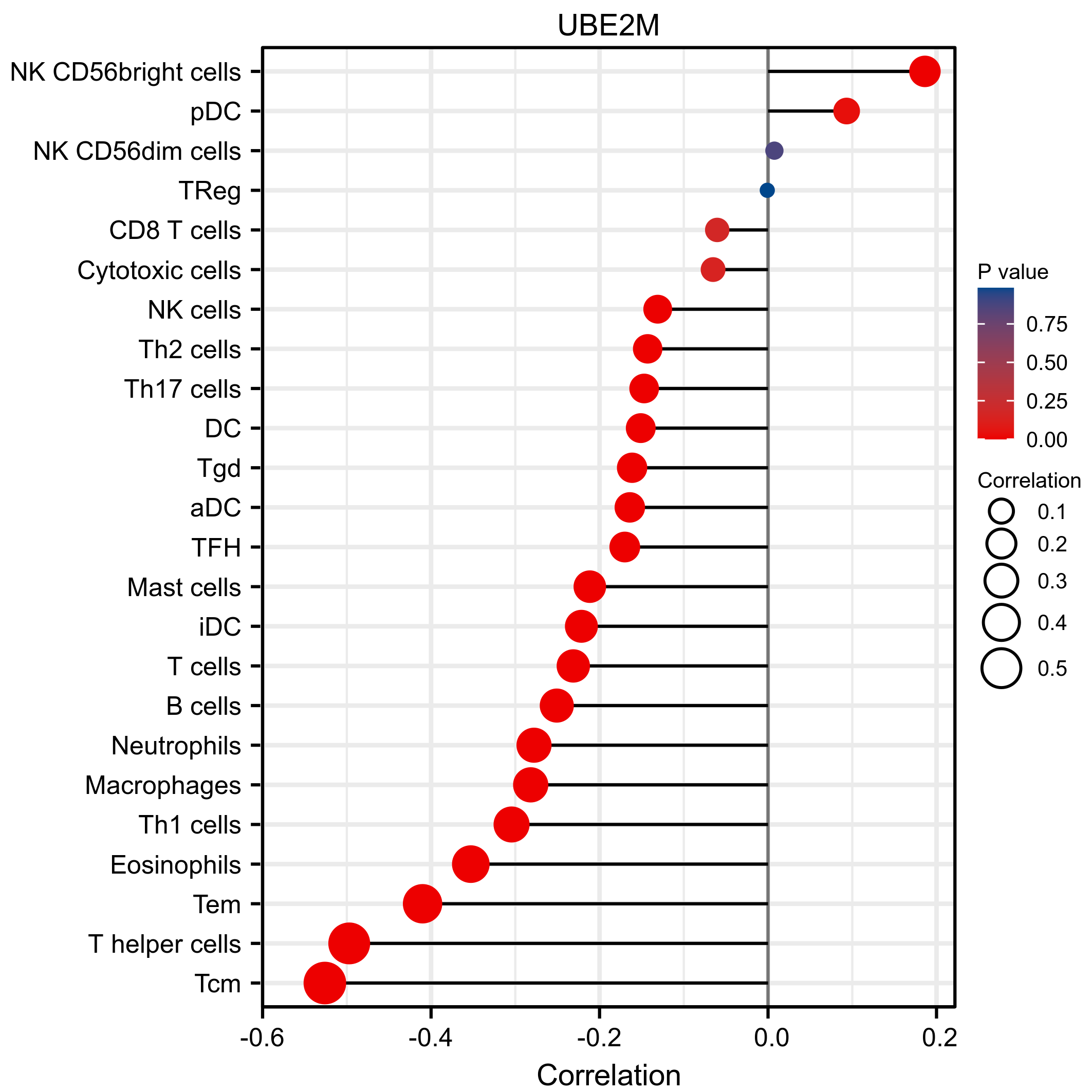

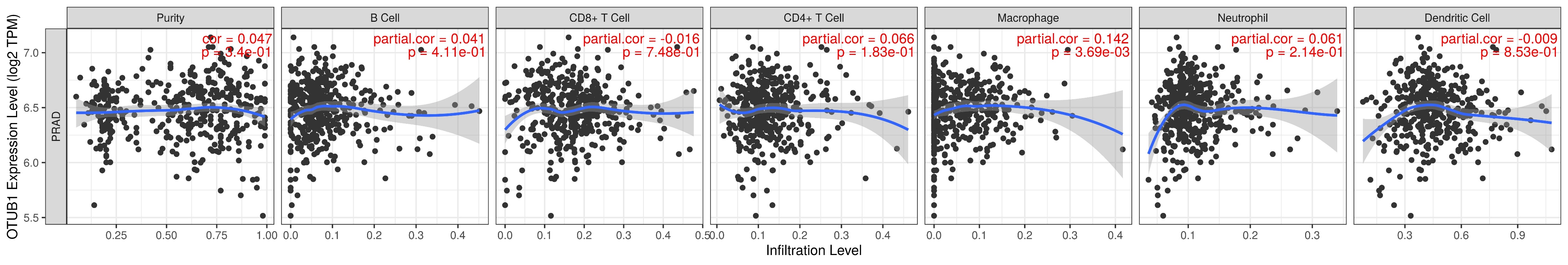

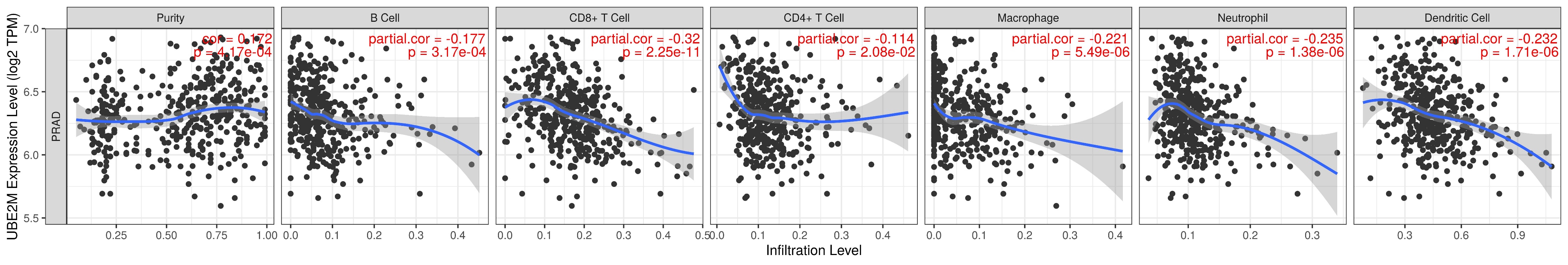


UBE2M

OTUB1

**A**

**B**

**C**

**D**

**Supplementary Figure 7. Correlation between UBE2M/OTUB1 and infiltration level of immune cells in PC.**

**(A)** Correlation between UBE2M/OTUB1 and immune infiltration in PC by ssGSEA method. **(B)** Correlation between UBE2M/OTUB1 and infiltration in six immune cells in PC by TIMER.


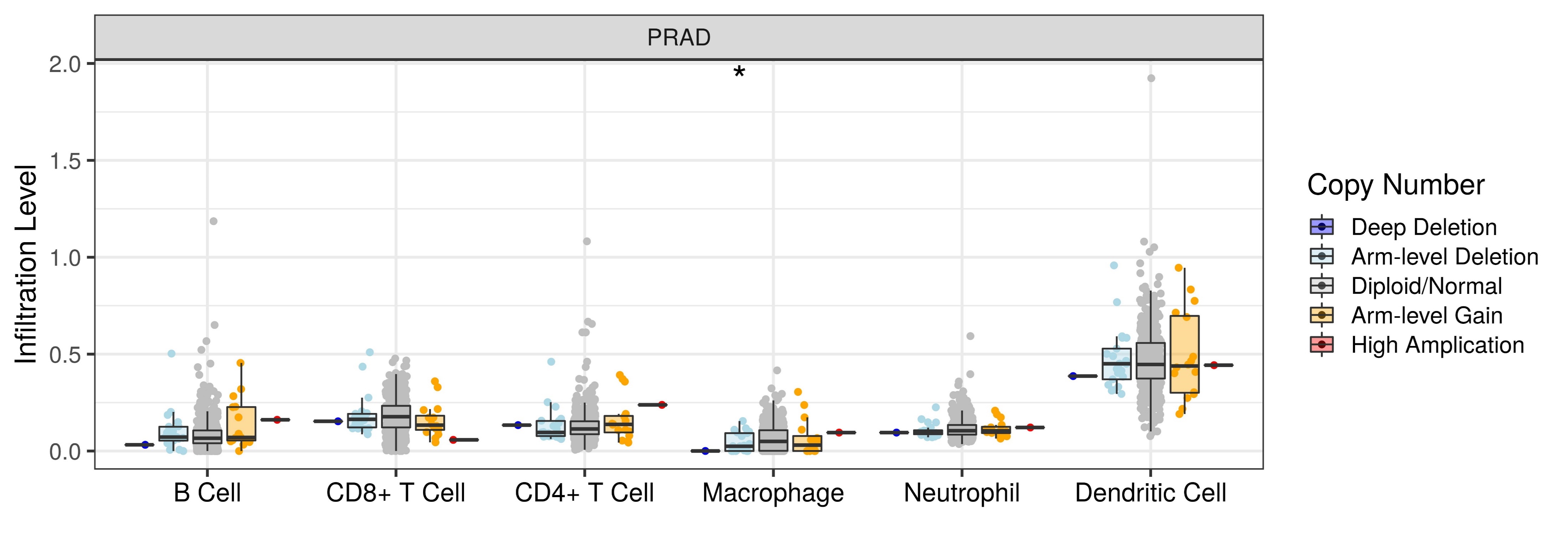

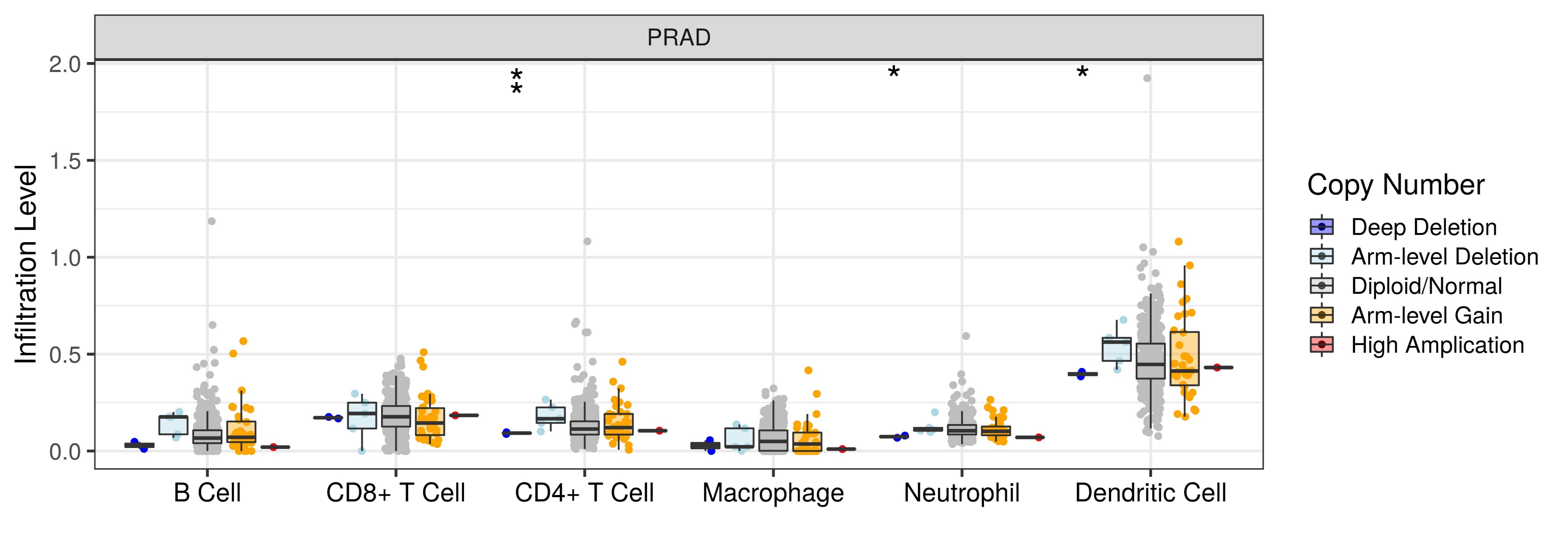


UBE2M

OTUB1

**A**

**B**

**Supplementary Figure 8. Relationship between somatic mutation of UBE2M/OTUB1 and immune infiltration in prostate cancer.**

**(A, B)** Relationship between somatic mutation of UBE2M/OTUB1 and immune infiltration in prostate cancer by SCNA analysis. * indicates *p* < 0.05, ** indicates *p* < 0.01.
